# SUMO E3 ligase Mms21 prevents spontaneous DNA damage induced genome rearrangements

**DOI:** 10.1371/journal.pgen.1007250

**Published:** 2018-03-05

**Authors:** Jason Liang, Bin-zhong Li, Alexander P. Tan, Richard D. Kolodner, Christopher D. Putnam, Huilin Zhou

**Affiliations:** 1 Ludwig Institute for Cancer Research, University of California School of Medicine, San Diego, La Jolla, California, United States of America; 2 Departments of Chemistry and Biochemistry, University of California School of Medicine, San Diego, La Jolla, California, United States of America; 3 Department of Cellular and Molecular Medicine, University of California School of Medicine, San Diego, La Jolla, California, United States of America; 4 Moores-UCSD Cancer Center, University of California School of Medicine, San Diego, La Jolla, California, United States of America; 5 Institute of Genomic Medicine, University of California School of Medicine, San Diego, La Jolla, California, United States of America; 6 Department of Medicine, University of California School of Medicine, San Diego, La Jolla, California, United States of America; Columbia University, UNITED STATES

## Abstract

Mms21, a subunit of the Smc5/6 complex, possesses an E3 ligase activity for the Small Ubiquitin-like MOdifier (SUMO). Here we show that the *mms21-CH* mutation, which inactivates Mms21 ligase activity, causes increased accumulation of gross chromosomal rearrangements (GCRs) selected in the dGCR assay. These dGCRs are formed by non-allelic homologous recombination between divergent DNA sequences mediated by Rad52-, Rrm3- and Pol32-dependent break-induced replication. Combining *mms21-CH* with *sgs1Δ* caused a synergistic increase in GCRs rates, indicating the distinct roles of Mms21 and Sgs1 in suppressing GCRs. The *mms21-CH* mutation also caused increased rates of accumulating uGCRs mediated by breakpoints in unique sequences as revealed by whole genome sequencing. Consistent with the accumulation of endogenous DNA lesions, *mms21-CH* mutants accumulate increased levels of spontaneous Rad52 and Ddc2 foci and had a hyper-activated DNA damage checkpoint. Together, these findings support that Mms21 prevents the accumulation of spontaneous DNA lesions that cause diverse GCRs.

## Author summary

Chromosomal rearrangement is a hallmark of cancer. *Saccharomyces cerevisiae* Mms21 is an E3 ligase for **S**mall **U**biquitin like **MO**difer (SUMO), which has been shown to have a major role in preventing chromosomal rearrangement. Despite extensive studies about the function of Mms21 in regulating the repair of exogenously induced DNA damage, how Mms21, and its human ortholog *NSMCE2*, prevents spontaneous chromosomal rearrangement in unperturbed cells has been unknown. In this study, we provided genetic evidences supporting a novel role of Mms21 in preventing the accumulation of spontaneous DNA breaks, which are likely caused by defective DNA replication, without appreciably affecting how they are repaired. Our findings highlight the central role of faithful DNA replication in preventing spontaneous chromosomal rearrangement, and further suggest that the study of the role of Mms21 dependent sumoylation in DNA replication could yield important insights into how the SUMO pathway prevents chromosomal rearrangement in human disease.

## Introduction

The Small Ubiquitin-like MOdifier (SUMO) regulates many biological processes through its covalent attachment to lysine residues on target proteins via a cascade of an E1-activating enzyme (Aos1-Uba2 in *Saccharomyces cerevisiae*), an E2-conjugating enzyme Ubc9, and one of several SUMO E3 ligases [1]. Three mitotic SUMO E3 ligases (Siz1, Siz2 and Mms21/Nse2) have been identified in *S*. *cerevisiae*, and these enzymes control substrate-specific sumoylation *in vivo*. Siz1 and Siz2, two paralogs of the PIAS family SUMO E3 ligases [2], catalyze the bulk of intracellular sumoylation [3,4], while the SUMO E3 ligase Mms21 has fewer known substrates [5,6]. This mitotic SUMO pathway is essential for cell viability in *S*. *cerevisiae*; individual deletions of *AOS1*, *UBA2*, or *UBC9*, and combined inactivation of all three mitotic SUMO E3 ligases causes lethality [5]. In contrast, sumoylation of proteins by Mms21 is not necessary for viability in the presence of Siz1 and Siz2 in *S*. *cerevisiae* nor do mice require the SUMO E3 ligase activity of the mouse Mms21 ortholog NSMCE2 [7], indicating some redundancy between mitotic E3 ligases.

Mms21 is an integral subunit of the Smc5/6 complex and it is essential for cell viability like other subunits in this complex [8]. The Smc5/6 complex belongs to the evolutionarily conserved Structural Maintenance of Chromosomes (SMC) family proteins and acts in maintaining chromosome integrity [9]. Loss of the Mms21 SUMO E3 ligase activity does not affect cell viability but causes aberrant increases in homologous recombination (HR) intermediates, increased sister chromatid exchange (SCE) and accumulations of gross chromosomal rearrangements (GCRs) in *S*. *cerevisiae* [4,10–13]. Consistent with this, mutations in human *NSMCE2/MMS21* cause increased SCE [14] and have been recently linked to DNA replication and/or repair defects and primordial dwarfism [15].

How sumoylation by Mms21 acts to suppress the accumulation of HR intermediates and GCRs is not known. These phenotypes could be attributed to a failure in resolving HR intermediates and/or an elevated incidence of DNA lesions that are repaired by HR. These phenotypes, however, are reminiscent of those of cells lacking the Sgs1 helicase [10,16]. Sgs1, the *S*. *cerevisiae* ortholog of the human BLM helicase that is deficient in patients with Bloom syndrome, has well-documented roles in resolving HR intermediates as well as participating in resection of DNA double strand breaks (DSBs) [17–19]. The similarity between the phenotypes caused by *sgs1Δ* and *mms21* E3 ligase-defective mutations raises the possibility that Mms21 and Sgs1 might function together to regulate or prevent HR [10]. In support of this model, two recent studies showed that sumoylation of Sgs1/BLM by Mms21/NSMCE2 prevents the accumulation of aberrant HR intermediates induced by DNA alkylation damage [20,21]. However, the roles of Sgs1 and Mms21 in preventing spontaneous genome rearrangements have not been investigated in sufficient detail, although mutations affecting each cause increased accumulation of GCRs [4,22].

In contrast, several lines of evidence suggest that Mms21 and Sgs1 function in separate pathways that act to maintain genome stability. The *sgs1* mutations that eliminate DNA damage-induced sumoylation of Sgs1 by Mms21 do not cause appreciable sensitivity to DNA damaging agents [20,21], unlike that seen for *mms21* E3 ligase defective mutants and *sgs1Δ* mutants [5,10]. Similarly, combining mutations affecting *NSMCE2/MMS21* and *BLM/SGS1* caused synthetic growth defects and increased SCE in mouse B cells [7]. We previously demonstrated that Esc2, a protein containing two SUMO-like domains with an important role in genome maintenance [11,23], functions together with Mms21 in controlling intracellular sumoylation and suppressing GCRs [4]. Mutations affecting both *SGS1* and *ESC2* cause a synthetic growth defect and elevated gene conversion and joint-molecule formation in *S*. *cerevisiae* [24]. Moreover, several studies have suggested that the increased genome instability of *mms21* mutants might not be caused by a defect in DNA repair, in contrast to the known repair defects caused by *sgs1* mutations [17–19]. For example, the repair of meiotic DNA DSBs occurs with normal kinetics in *mms21* mutants [25]. In addition, the increased level of SCE in *nsmce2/mms21* mutant mice is not associated with an increase in 53BP1 foci, suggesting a lack of an obvious defect in DNA DSB repair [7]. Together these studies suggest that the genome maintenance functions of the Mms21-Esc2 pathway and Sgs1 might be different.

To gain insight into these questions, we performed a detailed study of the defects caused by the *mms21-CH* mutation, a SUMO E3 ligase-inactive allele of *MMS21* that results in C200A and H202A substitutions in the Mms21 SP-RING catalytic domain [4]. Our findings show that a diverse array of genome rearrangements accumulate in *mms21-CH* mutants, depending on specific DNA repair pathways available and the nature of genomic sequences involved in the formation of the GCRs observed. Collectively, these findings suggest that spontaneous DNA lesions accumulate in the *mms21-CH* mutant and initiate these genome rearrangements. We further show that Mms21 prevents spontaneous Pol32-dependent break induced replication (BIR) event, which is also dependent upon the Rrm3 DNA helicase and a subset of the DNA damage checkpoint, but does not involve resolution of recombination intermediates by Sgs1 and does not involve DNA damage-induced sumoylation of Sgs1.

## Results

### Genes in the *RAD52* epistasis group are required for the formation of duplication-mediated GCRs in *mms21-CH* mutant strains

**Fig 1:**
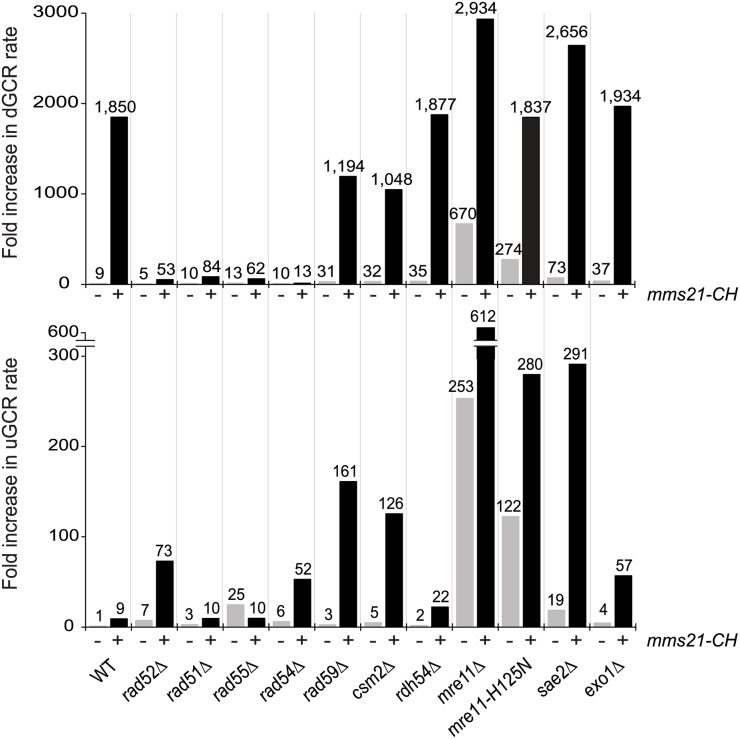
dGCR and uGCR rates caused by mutations of genes in the *RAD52* epistasis group and the *mms21-CH* mutation, a SUMO E3 ligase-null allele. The number above each bar indicates the fold change normalized to the uGCR rate of wild-type strain. Detailed results used to generate the bar graph are shown in S1 Table.

We previously showed that the *mms21-CH* mutation caused a substantial accumulation of GCRs selected in the duplication-mediated GCR (dGCR) assay (also called the *yel072w*::*CAN1/URA3* assay) [4]. In the dGCR assay, non-allelic HR between divergent homologous sequences on chromosome V and chromosomes IV, X, or XIV resulting in the formation of translocations dominate the GCRs selected in most HR-proficient strains [22,26] (S1 Fig). In contrast, the *mms21-CH* mutation caused only a modest increase in GCR rates in the unique sequence-mediated (uGCR) assay (also called the *yel068c*::*CAN1/URA3* assay) [4], which primarily selects for GCRs mediated by terminal deletions healed by *de novo* telomere additions and various types of micro- and non-homology mediated translocations [27] (S1 Fig). To explore this further, we combined the *mms21-CH* mutation with mutations affecting individual genes in the *RAD52* epistasis group in strains containing the dGCR assay or the uGCR assay. We then performed fluctuation analysis to measure the GCR rates of these single and double mutant strains (Fig 1 and S1 Table).

This analysis of the *RAD52* epistasis group of genes uncovered three main classes of genetic interactions. Class I mutations included deletions of the *RAD51*, *RAD52*, *RAD54* and *RAD55* genes required for HR [28]. In this case, deletion of each gene caused a drastic reduction of the increased dGCR rate caused by an *mms21-CH* mutation, indicating a requirement for HR in the formation of GCRs selected in the dGCR assay. Two of the Class I mutations, *rad51Δ* and *rad55Δ*, did not cause an increased uGCR rate when combined with the *mms21-CH* mutation, whereas two of the class I mutations, *rad52Δ* and *rad54Δ* (as well as a *rad59Δ* mutation; see below), caused an increased uGCR rate when combined with the *mms21-CH* mutation; this is consistent with previous observations that some HR pathways suppress GCRs selected in single copy sequence-mediated GCR assays such as the uGCR assay, presumably by promoting sister chromatid HR [22,29]. In contrast, deletion of *RDH54*, which encodes a Rad54 paralog with a role in meiotic HR [30], had little effect on the accumulation of GCRs in the *mms21-CH* mutant.

Class II mutations included deletions of *RAD59* and *CSM2*. Class II mutations partially suppressed the increased GCR rate caused by the *mm21-CH* mutation in the dGCR assay, but caused an increased GCR rate in the uGCR assay when combined with the *mms21-CH* mutation. Rad59 is a stimulatory factor for Rad52 and is important for HR involving shorter repeats or when Rad52 is absent [28]. Csm2 is a subunit of the Shu complex [31], which has been implicated as a regulator of HR, possibly by facilitating the formation of Rad51 filaments [28]; other Shu complex mutations were not tested. Consistent with these accessory roles in HR, deletions of *RAD59* and *CSM2* in the *mms21-CH* mutant modestly reduced the rate of accumulating GCRs in the dGCR assay (Fig 1, upper panel) and substantially increased the rate of accumulating GCRs in the uGCR assay in the *mms21-CH* mutant (Fig 1, lower panel).

Class III mutations included mutations in *MRE11* and *SAE2*. Class III mutations caused a modest increase in the increased dGCR rate caused by the *mms21-CH* mutation, but caused a substantial increase in the uGCR rate when combined with the *mms21-CH* mutation. The Mre11-Rad50-Xrs2 complex, together with Sae2, performs nucleolytic processing of DNA DSBs, leading to 5’-resection at DSBs and an ordered recruitment of HR proteins [28]. Deletion of *MRE11* has been shown to cause substantial increases in the rate of accumulation of GCRs [22], and deletion of *MRE11* in combination with the *mms21-CH* mutation caused an increase in the rate of accumulating GCRs in both the dGCR and uGCR assays compared to the *mms21-CH* single mutant (Fig 1). A mutation inactivating the endonuclease activity of Mre11, *mre11-H125N*, alone caused a 30-fold increase and a 122-fold increase in the rate of accumulating GCRs in the dGCR and uGCR assays, respectively (Fig 1). Interestingly, the *mre11-H125N* mutation did not appreciably affect the dGCR rate of the *mms21-CH* mutant, but caused a further increase in the uGCR rate of the *mms21-CH* mutant, suggesting the involvement of the Mre11 endonuclease activity in suppressing the GCRs selected in the uGCR assay. Sae2 participates in DNA DSB processing by specifically stimulating Mre11 endonuclease activity [32,33]. Like the *mre11-H125N* mutation, deletion of *SAE2* only modestly increased the dGCR rate of the *mms21-CH* mutant, but caused a much larger increase in the uGCR rate of the *mms21-CH* mutant. Thus, the initial nucleolytic processing by Mre11 endonuclease has a critical role in suppressing the formation of the GCRs selected in the uGCR assay in the *mms21-CH* mutant. In contrast, deletion of *EXO1*, which eliminates a key exonuclease that participates in long-range resection of DNA breaks, had little effect on the rate of accumulating GCRs selected in either the dGCR or uGCR assays in the *mms21-CH* mutant (Fig 1).

### Structures of GCRs formed in the wild-type strain and the *mre11* and *mms21-CH* mutant strains

**Fig 2:**
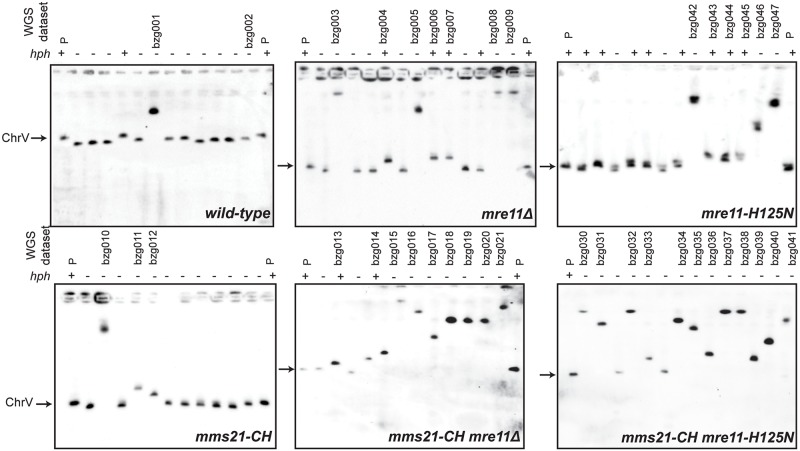
Analysis of the size of the rearranged chromosome V selected in wild-type, *mms21-CH*, *mre11Δ*, *mre11-H125N*, *mms21-CH mre11Δ*, and *mms21-CH mre11-H125N* uGCR strains. Chromosomes were separated by Pulsed-Field Gel Electrophoresis (PFGE) and Southern blotted using a probe for the essential chromosome V gene *MCM3*. The size of chromosome V in the parental strain (P) is indicated by an arrow. The retention (+) or loss (-) of the *hph* marker inserted next to the telomere on the left end of chromosome V is indicated above each lane in the gel. Isolates selected for whole genome sequencing are indicated with their WGS data set name above each lane.

To gain further insight into the effects of the loss of *MRE11* and *MMS21* function, we investigated the structures of the GCRs selected in the wild-type strain and the *mms21-CH*, *mre11Δ*, *mre11-H125N*, *mms21-CH mre11Δ*, and *mms21-CH mre11-H125N* mutant strains. We focused on GCRs selected in the uGCR assay, as the GCRs selected in the dGCR assay are almost exclusively duplication-mediated translocations formed by non-allelic HR between the *DSF1-HXT13* segmental duplication on chromosome V and regions of divergent homology on chromosomes IV, X and XIV, consistent with the HR gene dependency observed for GCRs selected in the dGCR assay in the *mms21-CH* mutant (S1 Fig). We first characterized the GCRs by testing the individual independent GCR-containing isolates for retention of the telomeric hygromycin resistance marker *hph* located between the telomere and the counter-selectable *CAN1/URA3* cassette on the uGCR assay chromosome and by determining the size of the rearranged chromosome V by Pulse Field Gel Electrophoresis (Fig 2; S2 Table). GCRs were divided into three groups: rearranged chromosomes that were larger than the wild-type chromosome V (group 1) and chromosomes that were similar to or slightly shorter than the wild-type chromosome V and either lost (group 2) or retained (group 3) the telomeric *hph* marker. We classified GCRs in group 2 as *de novo* telomere addition GCRs, which are formed by the healing of broken chromosomes by the *de novo* addition of a new telomere [34]. *De novo* telomere additions are the predominant form of GCRs selected in uGCR assays in strains without telomerase defects [29,35,36] and are always associated with loss of the *hph* marker [22], although it should be noted that rare interstitial deletion GCRs can be associated with deletion of the *hph* marker. Similarly, we classified GCRs in group 3 as interstitial deletion GCRs, in which the deletion is typically associated with non-homology or microhomology breakpoint junctions when selected in GCR assays containing only unique sequences in the breakpoint region like the uGCR assay used here [37].

**Fig 3:**
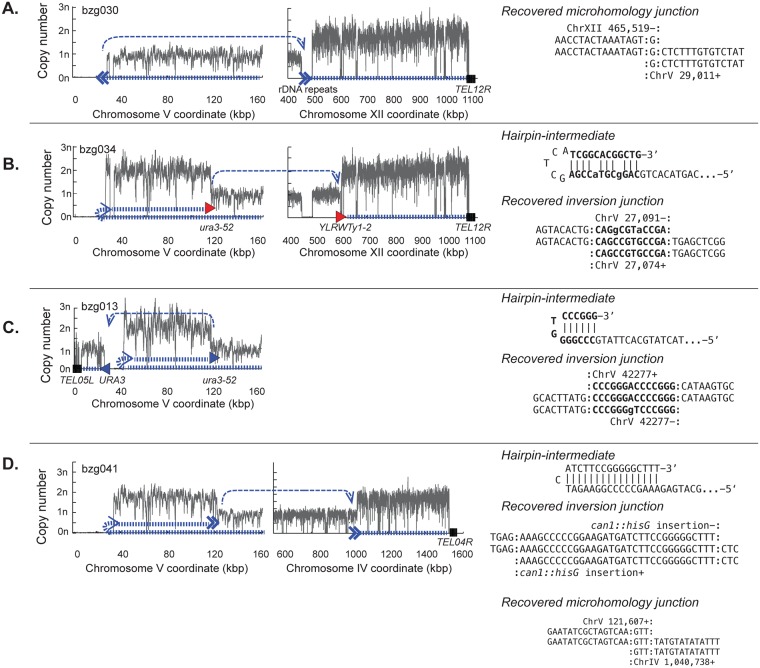
Types of GCRs identified by whole genome sequencing. Analysis of a microhomology-mediated translocation (**A**) and hairpin-mediated inverted duplications that were resolved by homologous recombination between Ty-derived elements (**B**), the homology between *URA3* and *ura3-52* (**C**), and a microhomology-mediated translocation (**D**). **Left**. Copy number analysis of uniquely mapping regions along a portion of the left arm of the assay-containing chromosome V derived from the read depth of uniquely mapping regions of the genome. **Middle**. Copy number analysis that, if present, shows other copy number changes elsewhere in the genome. **Left and Middle**. The path describing the GCR-containing chromosome is illustrated by the hashed thick blue line; the thin dashed blue lines indicate the connectivity between individual fragments that are separated on the reference genome. Homology-mediated translocation junctions are depicted with filled in triangles that point in the direction in which homology element points; junctions involving Ty-related homologies are red and other homologies are blue. Non-homology or micro-homology translocations are shown using two chevrons. Telomeres associated with the GCR (if known) are shown as a series of black vertical lines. **Right**. Sequences of any novel junctions are with the central line in the alignment corresponding to the novel junction. Sequences at the junction that could have been derived from either sequence are surrounded with colons. For GCRs with hairpin-mediated inversions, the inferred structure of the hairpin intermediate is also shown.

Strains containing GCRs falling into group 1 were subjected to whole genome paired end sequencing to determine the structures of the GCRs present (S3 Table). In addition to being able to detect all of the mutations and chromosome modifications introduced into the starting strains during strain construction (S2 and S3 Figs), we were also able to extensively characterize the structures of the GCR-containing chromosomes (Fig 3, S4–S9 Figs and S4 Table). We observed two distinct types of group 1 GCRs: *microhomology-mediated translocations* and *hairpin-mediated inverted duplications*.

In microhomology-mediated translocations, the broken end of a broken chromosome V is fused to another broken chromosome such that the broken chromosome V acquires a fragment of the second broken chromosome that is terminated with a telomere (Fig 3a). Copy number analysis indicated that these fusion events duplicated the non-chromosome V target, and junction sequences revealed only short sequences of identity at the translocation junctions. The copy number analysis was also consistent with the presence on an intact copy of the target chromosome, indicating that the microhomology-mediated translocations were non-reciprocal.

In hairpin-mediated inverted duplications, the broken end of a broken chromosome V is fused to an inverted copy of itself on the left arm of chromosome V at a position between the *CAN1/URA3* cassette and the first centromeric essential gene (Fig 3b, 3c and 3d). The inversion site sequences are consistent with a mechanism in which a broken chromosome V is resected to form a 3’ overhang that then pairs with a short stretch of homologous sequence centromeric to the breakpoint that is processed to yield a hairpin-terminated chromosome followed by replication of the hairpin-terminated chromosome (S10 and S11 Figs). As previously observed [36], these inverted dicentric duplication chromosomes (also called isoduplications) all underwent additional rounds of rearrangement that resolved them to the monocentric translocations observed, although other mechanisms for the hairpin formation and resolution are possible. These secondary rearrangements often, but not always, involved HR between the Ty- or *PAU* gene-related sequences on chromosome V L and a homology elsewhere in the genome (Fig 3b and 3c; S12–S14 Figs). The secondary rearrangements that initially appeared to involve HR between *ura3-52* on chromosome V and *YLRCdelta21* on chromosome XII actually proved to target an adjacent full-length Ty element on chromosome XII that was not present in the reference sequence (S15 Fig); this full-length Ty element has been previously observed by others [38,39]. A specific secondary rearrangement between a *URA3* fragment in the Ty-inactivated *ura3-52* on chromosome V L and the *URA3* in the *yel068c*::*CAN1/URA3* cassette first observed in GCRs derived from the *tel1Δ* uGCR strain was also observed here [36]. An additional type of secondary rearrangement was mediated by microhomologies (Fig 3d); microhomology-mediated secondary rearrangements were not observed in GCRs selected in *tel1Δ* mutants [36]. In most cases, the hairpin-mediated inverted duplications underwent a single secondary rearrangement as described above; however, in a small number of cases multiple rounds of secondary rearrangements were observed leading to the formation of monocentric GCRs (S4 Table).

### Distribution of GCRs formed in different mutant strains

**Fig 4:**
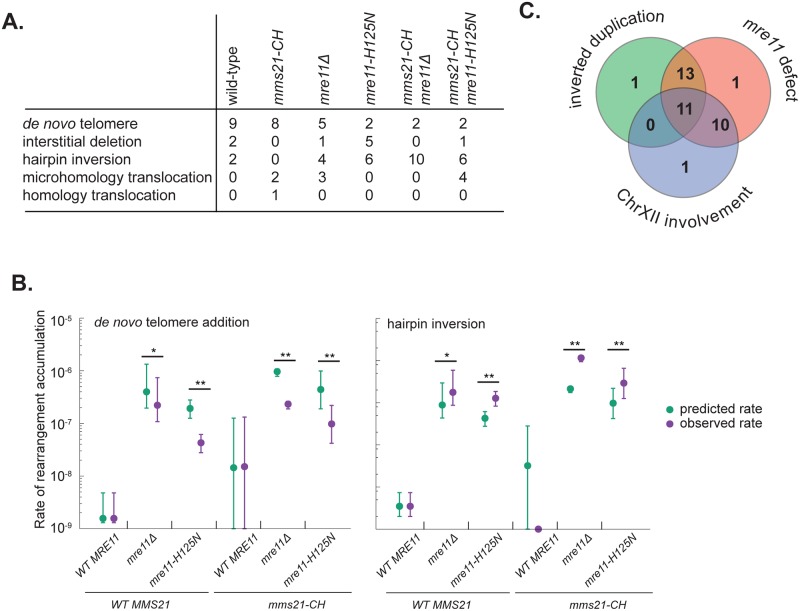
Distribution of GCR types from each mutant analyzed. **A**. Table of the number of each type of rearrangement, *de novo* telomere addition, interstitial deletion, hairpin-mediated inverted duplication, micro- and non-homology-mediated translocations, and homology-mediated translocations. **B**. The observed rates (purple) of accumulating *de novo* telomere addition GCRs and hairpin inversion GCRs were calculated by multiplying the observed rate and 95% confidence intervals by the fraction of GCRs in each mutant corresponding to each type of GCR. The expected rates (green) were calculated by multiplying the rates by the fraction of GCRs in the wild-type strain. Statistically significant differences between the observed and expected rates were calculated by the Mann-Whitney test; p-values < 0.05 displayed as ‘*’ and p-values < 0.0005 displayed as ‘**’. **C**. Venn diagram of sequenced GCRs demonstrates that strains with *MRE11* defects tend to accumulate GCRs involving inverted duplications and/or chromosome XII.

GCRs selected in the wild-type uGCR strain were primarily *de novo* telomere addition GCRs (Fig 4a; S7 Fig), consistent with dominance of *de novo* telomere addition GCRs among the GCRs selected in the “classical” GCR assay [35,40], which lacks large repetitive sequences in the breakpoint region like the uGCR assay used here. In addition, two interstitial deletions and two hairpin-mediated inverted duplications that were resolved by HR between the *ura3-52* allele and the *URA3* gene on the terminal chromosome V telomere-containing fragment were recovered. The spectrum of GCRs obtained from the *mms21-CH* uGCR strain shared this bias towards the formation of *de novo* telomere addition GCRs, with the other GCRs recovered being translocations involving other chromosomes (Fig 4a; S5 Fig). In contrast, substantially increased numbers of hairpin-mediated inverted duplications and decreased numbers of *de novo* telomere addition GCRs were selected in the *mre11Δ* and *mre11-H125N* single mutant uGCR strains and the *mms21-CH mre11Δ* and *mms21-CH mre11-H125N* double mutant uGCR strains (Fig 4a and 4b; S5–S9 Figs). These observations were consistent with role of the Mre11-Rad50-Xrs2 complex in cleaving hairpin structures [33] and the recovery of hairpin-mediated GCRs in strains containing an *mre11-H125N* mutation [41]. Remarkably, *MRE11*-deficient strains showed a bias for selection of translocations containing a copy of a long region of chromosome XII R (Fig 4c; S16 Fig), which could reflect either a bias due to increased fragility or accessibility of chromosome XII or due to suppression of *mre11*-dependent growth defects by duplication of chromosome XII R. We also observed that 8 of the 10 sequenced *mms21-CH mre11Δ* GCR-containing isolates were disomic for chromosome VIII and 1 of the 10 was disomic for chromosome I (S2 Table, S17 Fig). Taken together, these data are consistent with the idea that the *mms21-CH* mutation increases the total level of DNA damage without substantially biasing the mechanisms involved in forming GCRs, whereas *mre11* defects increase the propensity of damaged DNAs to form hairpin inversions.

### Roles of Pol32 and DNA helicases in the accumulation of GCRs in *mms21-CH* mutant strains

The dramatic HR-dependent increase in the dGCR rate caused by the *mms21-CH* mutation (Fig 1), combined with the fact that the *mms21-CH* mutation caused at best modest changes in the spectrum of GCRs selected in the uGCR assay (Fig 4 and S5 Table), suggested that the *mms21-CH* mutation causes an increase in DNA damage that underlies the formation of GCRs without dramatically affecting the DNA repair pathways that act on this DNA damage. Because HR appears to act on this DNA damage to produce GCRs selected in the dGCR assay, we investigated whether BIR or a BIR-related pathway might play a role in the formation of GCRs in *mms21-CH* dGCR strains.

**Fig 5:**
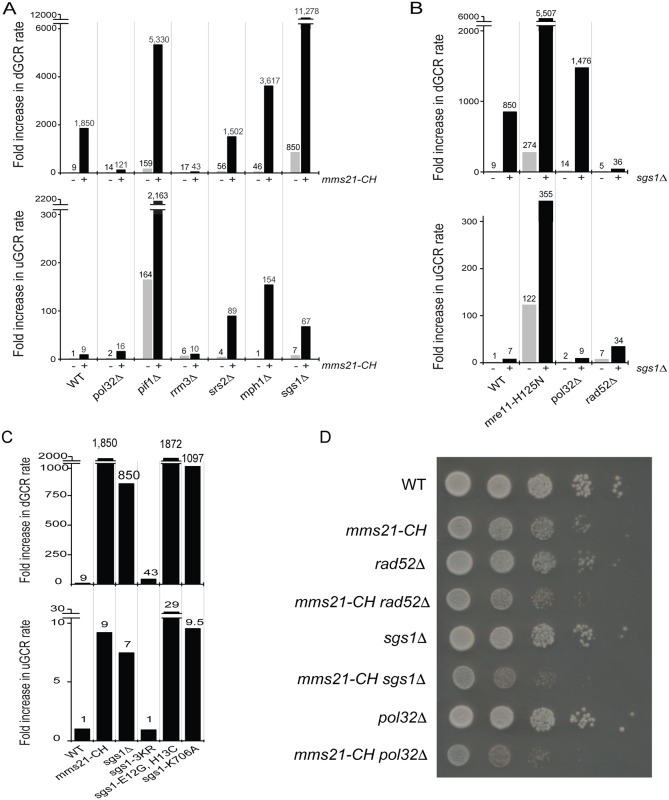
Role of Pol32 and DNA helicases in the formation of GCRs caused by *mms21-CH*. A) dGCR and uGCR rates caused by deleting *POL32*, *PIF1*, *RRM3*, *MPH1*, *SRS2* and *SGS1* in wild-type and *mms21-CH* mutant strains. B) Effect of combining *sgs1Δ* and *pol32Δ*, *rad52Δ or mre11-H125N* on the rate of accumulating GCRs. C) Effects of *sgs1-3KR* (K175R, K621R and K831R) mutations on the rate of accumulating GCRs. The number above each bar indicates the fold change normalized to the uGCR rate of wild-type strain. Detailed results used to generate the bar graph are shown in S5 Table. D) Combining *mms21-CH* with *rad52Δ*, *sgs1Δ* or *pol32Δ* causes a more severe growth defect in the double mutants. Equal amounts of cells, as measured by cell density, were 10-fold serially diluted, plated on YPD plate and incubated at 30 degree for 2 days before the picture was taken.

Previous studies of the repair of HO endonuclease-induced DNA DSBs by BIR showed that Pol32, a subunit of DNA polymerases delta and zeta, is required for BIR [42–44]. However, other studies have found that the *pol32Δ* mutation only reduced the efficiency of BIR [45]. We previously found that a *pol32Δ* mutation did not decrease the wild-type dGCR rate nor did the *pol32Δ* mutation eliminate duplication-mediated GCRs [22], suggesting that the role of *POL32* in promoting BIR may be dependent on the nature of the initiating damage or that the GCRs selected in the dGCR assay are not formed by BIR. Remarkably, we found that deletion of *POL32* in the *mms21-CH* mutant caused a drastic reduction of the dGCR rate by about 15-fold and a relatively modest increase in its uGCR rate (Fig 5A), consistent with an important role of *POL32*-dependent BIR in forming dGCRs in the *mms21-CH* mutant.

Pif1 has been shown to be required for BIR initiated by HO endonuclease-induced DSBs and is thought to act by promoting DNA synthesis mediated by a migrating D-loop replication intermediate [42,43]. Pif1 also dissociates telomerase from single-stranded DNA thereby suppressing GCRs mediated by *de novo* telomere addition at DSBs [29,46]. We found that deleting *PIF1* in the *mms21-CH* mutant caused further increases in both the dGCR and uGCR rates relative to that of the respective single mutants (Fig 5A), which is consistent with the idea that the DNA damage that underlies the formation of GCRs in the *mms21-CH* mutant is a substrate for *de novo* telomere additions [34,36].

We also screened other DNA helicases for their role in forming GCRs in *mms21-CH* mutant strains. *RRM3* encodes a DNA helicase that travels with DNA replication fork [47]. A recent study showed that Rrm3 participates in the repair of replication-associated DNA breaks [48], although it is not involved in BIR induced by HO endonuclease. Deletion of *RRM3* in the *mms21-CH* mutant caused a reduction (43-fold) in the dGCR rate without appreciably affecting the uGCR rate compared to that of the respective single mutants (Fig 5A), indicating a requirement of Rrm3 in the formation of duplication-mediated GCRs in the *mms21-CH* mutant strains. These findings suggest that accumulation of duplication-mediated GCRs in *mms21-CH* might reflect the formation of replication-associated DNA DSBs that require Rrm3 for BIR-like repair.

The DNA helicase Srs2 acts as an anti-recombinase by disrupting the formation of Rad51 filaments and D-loops [49–51]. In addition, the Smc5/6 complex of which Mms21 is a subunit has been shown to control the recombination activity of the Mph1 helicase [12,52]. Deletion of *SRS2* or *MPH1* in the *mms21-CH* mutant did not appreciably alter the dGCR rate, but caused a drastic increase in the uGCR rate relative to that of the respective single mutants (Fig 5A). This latter result could be explained if Srs2 and Mph1 either suppress the formation of a critical intermediate in the formation of the GCRs selected in the uGCR assay or target the initiating DNA damage to sister chromatid HR to an extent that suppresses GCRs selected in the uGCR assay, but not those selected in the dGCR assay.

The Sgs1 helicase has a major role in specifically suppressing dGCRs [16,22], and this has been attributed to its role in preventing crossovers during the resolution of HR intermediates [53]. Interestingly, combining an *sgs1Δ* with the *mms21-CH* mutation resulted in synergistic increases in both dGCR and uGCR rates relative to the respective single mutants (Fig 5A), indicating Mms21 and Sgs1 function in distinct pathways to prevent the formation of GCRs. To explore this further, we analyzed the effects of mutating *RAD52*, *MRE11* and *POL32* in the *sgs1Δ* mutant. A deletion of *RAD52* and the *mre11-H125N* mutation caused similar effects in *sgs1Δ* and *mms21-CH* mutants (comparing Figs 1 and 5). In contrast, deletion of *POL32* caused an increase in the dGCR rate of the *sgs1Δ* mutant whereas deletion of *POL32* in the *mms21-CH* mutant reduced the dGCR rate more than 10-fold (Fig 5B). Thus, the formation of duplication-mediated GCRs in the *mms21-CH* and *sgs1Δ* mutants had distinctly different requirements for Pol32. Because deletion of *RRM3* is lethal in an *sgs1Δ* mutant [54], we could not compare the role of Rrm3 in the *mms21-CH* and *sgs1Δ* mutants.

To determine whether the helicase activity or the Top3-binding activity of Sgs1 is involved in the suppressing of dGCRs, we analyzed *sgs1-K706A* (helicase-dead) and *sgs1-E12G*, *H13S* (Top3-binding defective) mutants [55]. We found that both *sgs1* mutations caused approximately the same increase in GCR rates compared to that caused by the *sgs1Δ* mutation, indicating that the function of Sgs1 in suppressing GCRs requires both its helicase activity and interaction with Top3-Rmi1 (Fig 5C). Recent studies showed that Mms21 specifically catalyzes sumoylation of Sgs1 in response to treatment with DNA alkylating agents [10,20,21]. We found that the *sgs1-3KR* mutation that eliminates the sumoylation sites on Sgs1 did not cause a comparable increase in GCR rates to that seen in the *sgs1Δ* mutant (Fig 5C). Although we cannot exclude the possibility that a low and undetectable level of Sgs1 sumoylation occurs in the *sgs1-3KR* mutant, this result indicates that the major DNA damage-induced sumoylation of Sgs1 does not have an appreciable role in preventing spontaneous GCRs.

Because the DNA lesions accumulated in the *mms21-CH* mutant are repaired by a variety of DNA repair pathways, especially the HR and more specifically BIR pathway, we reason that inactivating these DNA repair pathways in the *mms21-CH* mutant could cause a synergistic growth defect. Consistent with this view, we found that mutations of *rad52Δ*, *sgs1Δ* and *pol32Δ* caused a significant slower growth when they are combined with the *mms21-CH* mutation (Fig 5D). Thus, a failure in properly repairing the spontaneous DNA lesions accumulated in the *mms21-CH* mutant is detrimental to cell growth.

### The *mms21-CH* mutation induces spontaneous DNA lesions and activates the DNA damage checkpoint to promote dGCRs

**Fig 6:**
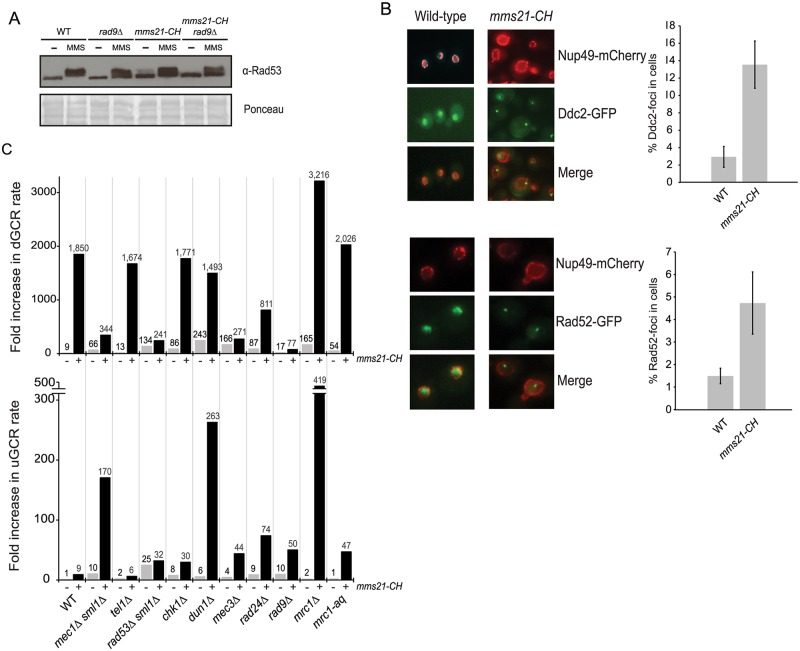
Role of DNA damage checkpoint in the formation of GCRs. A) Rad53 gel shift assay to examine Rad53 activation in WT, *mms21-CH*, *rad9Δ* and *rad9Δ mms21-CH* mutants. B) Spontaneous Ddc2 foci in *mms21-CH* mutant and WT, which also contain Nup49-mCherry to mark the nuclear envelope. Bar graph indicates the percentage of Ddc2-foci within the nuclear envelope (marked by Nup49-mCherry). Error bars represent the standard deviation from three replicate experiments using two biological isolates per strain per replicate. 200–400 cells were imaged and counted for each experiment. C) dGCR and uGCR rates caused by mutations of DNA damage checkpoint genes with or without *mms21-CH*. The number above each bar indicates the fold change normalized to the uGCR rate of wild-type strain. Detailed results used to generate the bar graph are shown in S6 Table.

The above findings are consistent with the idea that the *mms21-CH* mutation causes accumulation of spontaneous DNA lesions that underlie the formation of a diverse range of GCRs. Cells have evolved a signal transduction pathway, the DNA damage checkpoint, to detect endogenous DNA lesions [56]. We therefore examined the Rad53 kinase, which becomes hyperphosphorylated in the presence of such DNA damage and migrates with a slower electrophoretic mobility than non-phosphorylated Rad53. Treatment by the DNA alkylating agent methyl methane sulfonate (MMS) caused a pronounced electrophoretic mobility shift of fully activated Rad53 to slower migrating species (Fig 6A). An increased but sub-stoichiometric amount of Rad53 was found to show slower gel mobility in an *mms21-CH* mutant that was not treated with MMS compared to untreated wild-type, indicating that Rad53 is partially activated in *mms21-CH* mutants. Deletion of *RAD9*, which encodes an adaptor protein that acts to promote DNA damage-induced activation of Rad53, reduced the amount of the slower migrating species of Rad53 to an undetectable level in the *mms21-CH* mutant that was not treated with MMS; note that MMS-induced Rad53 phosphorylation still occurs in a *rad9Δ* mutant due to the redundant role of Mrc1 in mediating Rad53 activation [57–59]. Ddc2, together with the Mec1 kinase, is recruited to RPA-coated single stranded DNA at the sites of DNA damage where it can be visualized as sub-nuclear foci [60]. A higher incidence of Ddc2 foci was seen in the untreated *mms21-CH* mutant compared to untreated wild-type cells (Fig 6B). We also examined the localization of Rad52, which forms foci at the sites of DNA lesions that undergo DNA repair by HR [60]. Similar to Ddc2 foci, an elevated incidence of Rad52 foci was detected in the *mms21-CH* mutant compared to wild-type cells (Fig 6B). Together, these results suggest that elevated levels of endogenous DNA lesions occur in the *mms21-CH* mutant that activate the DNA damage checkpoint and are and processed by Rad52-mediated HR.

We next asked whether the DNA damage checkpoint influences the formation of GCRs in the *mms21-CH* mutant (Fig 6C and S6 Table). The DNA damage checkpoint involves two partially redundant protein kinases, Mec1 and Tel1. While Mec1 has a major role in controlling the DNA damage response, Tel1 has an important role in telomere length maintenance in wild-type cells and in checkpoint responses in *mec1* mutants [61,62]. We found that deletion of *MEC1* caused a 5-fold reduction in the dGCR rate of the *mms21-CH* mutant (Fig 6C, upper panel) and a substantial increase in the uGCR rate of the *mms21-CH* mutant (Fig 6C, lower panel). Unlike the *mec1Δ* mutation, deletion of *TEL1* caused little of no change in the dGCR and uGCR rates of the *mms21-CH* mutant. Like the deletion of *MEC1*, deletions of the *MEC3*, *RAD24* and *RAD9* genes involved in the DNA damage checkpoint, caused varying degrees of reduction of the dGCR rate of the *mms21-CH* mutant with the *rad9Δ* mutation causing the greatest reduction (~ 24-fold). In contrast, deletions of the *MEC3*, *RAD24* and *RAD9* genes in the *mms21-CH* mutant resulted in increases in the uGCR rate, consistent with the known role of DNA damage checkpoint in suppressing GCRs mediated by single-copy sequences [63].

Unlike deletion of *RAD9*, deletion of *MRC1* caused a modest (2-fold) increase in the dGCR rate of the *mms21-CH* mutant (Fig 6C, upper panel). Interestingly, deletion of *MRC1* caused a drastic increase in the uGCR rate of the *mms21-CH* mutant (Fig 6C, lower panel). Because Mrc1 also has a role in DNA replication [64], we next examined the *mrc1-AQ* mutant, all of whose Mec1 consensus phosphorylation sites are mutated to non-phosphorylatable alanines and is thus unable to mediate Rad53 activation [65]. We found that the *mrc1-aq* mutation did not appreciably alter the dGCR rate of the *mms21-CH* mutant although it did cause an increase in the uGCR rate of the *mms21-CH* mutant, but not to the extent seen with the *mrc1Δ* mutation. Rad53, Chk1, and Dun1 are the downstream effector kinases of the checkpoint pathways. Deletion of *RAD53* reduced the dGCR rate of the *mms21-CH* mutant by about 9-fold (Fig 6C, upper panel), while deletion of *CHK1* did not appreciably alter the dGCR rate of the *mms21-CH* mutant and caused a small increase in the uGCR rate of the *mms21-CH* mutant. Although deletion of *DUN1* did not appreciably alter the dGCR rate of the *mms21-CH* mutant, it caused a synergistic increase in the uGCR rate of the *mms21-CH* mutant (Fig 6C, lower panel). These data support the idea that the Mec3/Rad24-Rad9-Mec1-Rad53 pathway plays a role in promoting the GCRs selected in the dGCR assay in the *mms21-CH* mutant while suppressing the GCRs selected in the uGCR assay in the *mms21-CH* mutant.

Because the *mms21-CH* mutation appears to cause increased levels of some type of DNA damage, we tested the affect of the *mms21-CH* mutation on HR between *ade2* heteroalleles in a mitotically growing diploid strain [66]. The rate of accumulating Ade+ recombinants in the wild-type strain was 5.0 x10^-5^ [95% confidence interval = 1.8 x10-5–8.5 x10^-5^] and the rate in the *mms21-CH* mutant was 3.2 x10^-5^ [95% confidence interval = 2.0 x10-5–1.0 x10^-4^] indicating that there was no change in the rate of HR between *ade2* heteroalleles in the *mms21-CH* mutant. This is unlike the effect of the *mms21-CH* mutation on the formation of GCRs. General DNA damage such as γ-rays is known to induce HR between heteroalleles [67]. A possible explanation for the difference between the effect of *mms21-CH* on heteroallelic HR and the formation of GCRs is that the DNA damage that occurs in the *mms21-CH* mutant is only subjected to one-ended HR such as BIR. This damage, most likely a DSB, would rarely result in HR between heteroalleles as the damage would need to occur between the heteroalleles to yield recombinants, and this is a much smaller target than the region in which a DSB would yield a GCR [22].

## Discussion

**Fig 7:**
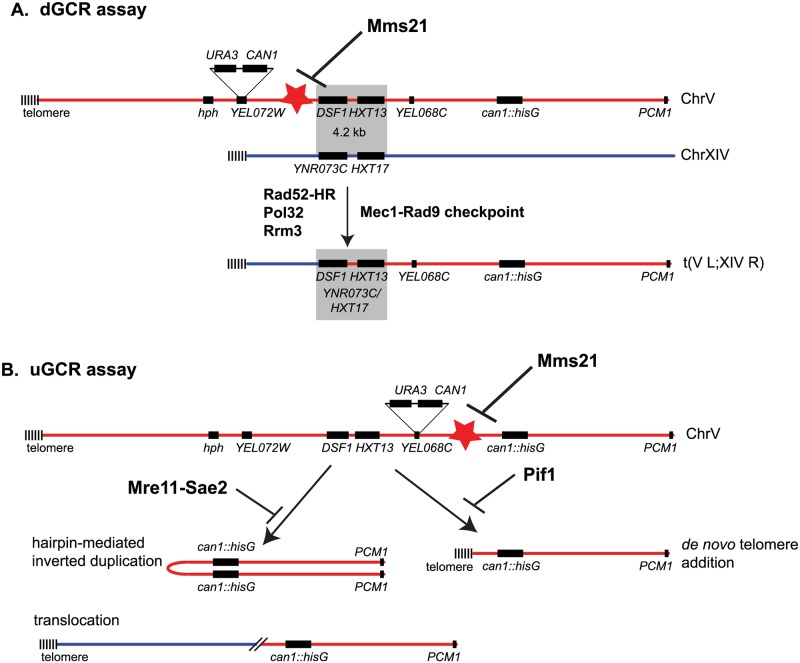
Summary of the functions of the Mms21 E3 ligase pathway in preventing genome rearrangements. A) dGCR assay detects BIR-like events, driven by Pol32, Rrm3, DNA damage checkpoint and Rad52 dependent non-allelic HR. B) uGCR assay detects micro-homology mediated translocations and hairpin mediated inverted duplications prevented by the Mre11-Sae2 endonuclease, and *de novo* telomere addition prevented by the Pif1 helicase. These GCR events are stimulated by inactivating Mms21 E3 ligase to cause DNA lesions, depicted as red stars.

Mutations affecting the Mms21 SUMO E3 ligase cause substantially increased accumulation of DNA damage intermediates and increased accumulation of GCRs [4,5,10]. Here we show that the Mms21 E3 ligase plays an important role in suppressing the formation of GCRs selected in the dGCR assay, which are typically translocations mediated by non-allelic HR (Fig 7). The duplication-mediated GCRs formed in Mms21 E3 ligase-null mutants appear to be formed by *POL32*-dependent BIR-related event in contrast to those formed in wild-type strains, which have little dependence on Pol32 [22]. The increased rate of accumulating GCRs selected in the uGCR assay caused by *mms21-CH* mutations, which does not reflect the formation of duplication-mediated GCRs, is not accompanied by a change in the spectrum of GCRs relative to the spectrum of GCRs selected in the uGCR assay in wild-type strains. In addition, the *mms21-CH* mutation causes increased accumulation of spontaneous Ddc2 and Rad52 foci. These observations suggest that the *mms21-CH* mutation causes increased levels of DNA damage that trigger the DNA damage checkpoint and are processed into GCRs. We cannot rule out the possibility that Mms21 plays roles in some DNA repair pathways; however, the accumulated evidence presented here suggests that Mms21 suppresses genome instability primarily by preventing the formation of initiating DNA damage that potentially occurs during DNA replication.

The GCRs selected in the dGCR assay are primarily translocations formed by non-allelic HR between the *DSF1-HXT13* region on chromosome V and divergent homologous sequences elsewhere in the genome [22]. The genetic requirements for the formation of the duplication-mediated GCRs that occur at increased rates in *mms21-CH* mutants are consistent with the idea that these GCRs are formed by non-allelic HR mediated by Pol32-dependent BIR; the increased dGCR rates were greatly reduced when the genes required for HR were deleted, were partially reduced when accessory HR genes were deleted and were greatly reduced when the *POL32* gene required for BIR initiated by HO endonuclease-induced DSBs was deleted [44]. The fact that the *pol32Δ* mutation does not decrease the dGCR rate in wild-type cells [22] may suggest that the DNA lesions that initiate the formation of duplication-mediated GCRs in *mms21-CH* mutants are more similar to HO endonuclease-induced DSBs or are readily converted to such DSBs than the DNA lesions that underlie duplication-mediated GCRs in wild-type cells. This possibility is also consistent with the constitutive activation of the DNA damage checkpoint in *mms21-CH* mutants as indicated by increased Rad53 hyperphosphorylation and increased levels of Ddc2 and Rad52 foci in *mms21-CH* mutants (Fig 6). The Pif1 DNA helicase has also been shown to be required for BIR initiated from HO endonuclease-induced DSBs [42,43]; however, a *pif1Δ* mutation did not decrease the dGCR rate of *mms21-CH* mutants, although this predicted effect of the *pif1Δ* mutation on BIR could be masked by the large increase in the rate of *de novo* telomere addition GCRs that occurs in *pif1Δ* strains [34,37]. Moreover, the increase in the *mms21-CH* rates in the uGCR and dGCR assays caused by a *pif1Δ* mutation is consistent with the possibility that *MMS21* suppresses the formation of DNA damage as loss of the Pif1 DNA helicase causes increased GCR rates when combined with many different mutations that lead to increased levels of DNA damage. Together, these data argue that the increased dGCR rate seen in *mms21-CH* mutants is the result of increased non-allelic HR that is most likely mediated by BIR due to increased levels of DNA lesions that are substrates for BIR. We have not ruled out the possibility that the *mms21-CH* mutation also alters the activity of some of the HR proteins.

We have found that duplication-mediated GCRs that occur at increased rates in *mms21-CH* mutants depend on both the Rrm3 DNA helicase and the DNA damage checkpoint. Unlike Pif1, Rrm3 is not known to be required for HO-induced DSB-mediated BIR, and purified Rrm3 is not able to replace Pif1 in the extension of D-loops by DNA polymerase delta *in vitro* [43]. However, Rrm3, a homolog of Pif1 [68], might also play a role in promoting the formation of *mms21-CH*-induced GCRs mediated by BIR similar to how Pif1 acts [42,43]. Recently, it was reported that Rrm3 has an important role in repairing DNA DSBs originating from damaged DNA replication forks [48]. This finding and the requirement of Rrm3 in mediating the formation of duplication-mediated GCRs in *mms21-CH* mutant reported in this study raises the possibility that the spontaneous DNA legions that accumulate in *mms21-CH* mutants likely result from defective DNA replication forks that are processed into DNA DSBs. This possibility is also consistent with the synergistic increase in GCR rates caused by combining *mms21-CH* with *mrc1* mutations that cause DNA replication defects. The resulting DNA DSBs are substrates for Pol32-dependent BIR that also involves Rrm3; in contrast, Pif1 may play a more important role than Rrm3 in repairing HO endonuclease-induced DNA DSBs by similar BIR [42,43]. We have also identified the DNA damage checkpoint as promoting the formation of duplication-mediated GCRs in *mms21-CH* mutants. The DNA damage checkpoint has well-documented roles in promoting the homology search during HR [69] which could promote the production of the GCRs selected in the dGCR assay. The DNA damage checkpoint could also act to delay the cell cycle to allow the dGCR events being recovered in our assay. Remarkably, deletion of *RAD9* caused a specific reduction of the dGCR rate, which could suggest that GCR-initiating DNA lesions associated with active or stalled replication forks are converted to DSBs that are recognized by the Rad9 branch of the DNA damage checkpoint to facilitate homology search and/or allow time for HR repair.

Loss of either Mms21/NSMCE2 or Sgs1/BLM causes increased levels of aberrant HR intermediates, SCE and GCRs [4,5,10,11,14], and sumoylation of Sgs1 by Mms21 prevents the formation of aberrant HR intermediates in response to alkylation damage [20,21]. Despite these similarities, the increase in the dGCR and uGCR rates caused by combining the *mms21-CH* and *sgs1Δ* mutations argues strongly that these proteins act in different pathways that suppress the formation of GCRs. Consistent with this conclusion, deletion of *POL32* had differing effects on dGCR rates in *mms21-CH* strains compared to deleting *SGS1*. Sgs1 is important in resolving HR intermediates [17]; hence, the GCR-based genetic interactions between *sgs1Δ* and *mms21-CH* mutations seen here suggest that Mms21 prevents the formation of damage that underlies aberrant HR and that Sgs1 acts to edit these aberrant HR intermediates to prevent non-allelic HR. In this regard, our analysis indicated that it is the DNA helicase activity of Sgs1 that functions in conjunction with Rmi1 and Top3 to suppress GCRs rather than some function of Sgs1 mediated by sumoylation. Thus, despite the known role of Mms21-dependent sumoylation of Sgs1 in repairing exogenously induced DNA damage [20,21], the function of Mms21 in preventing spontaneous genome rearrangements is distinct from that of Sgs1.

The increased accumulation of DSBs or damage that can be converted to DSBs in *mms21-CH* mutant strains is also consistent with the structures of the GCRs selected in the uGCR assay as determined by whole genome sequencing. The *mms21-CH* single mutants have increased rates of accumulating *de novo* telomere addition GCRs and microhomology-mediated translocation GCRs, which reflect different mechanisms of healing broken chromosomes. Interestingly, mutations affecting *MRE11* caused an increase in hairpin-mediated inverted duplication GCRs as well as decrease in *de novo* telomere addition GCRs alone and when combined with an *mms21-CH* mutation, which is consistent with previous observations [35,41]. These results are consistent with increased formation of DSBs in an *mms21-CH* mutant combined with the inability of *mre11* mutants to cleave DNA hairpins generated from these DSBs [33]. The fact that the relative increase in hairpin-mediated GCRs occurs with a relative decrease in *de novo* telomere addition GCRs in *mre11* mutants suggests that hairpin formation is likely to be faster at broken chromosomes than the addition of a new telomere. Due to the relatively small numbers of events classified, there is insufficient evidence to suggest that *mre11Δ* and *mre11-H125N* differ significantly in the microhomology-mediated translocations or hairpin-mediated inverted duplications; instead, these events are likely caused by the same defect in nucleolytic processing of DNA lesions in these *mre11* mutants.

Together, the findings presented here argue that mutations inactivating the Mms21 E3 ligase lead to an accumulation of DNA lesions that are either DNA DSBs or are easily convertible to DNA DSBs, and that these DSBs lead to diverse genome rearrangements, depending on the available DNA repair pathways (Fig 7). These spontaneous DNA breaks likely result from damaged or defective DNA replication forks and would occur in chromosomal regions that have yet to be fully replicated. In this case, the resulting DSBs would be one-ended which would force these DSBs to be repaired by various DNA repair pathways including BIR with either the sister chromatid or homologous sites elsewhere in the genome. The differences between the *mms21-CH* and wild-type strains, for example in the *POL32*-dependence of duplication-mediated GCRs, suggest that much of the damage giving rise to GCRs in wild-type cells may be more complicated than simple DSBs. Considering Mms21 has been shown to sumoylate proteins with essential roles in DNA replication, including the MCM2-7 replicative helicase [4,6], it is tempting to speculate that defects in Mms21-mediated sumoylation of the DNA replisome could cause defective DNA replication, leading to accumulation of DNA DSBs that drive the formation of GCRs. If so, the study of the role of Mms21-dependent sumoylation in regulating DNA replication could provide important insights into how protein sumoylation may prevent genome rearrangements.

## Materials and methods

### *S*. *cerevisiae* strain construction and genetic methods

Standard *S*. *cerevisiae* genetics method was used to introduce mutations. *S*. *cerevisiae* strains used in this study are listed in S7 Table. Generation of the *sgs1-3KR* mutation involved two steps by first deleting the N-terminal region of Sgs1 from -425bp to 2600bp and then repairing it using PCR products containing the *sgs1-3KR (K175R*, *K621R and K831R)* mutations with a *HIS3* marker located at 435bp upstream of the starting codon of Sgs1 to preserve its native promoter. DNA sequencing was used to confirm the integration of *sgs1* mutations. Methods used for fluctuation analysis to determine GCR rates have been described previously [70]. Briefly, 14–16 rate measurements were performed using 7–8 independent cultures derived from 2 independently isolated strains and the GCR rate was determined by the method of the median. Fluctuation analysis was also used to determine the rate of HR between *ade2* heteroalleles. Diploids were generated from wild-type and *mms21-CH* derivatives of previously published haploid strains containing different *ade2* alleles [66], one due to an insertion of the I-SceI cut site (*ade2-I*) and another due to a 2bp deletion at the *NdeI* site (*ade2-n*). The I-SceI endonuclease was not expressed, allowing spontaneous HR to be measured. Cells were grown from single colonies overnight in YPD until cultures reach an OD_600_ of 0.5 and plated onto CSM complete or CSM minus adenine medium and grown for 3–4 days before counting the resulting colonies.

### Microscopy

Cells were grown in CSM medium to log phase and examined by live imaging using Olympus BX43 fluorescence microscope with a 60x, 1.42 PlanApo N Olympus Oil immersion objective. GFP and mCherry fluorescence were detected using a Chroma FITC filter set and a TxRed filter set respectively and captured with a Qimaging QIClick CCD camera. Images were captured using Meta Morph Advanced 7.7 imaging software. Figures were prepared in Adobe Photoshop, keeping processing parameters constant within each experiment.

### PFGE and Southern blotting

DNA plugs for PFGE were prepared as described [71]. Electrophoresis was performed using a Bio-Rad CHEF-DRII apparatus at 6 V/cm, with a 60 to 120 s switch time for 25 h. The gels were stained with ethidium bromide and imaged. The DNA in the gel was transferred to Hybond-XL membranes by neutral capillary blotting. The DNA was crosslinked to the membrane by UV irradiation in a Stratalinker (Stratagene) apparatus at maximum output for 60 seconds. The *MCM3* probe was generated by amplifying *MCM3* from genomic DNA using the primers 5’-CTGTGCAAGAAATGCCCGAAATG-3’ and 5’-GCCCCGGAGTTGGAATGCTC-3’ followed by random primer labeling of the PCR product with the Biotin DecaLabel DNA Labeling Kit (Thermo Scientific). Probe hybridization was performed at 50°C for 1 hr. Biotin signal was detected using Chemiluminescent Nucleic Acid Detection Module Kit (Thermo Scientific).

### Whole genome paired-end sequencing

Multiplexed paired-end libraries were constructed from 2 μg of genomic DNA purified using the Purgene kit (Qiagen). The genomic DNA was sheared using M220 focused-ultrasonicator (Covaris) and end-repaired using the End-it DNA End-repair kit (Epicentre Technologies). Common adaptors from the Multiplexing Sample Preparation Oligo Kit (Illumina) were then ligated to the genomic DNA fragments, and the fragments were then subjected to 18 cycles of amplification using the Library Amplification Readymix (KAPA Biosystems). The amplified products were fractionated on an agarose gel to select 600 bp fragments, which were subsequently sequenced on an Illumina HiSeq 4000 using the Illumina GAII sequencing procedure for paired-end short read sequencing. Reads from each read pair were mapped separately by bowtie version 2.2.1 [72] to a reference sequence that contained revision 64 of the *S*. *cerevisiae* S288c genome (http://www.yeastgenome.org), *hisG* from *Samonella enterica*, and the *hphMX4* marker. Sequence data is available from National Center for Biotechnology Information Sequence Read Archive under accession number: SRP106876.

### Rearrangement and copy number analysis of paired-end sequencing data

Chromosomal rearrangements were identified after bowtie mapping by version 0.6 of the Pyrus suite (http://www.sourceforge.net/p/pyrus-seq) [36]. Briefly, after removal of PCR duplicates, read pairs in which both reads uniquely mapped were used to generate the read depth and span depth copy number distributions. The read depth copy number distribution is the number of times each base pair was read in a sample; read depth distributions were the distributions plotted to examine copy number (S4–S9 Figs) as this distribution is less distorted than the span depth distribution in regions adjacent to repetitive elements. The span depth copy number distribution is the number of times each base pair in a sample was contained in a read or spanned by a pair of reads; span depth distributions were used to statistically distinguish real rearrangements identified by junction-defining discordant read pairs from discordant read pairs that were noise in the data. Read pair data were then analyzed for junction-defining discordant read pairs that indicated the presence of structural rearrangements relative to the reference genome. Identified rearrangements included junctions produced during strain construction, such as the *his3Δ200* deletion (see S2 and S3 Figs), or GCR-related rearrangements (see S4–S9 Figs). Associated junction-sequencing reads, which were reads that did not map to the reference but were in read pairs in which one end was adjacent to discordant reads defining a junction, were used to sequence novel junctions. Most hairpin-generated junctions (S11 Fig) could be determined using alignments of junction-sequencing reads. For junctions formed by HR between short repetitive elements (S12–14 Figs) and for problematic hairpin-generated junctions (S11 Fig), the junction sequence could be derived by alignment of all reads in read pairs where one read was present in an “anchor” region adjacent to the junction of interest and the other read fell within the junction to be sequenced. Similar strategies involving the alignment of reads paired with reads present in “anchor” regions also were used to sequence *de novo* telomere addition junctions (S4–S9 Figs) and to identify the “*YLRWTy1-4*” Ty element that was not present in the reference genome (S15 Fig).

### Rad53 gel shift analysis

Protein extracts for Western blot analysis was prepared using a TCA (trichloroacetic acid) extraction. To examine Rad53 electrophoretic mobility we used an anti-Rad53 monoclonal antibody (EL7E1 serum) from mouse, a gift from Dr. Marco Foiani.

## Supporting information

S1 Fig The dGCR and uGCR assays identify chromosomal rearrangements through the selection of canavanine (Canr) and 5-fluoroorotic acid (5FOA) resistant cells. Assays were constructed by placing a *CAN1/URA3* cassette telomeric to *PCM1*, the most telomeric essential gene, into a strain with a deletion of *CAN1*, the *ura3-52* allele, and a telomeric hygromycin resistance marker (*hph*). **A**. The uGCR (*yel068c*::*CAN1/URA3*) assay predominantly generates GCRs mediated by *de novo* telomere additions; interstitisal deletions, hairpin-mediated inverted duplications, and translocations are also observed. **B**. The dGCR (*yel072w*::*CAN1/URA3*) assay predominantly generates GCRs by HR using repeated homologies (grey box) on chromosomes IV, X, and XIV. Other GCRs, like those observed in the uGCR assay, can also form.
(PDF)

S2 Fig Identification of the starting chromosomal features on chromosome V by whole-genome sequencing. For each junction along chromosome V (junctions 5-A to 5-J), the evidence for each junction in the paired-end sequencing data is reported. The number preceding the slash is the number of junction-defining read pairs (those for which one read maps to one side of the junction and the other read maps to the other side of the junction). The number following the slash is the number of junction-sequencing reads (those that can be aligned to derive the sequence of the junction). “-/-”indicates a junction that could have been observed but was not, which is typically due to a GCR-related deletion. “n.a.” indicates a junction that could not have been observed as it was not present in the parental strain, such as the *mms21-CH*.*kanMX6* junctions in *MMS21* strains. Note that some sequences are short enough that some read pairs span multiple junctions, e.g. junction 5-IJ contains read pairs that span both junctions 5-I and 5-J.
(PDF)

S3 Fig Identification of the starting chromosomal features on chromosomes other than chromosome V by whole-genome sequencing. Evidence for each junction is displayed as for S1 Fig.
(PDF)

S4 Fig Analysis of GCRs selected in the uGCR assay in a wild-type strain. Copy number analysis of uniquely mapping regions using read depth from the whole genome sequencing data reveals the presence of deletions and duplications associated with the formation of GCRs. Read depth was scaled by the median read depth of concordant read pairs to determine 1n copy number. Graphs on the left indicate the copy number distribution along a portion of the left arm of the assay-containing chromosome V. For each isolate, the region of ChrV containing the *yel068c*::*CAN1/URA3* cassette is deleted. Graphs in the center, if present, show other copy number changes elsewhere in the genome. In relevant cases, the sequences of any novel junctions are shown in the sequence alignments on the right. For the sequence alignment, the central line is the novel junction. The lines above and below are the alignments to the two regions in the genome. Regions between the two colons represent identical sequences at the junction that could have been derived from either sequence. More complex junctions, such as hairpin-mediated inversions, are shown in S10–S12 Figs. The path describing the GCR-containing chromosome is illustrated by the thick hashed blue line; the thin dashed blue lines indicate the connectivity between individual fragments that are separated on the reference genome. For a description of these summarized junctions, see S6 Table. Homology-mediated translocations are depicted with filled in triangles that point in the direction in which homology element points; junctions involving Ty-related homologies are red and other homologies are blue. Non-homology or micro-homology translocations are shown using two chevrons. Telomeres associated with the GCR (if known) are shown by the black box.
(PDF)

S5 Fig Analysis of GCRs selected in the uGCR assay in an *mms21-CH* single-mutant strain. Data are displayed as in S4 Fig.
(PDF)

S6 Fig Analysis of GCRs selected in the uGCR assay in an *mre11Δ* single-mutant strain. Data are displayed as in S4 Fig.
(PDF)

S7 Fig Analysis of GCRs selected in the uGCR assay in an *mre11-H12N* single-mutant strain. Data are displayed as in S4 Fig.
(PDF)

S8 Fig Analysis of GCRs selected in the uGCR assay in an *mms21-CH mre11Δ* double-mutant strain. Data are displayed as in S4 Fig.
(PDF)

S9 Fig Analysis of GCRs selected in the uGCR assay in an *mms21-CH mre11-H125N* double-mutant strain. Data are displayed as in S4 Fig.
(PDF)

S10 Fig General mechanism for the formation of hairpin-mediated inverted duplications. A mechanism that can explain the formation of the inverted duplication junctions observed here (A and S11 Fig). A. An example of an inversion junction sequence identified in *mms21-CH mre11Δ* GCR isolate bzg013 is displayed with the inversion junction in center and the sequence alignments to chromosome V in opposite orientation above and below. B. The inversion junction can be formed by 5’ resection from a double-stranded break (DSB) to generate a 3’-overhang. Intramolecular loop formation mediated by intra-strand base pairing generates a 3’ primer terminus that can be extended by DNA polymerases. This initial hairpin-capped chromosome will generate a dicentric chromosome upon replication, which is unstable and undergoes additional rounds of rearrangement. Note that if a nuclease like Rad1-Rad10 is present to cleave flap-containing DNA molecules [73], then the double-strand break need to not occur at the end of the homology, but rather an annealed primer terminus can be generated by flap cleavage, analogously to the cleavage of 3’ non-homologous tails during homologous recombination [74].
(PDF)

S11 Fig Structure of the key hairpin-intermediate in hairpin-mediated inverted duplications inferred from the inversion junction sequences. **A-Q**. For most panels, the mapped junction sequence is displayed on top and the key hairpin-containing intermediate (see S10 Fig) is displayed on bottom. **C**. The inversion-containing sequence was derived by aligning all reads that paired with reads mapping to chrV:34,339–34,739 and pointed in a forward orientation (e.g. paired with a read in the chrV:34,339–34,739 region that pointed in the reverse orientation). This was necessary as the inversion site occurred next to the *can1*::*hisG* junction within a distance shorter than the median intra-read pair distance. **H**. The middle sequence is the engineered junction between *CAN1* and *YEL068C* that introduces plasmid sequence not present in the reference *S*. *cerevisiae* genome, but is involved in the formation of the resected intermediate during hairpin formation for the *mms21-CH mre11Δ* GCR bzg015. I. The middle sequence is the engineered junction between *hisG* and the *CAN1* flanking region in the *can1*::*hisG* locus that is not present in the reference *S*. *cerevisiae* genome, but is involved in hairpin formation for the *mms21-CH mre11Δ* GCR bzg016. **O**. For the *mms21-CH mre11-H125N* bzg034, a complete sequence of the inversion was not obtained; however, reads adjacent to the inversion could be aligned to generate two almost identical sequences, each of which defined one half of the inversion (see arrows at bottom). **Q**. The inversion-containing sequence was derived by aligning all reads that paired with reads mapping to chrV:34,339–34,739 and pointed in a forward orientation (e.g. paired with a read in the chrV:34,339–34,739 region that pointed in the reverse orientation). This was necessary as the inversion site occurred next to the *can1*::*hisG* junction within a distance shorter than the median intra-read pair distance. Arrows indicate the two copies of the inverted sequence.
(PDF)

S12 Fig Sequence of the *YERWdelta6/YLRWdelta6* junction in GCR bzg017. Sequence of the junction between *YELWdelta6* (yellow) and *YLRWdelta6* (red) that fuses the inverted duplication on chromosome V (magenta) with chromosome XII (grey). Sequence that could have been derived from either *YELWdelta6* or *YLRWdelta6* is displayed with an orange background.
(PDF)

S13 Fig Sequence of the *PAU2/PAU20* junction in GCR bzg021. Sequence of the junction between *PAU2* (yellow) and *PAU20* (red) that fuses the inverted duplication on chromosome V (magenta) with chromosome XII (grey). Sequences that could have been derived from either *PAU2* or *PAU20* are displayed with an orange background.
(PDF)

S14 Fig Sequence of the *YERWdelta6/ura3-52* junction in GCR bzg014. Sequence of the junction between *YELWdelta6* (yellow) and *ura3-52* (red). Sequence that could have been derived from either *YELWdelta6* or *ura3-52* is displayed with an orange background.
(PDF)

S15 Fig Evidence for a full-length Ty element adjacent to *YLRCdelta12* on chromosome XII. **A**. Analysis of copy number changes for GCRs bzg016, bzg018, and bzg042 using the S288c reference genome indicates that the duplication on chromosome XII begins adjacent to *YLRCdelta21*, which is in the opposite orientation relative to *ura3-52* required to generate the breakpoint junction by HR. **B**. To understand the nature of the target region, read pairs with one read mapping to either side of *YLRCdelta21* were analyzed in a sequenced uGCR strain that lacked a chromosome XII rearrangement (RDKY6761, [36]). First, read pairs in which one read mapped to Anchor region A (chrXII:817,711–818,470) preceding *YLRCdelta21* were collected, and all reads that pointed in the reverse direction (e.g. reads that paired with a read that mapped in the forward direction in Anchor region A) were aligned. This alignment was identical to chrXII:817,711–818,470; however, the end of alignment did not map to chromosome XII adjacent to this region, but rather mapped to many delta sequences throughout the genome. Second, Anchor region B (chrXII:818,426–818,800) following *YLRCdelta21* was analyzed using alignments from all reads pointing in the forward direction. This generated two alignments. The first alignment was identical to chrXII:818,470–818,849 but was preceded by a sequence containing the 3’ end of Ty sequence. The second alignment, derived from reads mapping to chrXII:818,387–818,467 were identical to the reference chromosome. Taken together, these junction sequences are consistent with the insertion of a full-length Ty in the forward orientation; called here “*YLRWTy1-4*” as the sequence of this element is most similar to other Ty1 elements in the S288c reference genome. The presence of this forward-oriented Ty element resolves the orientation problem for the formation of the chromosome V:XII translocation in GCRs bzg016, bzg018, and bzg042, and this Ty element has been previously observed in other strains derived in the S288c background [38,39]. **C**. A partial sequence of the junctions between chromosome XII and *YLRWTy1-4*. Note that the insertion of *YLRWTy1-4*, like other Ty1 elements [75], is also associated with the duplication of 5 bp on either side of the element.
(PDF)

S16 Fig Duplication of regions of chromosome XII R in GCRs selected in *MRE11*-deficient strains. **A**. Strains with mutations affecting *MRE11* show a propensity to duplicate regions of the right arm of chromosome XII. Duplicated regions from each sequenced isolate are drawn as horizontal lines with solid red triangles indicating Ty homology-mediated translocations and double blue chevrons indicating micro- or non-homology translocations. The centromere is depicted as a black circle, the rDNA repeats as a black box, and the telomeres as a series of vertical lines. Genes on chromosome XII R that are involved in DNA repair (yellow), telomere homeostasis (green), or genetically interact with *MRE11* (grey) are depicted above the chromosomal region where they are encoded. **B**. Genetic interactions reported in BioGRID [76] between *MRE11* and genes (color coded as above) encoded in the duplicated regions of chromosome XII R. Black lines indicate growth defects (synthetic lethality, synthetic growth defect, negative genetic E-MAP measurements), and red lines indicate phenotypic rescue or phenotypic enhancement.
(PDF)

S17 Fig Evidence for disomy of chromosome VIII and chromosome I in some of the GCR-containing *mms21-CH mre11Δ* strains. Copy number histograms for all sixteen chromosomes in the sequenced *mms21-CH mre11Δ* GCR-containing isolates are shown. Duplicated regions have twice the read depth as non-duplicated regions. Chromosomes duplicated by GCR-related events show a bimodal distribution. Chromosomes that are disomic such as chromosome VIII (bzg013, bzg014, bzg016, bzg018, bzg019, bzg020, bzg021, and bzg022) and chromosome I (bzg017) show a single peak at twice the read depth as most other chromosomes. No other disomic chromosomes were observed in these strains and no disomies were observed in any other strain analyzed. Note that the common chromosome VIII disomy cannot be attributed to the parental strain, as bzg013 and bzg015 to bzg022 were derived from one parental strain and bzg014 was derived from another parental strain.
(PDF)

S1 Table Fluctuation results of double mutants containing a null mutation of genes in the Rad52 pathway and *mms21-CH*. (PDF)

S2 Table Summary of Can^R^ 5FOA^R^ isolates from the uGCR assay. (DOCX)

S3 Table Statistics for whole genome sequencing results. (DOCX)

S4 Table Number of junction-defining read pairs and junction-sequencing reads for Can^R^ 5FOA^R^ isolates from the uGCR assay with sequenced genomes. (PDF)

S5 Table Fluctuation results of double mutants containing a null mutation of genes in the BIR pathway and *mms21-CH* or *sgs1Δ*. (PDF)

S6 Table Fluctuation results of double mutants containing a null mutation of genes in the DNA damage checkpoint pathways and *mms21-CH*. (PDF)

S7 Table *S*. *cerevisiae* strains used in this study. (DOCX)
