# Supplementary material for: SUMO E3 ligase Mms21 prevents spontaneous DNA damage induced genome rearrangements
Source: PLoS Genet. 2018 Mar 5;14(3):e1007250. doi: 10.1371/journal.pgen.1007250 (PMC5860785; doi:10.1371/journal.pgen.1007250)
Supplement: S1 Fig — Assays were constructed by placing a CAN1/URA3 cassette telomeric to PCM1, the most telomeric essential gene, into a strain with a deletion of CAN1, the ura3-52 allele, and a telomeric hygromycin resistance marker (hph). A. The uGCR (yel068c::CAN1/URA3) assay predominantly generates GCRs mediated by de novo telomere additions; interstitisal deletions, hairpin-mediated inverted duplications, and translocations are also observed. B. The dGCR (yel072w::CAN1/URA3) assay predominantly generates GCRs by HR using repeated homologies (grey box) on chromosomes IV, X, and XIV. Other GCRs, like those observed in the uGCR assay, can also form. (PDF) [file pgen.1007250.s001.pdf]

S1 Figure

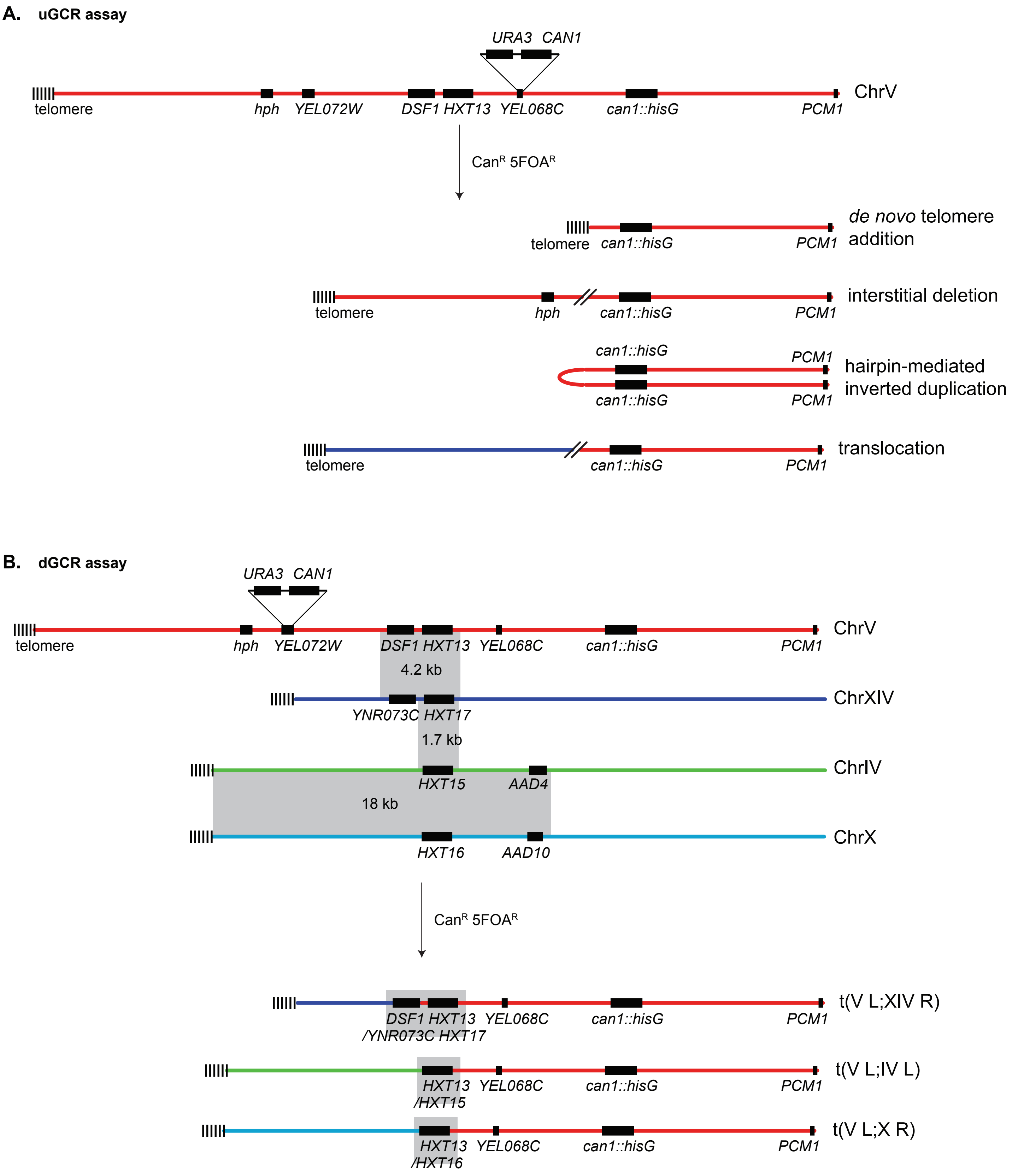

+ de novo telomere addition, interstitial deletions, hairpin-mediated inverted duplications, translocations
