# Supplementary figures and images for: SUMO E3 ligase Mms21 prevents spontaneous DNA damage induced genome rearrangements
Source: PLoS Genet. 2018 Mar 5;14(3):e1007250. doi: 10.1371/journal.pgen.1007250 (PMC5860785; doi:10.1371/journal.pgen.1007250)

S2 Figure

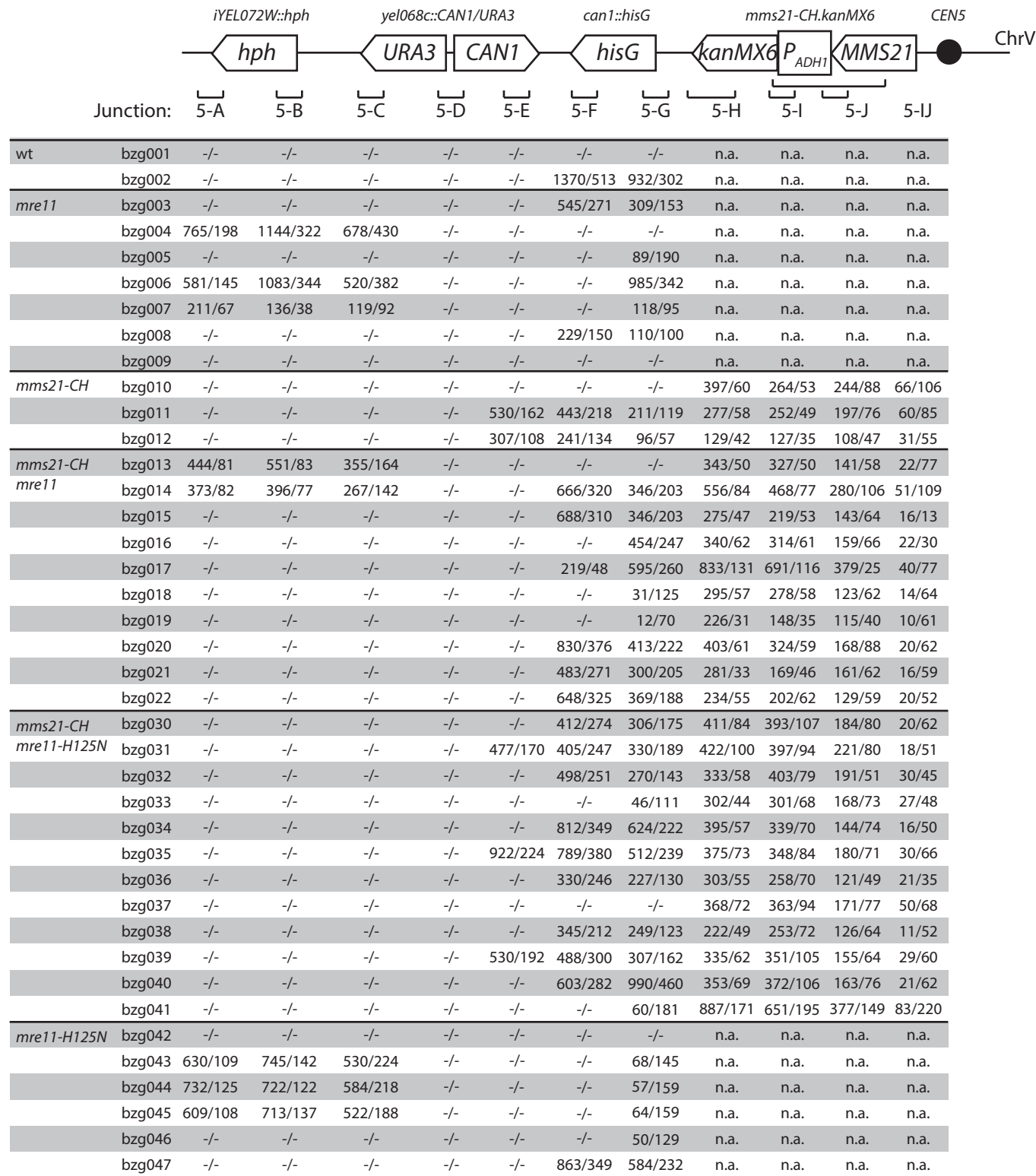

Supplement: S2 Fig — For each junction along chromosome V (junctions 5-A to 5-J), the evidence for each junction in the paired-end sequencing data is reported. The number preceding the slash is the number of junction-defining read pairs (those for which one read maps to one side of the junction and the other read maps to the other side of the junction). The number following the slash is the number of junction-sequencing reads (those that can be aligned to derive the sequence of the junction). “-/-”indicates a junction that could have been observed but was not, which is typically due to a GCR-related deletion. “n.a.” indicates a junction that could not have been observed as it was not present in the parental strain, such as the mms21-CH.kanMX6 junctions in MMS21 strains. Note that some sequences are short enough that some read pairs span multiple junctions, e.g. junction 5-IJ contains read pairs that span both junctions 5-I and 5-J. (PDF) [file pgen.1007250.s002.pdf]

S4 Figure

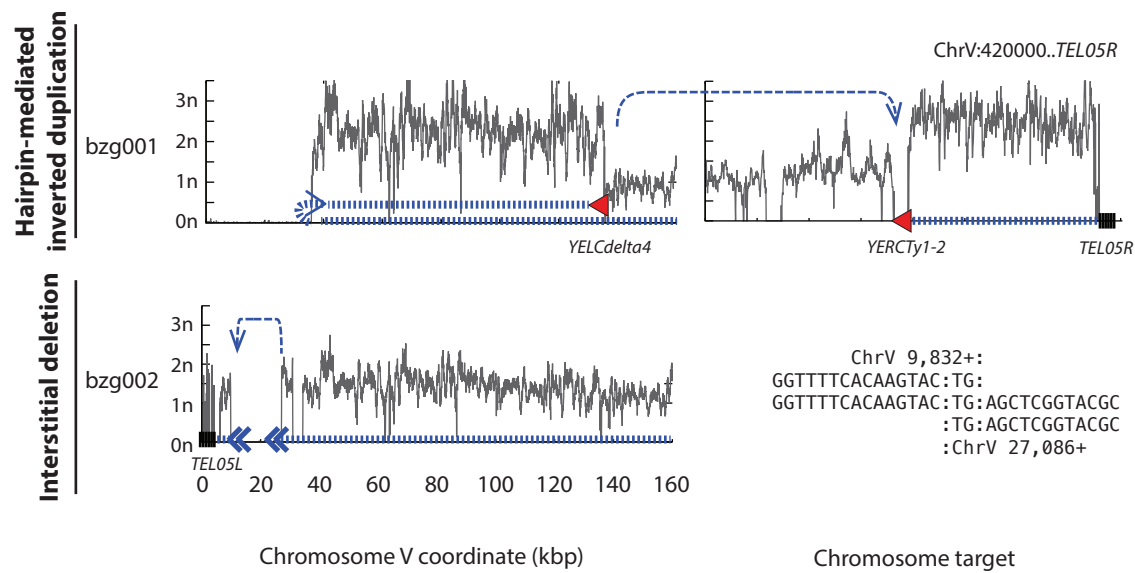

Supplement: S4 Fig — Copy number analysis of uniquely mapping regions using read depth from the whole genome sequencing data reveals the presence of deletions and duplications associated with the formation of GCRs. Read depth was scaled by the median read depth of concordant read pairs to determine 1n copy number. Graphs on the left indicate the copy number distribution along a portion of the left arm of the assay-containing chromosome V. For each isolate, the region of ChrV containing the yel068c::CAN1/URA3 cassette is deleted. Graphs in the center, if present, show other copy number changes elsewhere in the genome. In relevant cases, the sequences of any novel junctions are shown in the sequence alignments on the right. For the sequence alignment, the central line is the novel junction. The lines above and below are the alignments to the two regions in the genome. Regions between the two colons represent identical sequences at the junction that could have been derived from either sequence. More complex junctions, such as hairpin-mediated inversions, are shown in S10–S12 Figs. The path describing the GCR-containing chromosome is illustrated by the thick hashed blue line; the thin dashed blue lines indicate the connectivity between individual fragments that are separated on the reference genome. For a description of these summarized junctions, see S6 Table. Homology-mediated translocations are depicted with filled in triangles that point in the direction in which homology element points; junctions involving Ty-related homologies are red and other homologies are blue. Non-homology or micro-homology translocations are shown using two chevrons. Telomeres associated with the GCR (if known) are shown by the black box. (PDF) [file pgen.1007250.s004.pdf]

S5 Figure

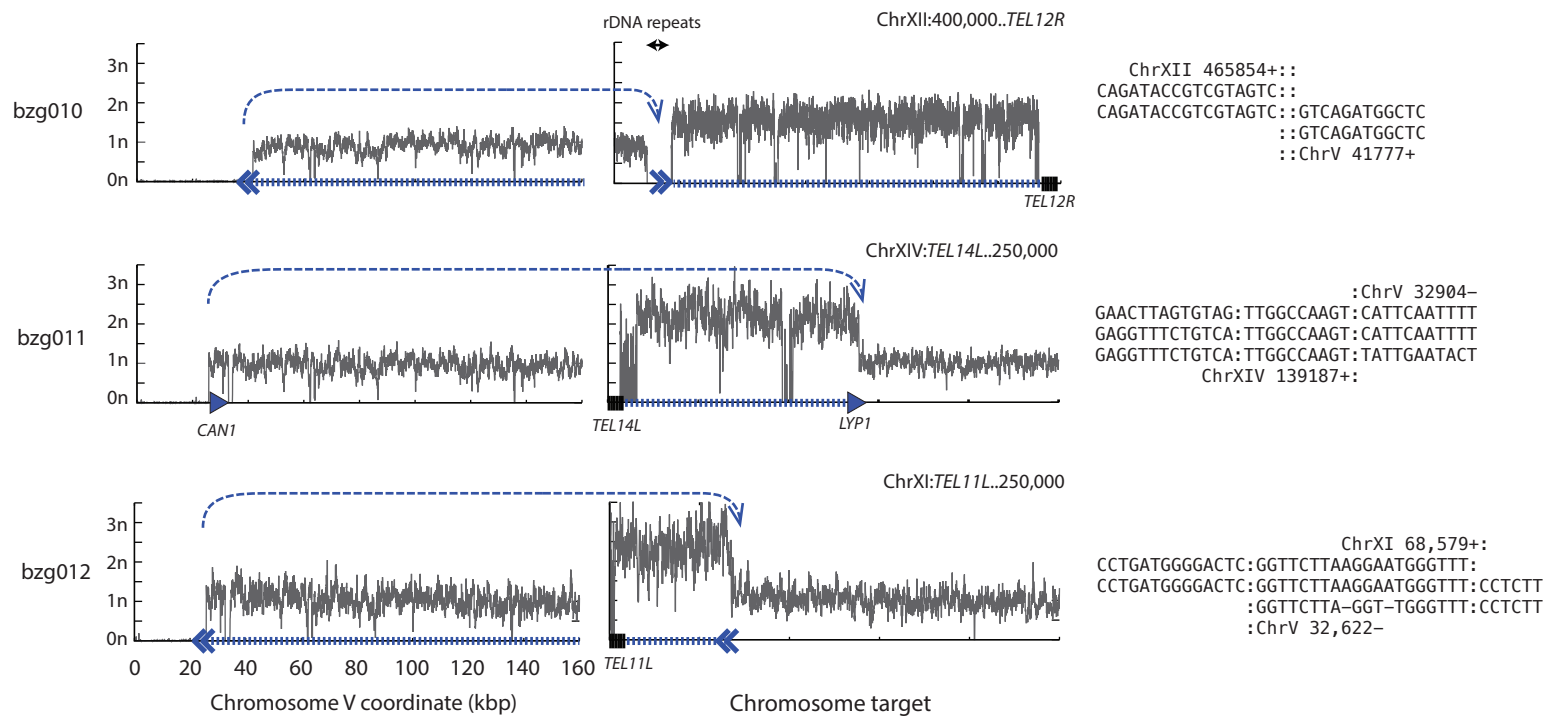

Supplement: S5 Fig — Data are displayed as in S4 Fig. (PDF) [file pgen.1007250.s005.pdf]

S6 Figure

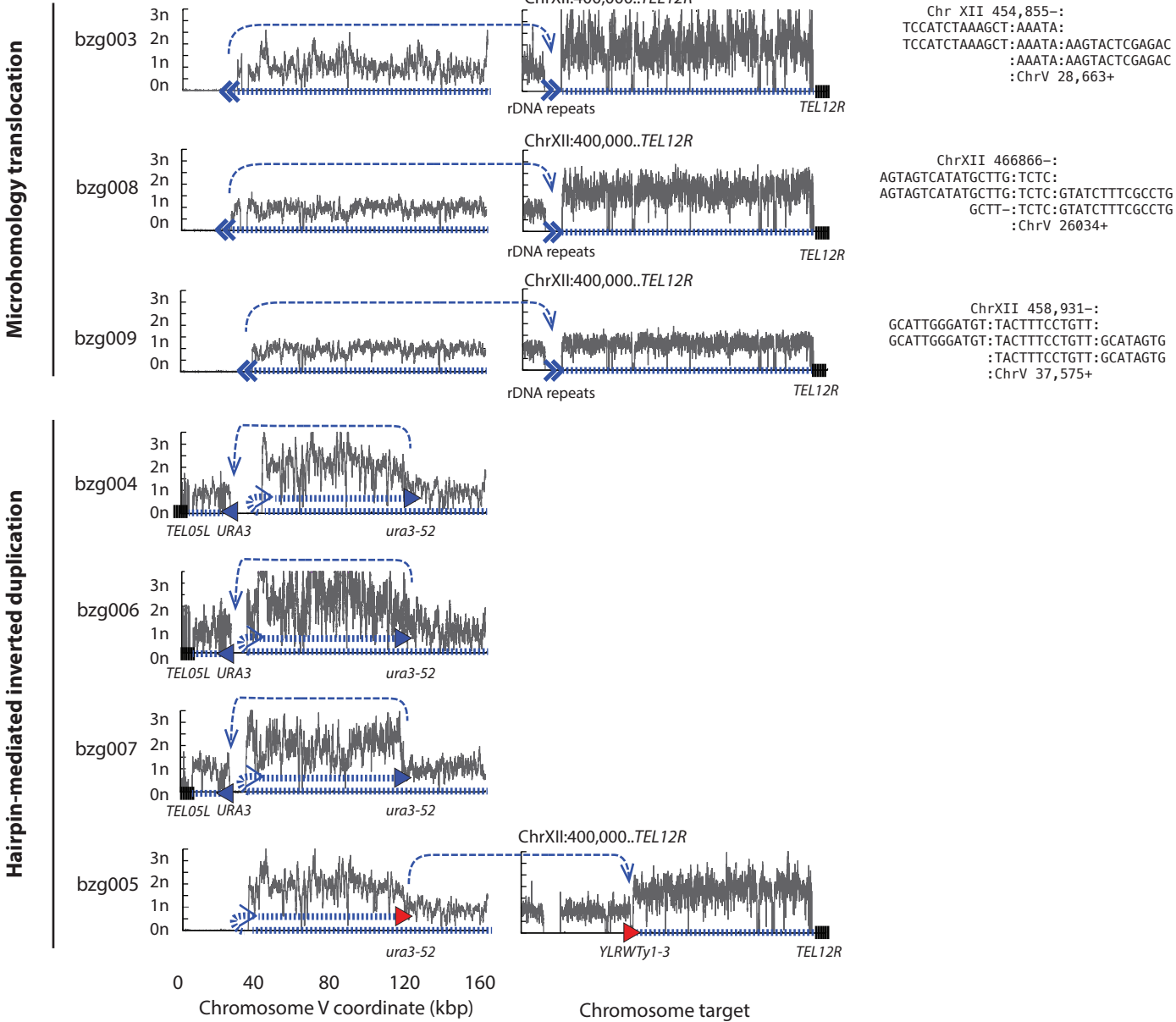

Supplement: S6 Fig — Data are displayed as in S4 Fig. (PDF) [file pgen.1007250.s006.pdf]

S7 Figure

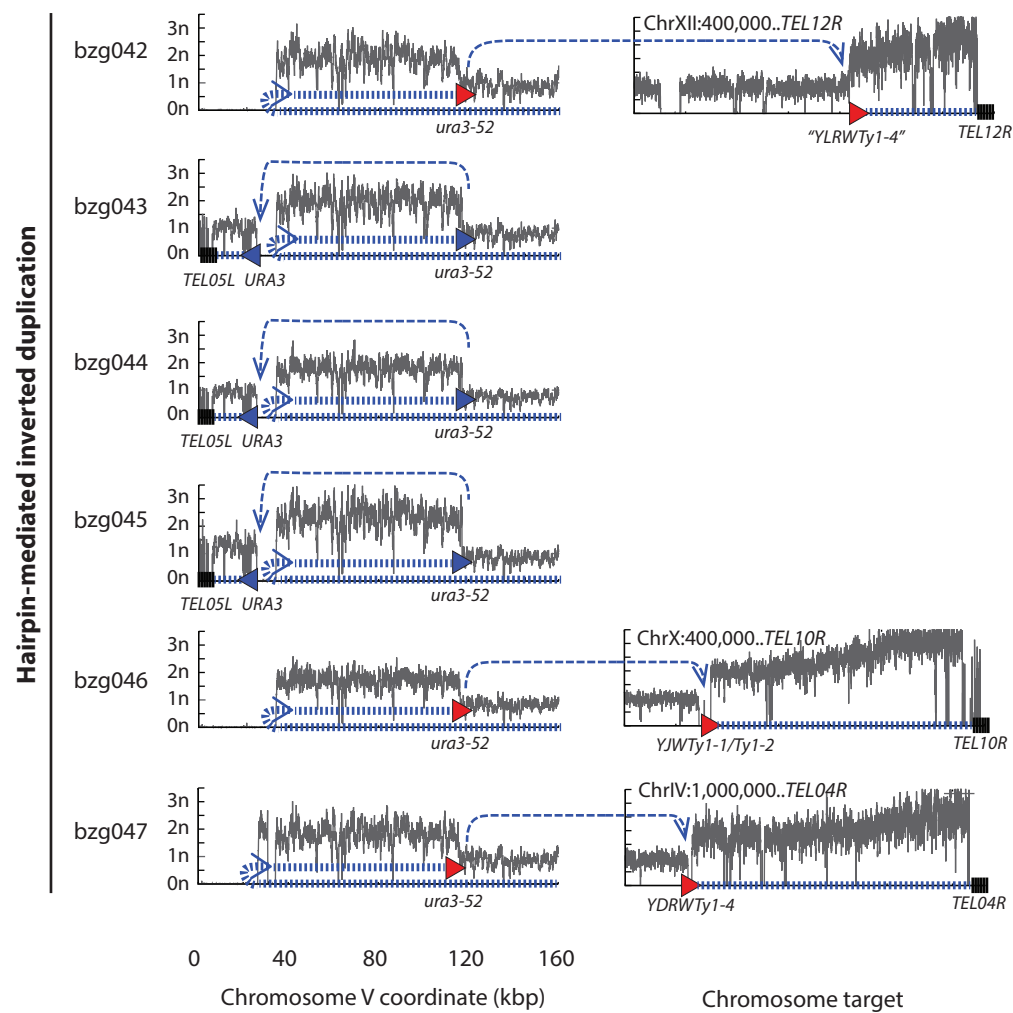

Supplement: S7 Fig — Data are displayed as in S4 Fig. (PDF) [file pgen.1007250.s007.pdf]

S8 Figure

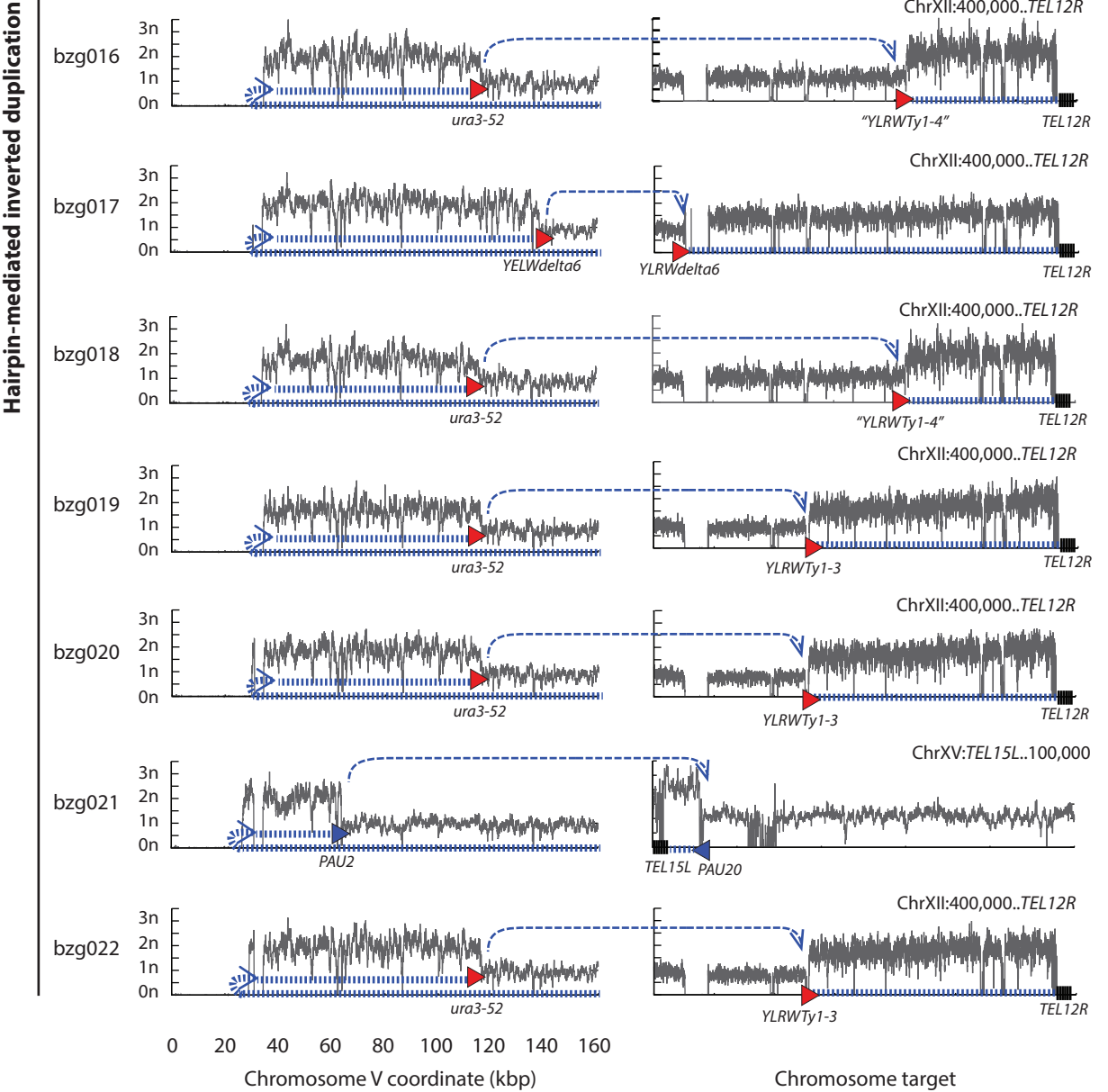

Supplement: S8 Fig — Data are displayed as in S4 Fig. (PDF) [file pgen.1007250.s008.pdf]

S9 Figure

Microhomology/non-homology translocation

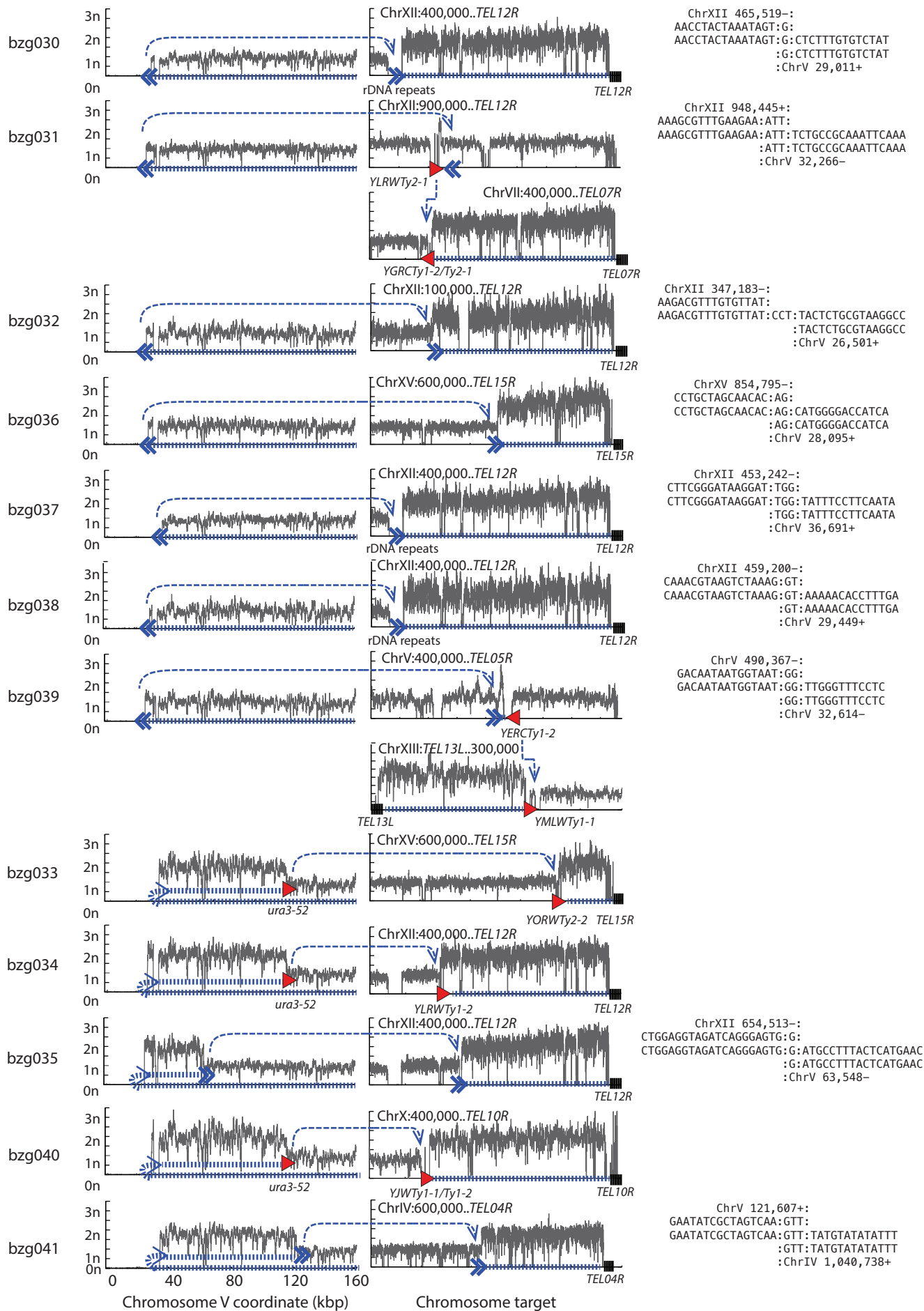

Supplement: S9 Fig — Data are displayed as in S4 Fig. (PDF) [file pgen.1007250.s009.pdf]

S13 Figure

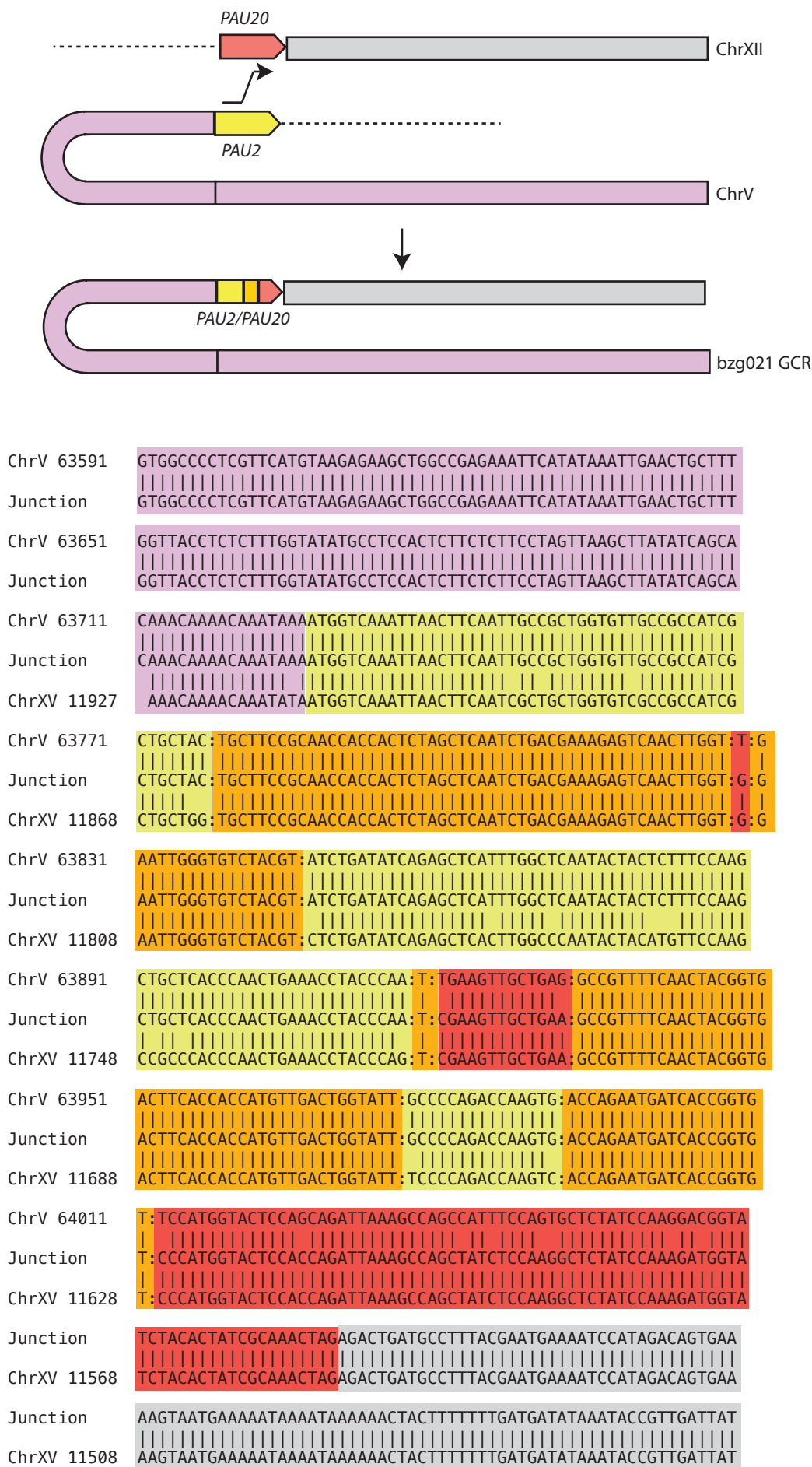

Supplement: S13 Fig — Sequence of the junction between PAU2 (yellow) and PAU20 (red) that fuses the inverted duplication on chromosome V (magenta) with chromosome XII (grey). Sequences that could have been derived from either PAU2 or PAU20 are displayed with an orange background. (PDF) [file pgen.1007250.s013.pdf]

S16 Figure

A.

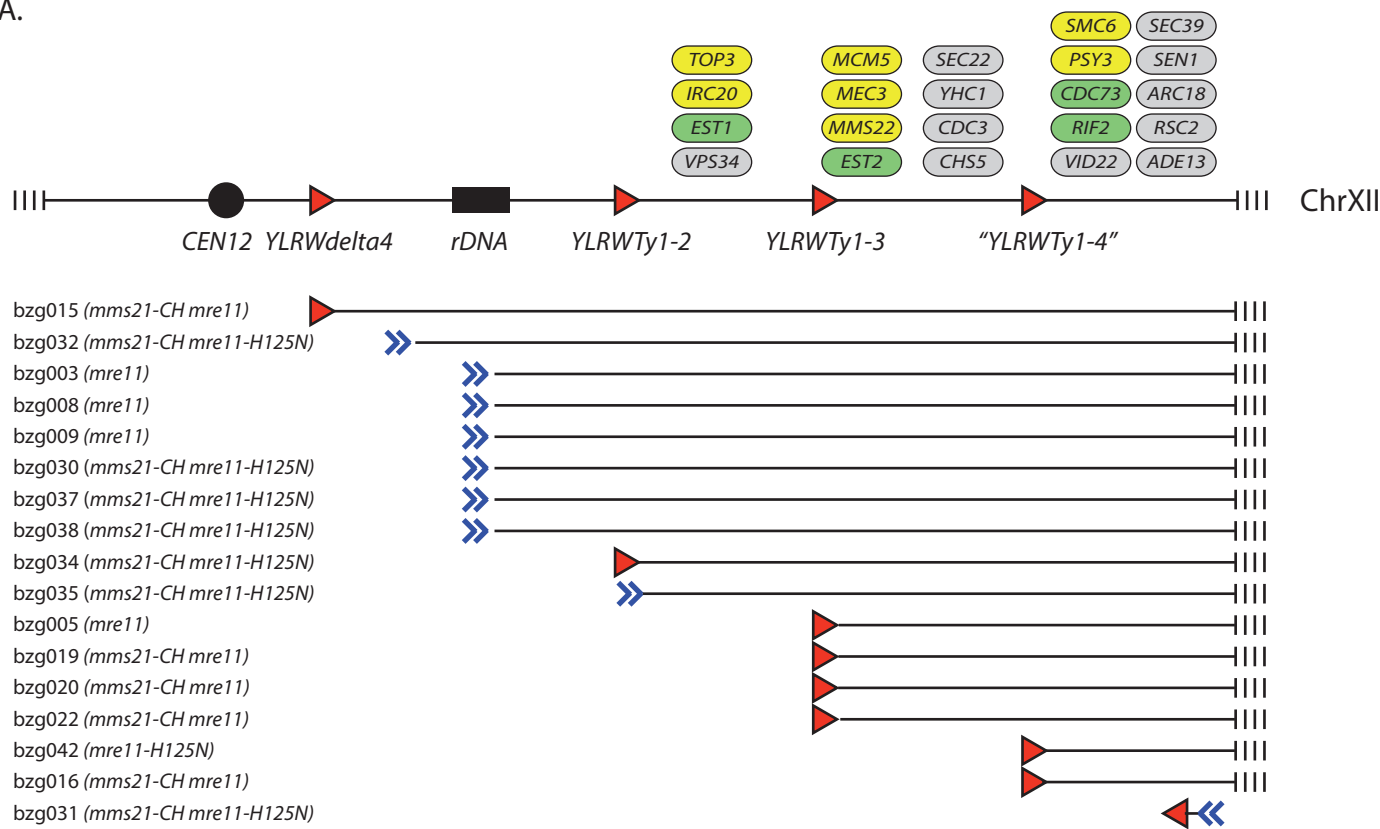

B.

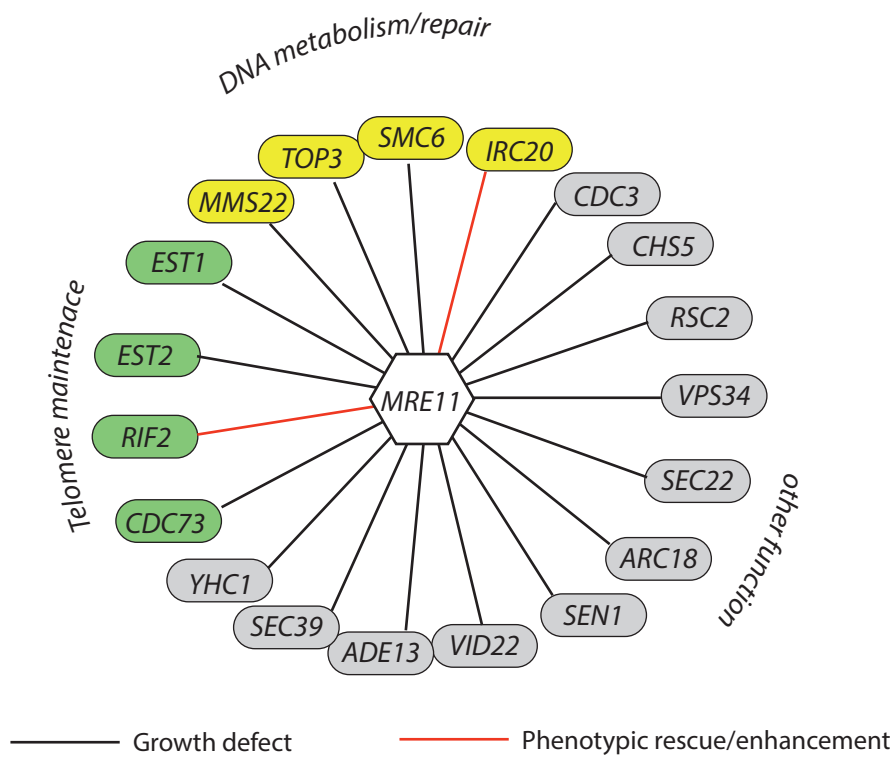

Supplement: S16 Fig — A. Strains with mutations affecting MRE11 show a propensity to duplicate regions of the right arm of chromosome XII. Duplicated regions from each sequenced isolate are drawn as horizontal lines with solid red triangles indicating Ty homology-mediated translocations and double blue chevrons indicating micro- or non-homology translocations. The centromere is depicted as a black circle, the rDNA repeats as a black box, and the telomeres as a series of vertical lines. Genes on chromosome XII R that are involved in DNA repair (yellow), telomere homeostasis (green), or genetically interact with MRE11 (grey) are depicted above the chromosomal region where they are encoded. B. Genetic interactions reported in BioGRID [76] between MRE11 and genes (color coded as above) encoded in the duplicated regions of chromosome XII R. Black lines indicate growth defects (synthetic lethality, synthetic growth defect, negative genetic E-MAP measurements), and red lines indicate phenotypic rescue or phenotypic enhancement. (PDF) [file pgen.1007250.s016.pdf]

S17 Figure

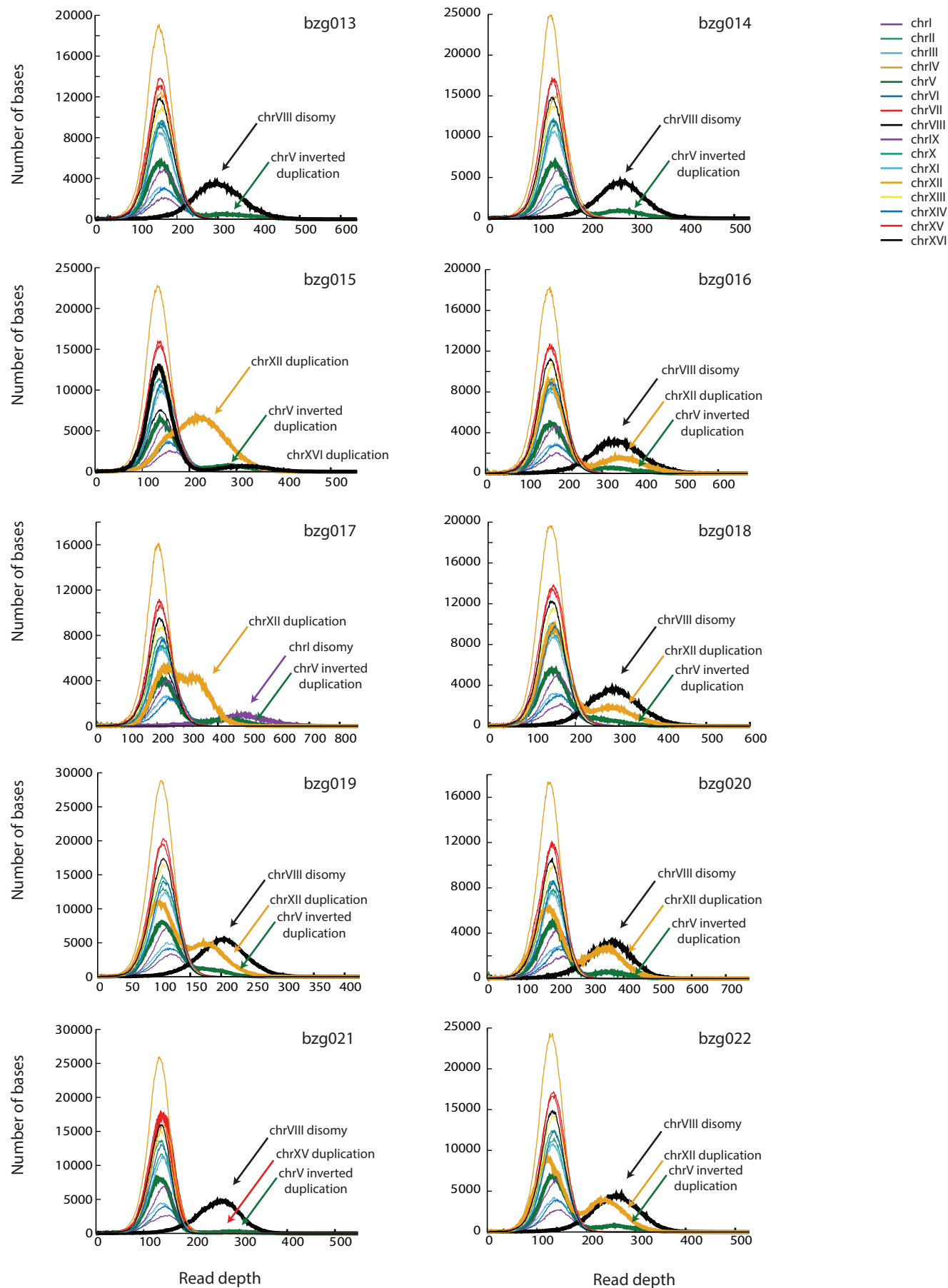

Supplement: S17 Fig — Copy number histograms for all sixteen chromosomes in the sequenced mms21-CH mre11Δ GCR-containing isolates are shown. Duplicated regions have twice the read depth as non-duplicated regions. Chromosomes duplicated by GCR-related events show a bimodal distribution. Chromosomes that are disomic such as chromosome VIII (bzg013, bzg014, bzg016, bzg018, bzg019, bzg020, bzg021, and bzg022) and chromosome I (bzg017) show a single peak at twice the read depth as most other chromosomes. No other disomic chromosomes were observed in these strains and no disomies were observed in any other strain analyzed. Note that the common chromosome VIII disomy cannot be attributed to the parental strain, as bzg013 and bzg015 to bzg022 were derived from one parental strain and bzg014 was derived from another parental strain. (PDF) [file pgen.1007250.s017.pdf]
