# Supplementary material for: SUMO E3 ligase Mms21 prevents spontaneous DNA damage induced genome rearrangements
Source: PLoS Genet. 2018 Mar 5;14(3):e1007250. doi: 10.1371/journal.pgen.1007250 (PMC5860785; doi:10.1371/journal.pgen.1007250)
Supplement: S3 Fig — Evidence for each junction is displayed as for S1 Fig. (PDF) [file pgen.1007250.s003.pdf]

S3 Figure

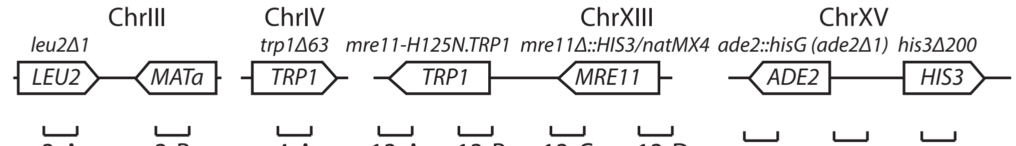

Junction:		3-A	3-B	4-A	13-A	13-B	13-C	13-D	15-A	15-B	15-C
wt	bzg001	1118/215	275/11	808/183	n.a.	n.a.	n.a.	n.a.	905/116	773/124	777/135
	bzg002	1081/234	234/11	718/165	n.a.	n.a.	n.a.	n.a.	781/118	646/116	680/158
mre11	bzg003	510/110	90/2	418/81	n.a.	n.a.	258/81	275/61	525/105	346/71	322/67
	bzg004	847/215	110/3	685/185	n.a.	n.a.	475/145	461/103	864/169	691/131	630/155
	bzg005	676/187	88/4	603/168	n.a.	n.a.	328/134	418/126	646/143	589/109	523/132
	bzg006	640/166	55/?	535/182	n.a.	n.a.	315/139	293/61	655/140	530/181	518/97
	bzg007	166/35	68/2	242/55	n.a.	n.a.	142/36	351/76	204/45	148/26	132/31
	bzg008	322/76	95/2	378/100	n.a.	n.a.	241/65	464/107	316/70	302/77	218/49
	bzg009	473/76	144/6	481/121	n.a.	n.a.	311/74	520/113	372/45	376/62	386/72
mms21-CH	bzg010	489/77	190/6	511/105	n.a.	n.a.	n.a.	n.a.	364/73	425/90	398/58
	bzg011	422/69	155/5	386/81	n.a.	n.a.	n.a.	n.a.	360/68	344/57	289/63
	bzg012	241/39	79/2	184/34	n.a.	n.a.	n.a.	n.a.	196/56	163/34	118/35
mms21-CH	bzg013	457/72	82/2	386/70	n.a.	n.a.	293/129	436/80	315/86	322/84	323/78
mre11	bzg014	353/64	7/4	310/66	n.a.	n.a.	250/96	302/146	269/63	276/100	250/41
	bzg015	346/76	75/0	322/16	n.a.	n.a.	226/114	335/141	250/69	236/53	225/54
	bzg016	449/77	56/3	363/96	n.a.	n.a.	277/141	404/173	307/83	289/161	306/60
	bzg017	550/95	81/2	451/111	n.a.	n.a.	374/163	417/210	382/98	372/72	384/63
	bzg018	384/85	56/5	330/65	n.a.	n.a.	246/111	398/174	297/80	272/68	270/43
	bzg019	255/51	3/0	211/52	n.a.	n.a.	144/93	214/130	198/44	145/44	152/50
	bzg020	471/93	81/12	445/61	n.a.	n.a.	296/132	405/195	327/90	337/88	306/81
	bzg021	236/65	54/0	265/60	n.a.	n.a.	162/125	212/112	267/61	260/58	237/51
	bzg022	346/55	69/3	286/69	n.a.	n.a.	205/92	299/153	235/63	222/53	223/56
mms21-CH	bzg030	488/94	n.a.	414/97	115/321	282/148	n.a.	n.a.	340/84	368/71	382/90
mre11-H125N	bzg031	504/101	n.a.	450/86	176/326	272/143	n.a.	n.a.	410/93	395/78	384/93
	bzg032	500/85	n.a.	338/93	160/286	247/143	n.a.	n.a.	395/83	349/63	308/74
	bzg033	447/55	n.a.	382/74	163/291	256/100	n.a.	n.a.	294/82	331/97	277/47
	bzg034	474/71	n.a.	401/88	161/312	262/137	n.a.	n.a.	358/74	398/64	385/80
	bzg035	481/96	n.a.	381/83	174/332	233/130	n.a.	n.a.	363/71	333/68	304/69
	bzg036	375/78	n.a.	290/64	114/256	200/105	n.a.	n.a.	253/62	243/67	271/46
	bzg037	549/85	n.a.	452/97	209/365	339/155	n.a.	n.a.	397/100	488/98	371/72
	bzg038	399/72	n.a.	244/58	105/222	185/115	n.a.	n.a.	249/68	244/61	252/62
	bzg039	520/97	n.a.	373/72	143/312	231/134	n.a.	n.a.	305/68	298/85	320/77
	bzg040	483/91	n.a.	369/66	152/285	226/137	n.a.	n.a.	349/74	353/71	291/72
	bzg041	601/140	n.a.	483/140	194/432	431/190	n.a.	n.a.	450/114	492/125	467/115
mre11-H125N	bzg042	393/81	63/2	246/62	94/189	175/112	n.a.	n.a.	239/72	199/58	192/44
	bzg043	577/88	125/0	444/71	204/332	320/153	n.a.	n.a.	332/93	431/105	393/69
	bzg044	664/111	198/3	583/19	281/348	392/176	n.a.	n.a.	474/97	584/122	594/101
	bzg045	597/110	162/3	393/73	233/316	339/137	n.a.	n.a.	348/67	385/64	326/63
	bzg046	644/104	173/7	412/79	241/312	404/144	n.a.	n.a.	450/83	434/72	434/62
	bzg047	615/93	111/9	349/73	178/279	276/143	n.a.	n.a.	294/70	306/46	311/61
