# Supplementary material for: SUMO E3 ligase Mms21 prevents spontaneous DNA damage induced genome rearrangements
Source: PLoS Genet. 2018 Mar 5;14(3):e1007250. doi: 10.1371/journal.pgen.1007250 (PMC5860785; doi:10.1371/journal.pgen.1007250)
Supplement: S11 Fig — A-Q. For most panels, the mapped junction sequence is displayed on top and the key hairpin-containing intermediate (see S10 Fig) is displayed on bottom. C. The inversion-containing sequence was derived by aligning all reads that paired with reads mapping to chrV:34,339–34,739 and pointed in a forward orientation (e.g. paired with a read in the chrV:34,339–34,739 region that pointed in the reverse orientation). This was necessary as the inversion site occurred next to the can1::hisG junction within a distance shorter than the median intra-read pair distance. H. The middle sequence is the engineered junction between CAN1 and YEL068C that introduces plasmid sequence not present in the reference S. cerevisiae genome, but is involved in the formation of the resected intermediate during hairpin formation for the mms21-CH mre11Δ GCR bzg015. I. The middle sequence is the engineered junction between hisG and the CAN1 flanking region in the can1::hisG locus that is not present in the reference S. cerevisiae genome, but is involved in hairpin formation for the mms21-CH mre11Δ GCR bzg016. O. For the mms21-CH mre11-H125N bzg034, a complete sequence of the inversion was not obtained; however, reads adjacent to the inversion could be aligned to generate two almost identical sequences, each of which defined one half of the inversion (see arrows at bottom). Q. The inversion-containing sequence was derived by aligning all reads that paired with reads mapping to chrV:34,339–34,739 and pointed in a forward orientation (e.g. paired with a read in the chrV:34,339–34,739 region that pointed in the reverse orientation). This was necessary as the inversion site occurred next to the can1::hisG junction within a distance shorter than the median intra-read pair distance. Arrows indicate the two copies of the inverted sequence. (PDF) [file pgen.1007250.s011.pdf]

A. Bzg001 (wild-type)

```
                         ChrV 35846-:
CATTACAGGAAAAGAGTAGCA:CCTGTAAATGATA:
||||||||||||||||||||| |||||||||||||
CATTACAGGAAAAGAGTAGCA:CCTGTAAATGATA:AATGCAGGTTCACGTGAACCAAA
                      ||||||| ||||| |||||||||||||||||||||||
                     :CCTGTAA-TGATA:AATGCAGGTTCACGTGAACCAAA
                     :ChrV 35871+
```

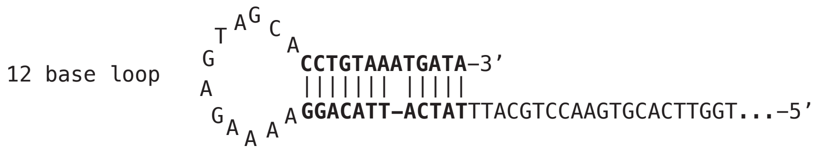

B. Bzg004 (*mre11*)

```
                   ChrV 42524-:
CTGCCGGCTGAAAGAGCTT:GTGCAATG:GGAGTGCCAA
||||||||||||||||||| ||||||||
CTGCCGGCTGAAAGAGCTT:GTGCAATG:CAGTGTTCAAAAAGCCTCTTAAAAGGCG
                    |||||||| ||||||||||||||||||||||||||||
     GGCTGATTTGTCGG:GTGCAATG:CAGTGTTCAAAAAGCCTCTTAAAAGGCG
                   :ChrV 42453+
```

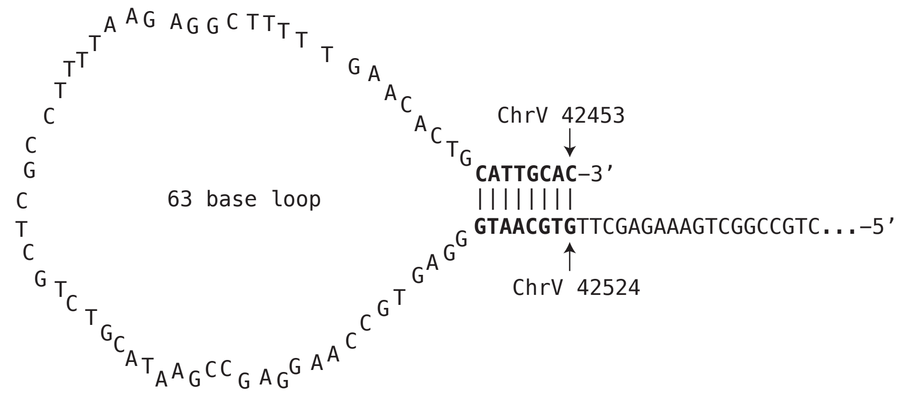

C. Bzg005 (mre11), Bzg018, Bzg019 (mms21-CH mre11), Bzg033, Bzg041 (mms21-CH mre11-H125N)
Bzg043, Bzg044, Bzg045,Bzg046 (mre11-H125N)

```
5’-...CAGATCTTCCAGTGGTGCATGAACGCATGAGAAAGCCCCCGGAAGATGATCTTCCGGGGGCTTTCTCATGCGTTCATGCACCACTGG
3’-...GTCTAGAAGGTCACCACGTACTTGCGTACTCTTTCGGGGGCCTTCTACTAGAAGGCCCCCGAAAGAGTACGCAAGTACGTGGTGACC

   AAGATCTGAATTCTTGAAGACGAAAGGGCCTCGTGATACGCCGGGGGATCC:CTGGGTTAGCTTGAAGCGACTTTCTTTCTCTACT...-3’
   TTCTAGACTTAAGAACTTCTGCTTTCCCGGAGCACTATGCGGCCCCCTAGG:GACCCAATCGAACTTCGCTGAAAGAAAGAGATGA...-5’
                                                      :ChrV 34339+


                   ATCTTCCGGGGGCTTT-3’
1 base loop      C ||||||||||||||||
                   TAGAAGGCCCCCGAAAGAGTACGCAAGTACGTGGTGACCTTCTAGACTTAAGAACTTCTGCTTTCCCGGAGCAC...-5‘
```

S11 Figure

D. Bzg006 (*mre11*)

```
                              CDP019035.1 2049119-:
TTCAGGCGCGCGAATCGAAATACATCAT:GATGCACGCGCC:
|||||||||||||||||||||||||||| ||||||||||||
TTCAGGCGCGCGAATCGAAATACATCAT:GATGCACGCGCC:TGAATCACGCCCTGAATACGGGTCAG
                              ||| | |||||| |||||||||||||||||||||||||||
                             :GATTCGCGCGCC:TGAATCACGCCCTGAATACGGGTCAG
                             :CDP019035.1 2049143+
```

```
                    T  C  A
                 A          T
                C            GATGCACGCGCC-3
12 base loop                 ||| | ||||||
                 A           CTAAGCGCGCGGACTTAGTGCGGGACTTATGCCCAGTCGTTAAATAGCTAGTCGACA...-5’
                  T A A A G
```

E. Bzg007 (*mre11*)

```
                           CP019035.1 2049078-:
GAACGCCTGGAAGAGGTTATCGCCCTG:CTGCCAGGCG:
||||||||||||||||||||||||||| ||||||||||||||
GAACGCCTGGAAGAGGTTATCGCCCTG:CTGCCAGGCG:TTCACTTGGCGCGTGCATCATGATGTATTTCGAT
                             || ||||||| ||||||||||||||||||||||||||||||||||||
                           :CTTCCAGGCG:TTCACTTGGCGCGTGCATCATGATGTATTTCGAT
                           :CP019035.1 2049102+
```

```
                  G C C C T
                 C         G
                             CTGCCAGGCG-3’
14 base loop     T           || ||||||
                 A          AGAAGGTCCGCAAGTGAACCGCGCACGTAGTACTACATAAAGC...-5’
                  T T G G
```

F. Bzg013 (*mms21-CH mre11*)

```
                                   :ChrV 42277+
                                   :CCCGGGACCCCGGG:CATAAGTGCATAGTAGGCACAGACCTGA
                                    |||||||||||||| ||||||||||||||||||||||||||||
GACAGATCAGGTCTGTGCCTACTATGCACTTATG:CCCGGGACCCCGGG:CATAAGTGCATAGTAGGCACAGACCTGA
|||||||||||||||||||||||||||||||||| |||||| |||||||
GACAGATCAGGTCTGTGCCTACTATGCACTTATG:CCCGGGGTCCCGGG:
                                      ChrV 42277-:
```

```
                T CCCGGG-3’
2 base loop       ||||||
                G GGGCCCGTATTCACGTATCATCCGTGTCTGGACTAGACAGCCCACGTCAAAT...-5’
```

G. Bzg014 (*mms21-CH mre11*)

```
                                         ChrV 27348-:
TGCACCTGGATTAAGCTGCAAGTCATTCTGT:GCCTGCGCCGTACGGCGC:TTGC
||||||||||||||||||||||||||||||| ||  ||||||||||||||
TGCACCTGGATTAAGCTGCAAGTCATTCTGT:GCAAGCGCCGTACGGCGC:AGGCACAGAATGACTTGCAG
                                 ||||||||||||||||| |||||||||||||||||||
                                :GCAAGCGCCGTACGGCGC:AGGCACAGAATGACTTGCAG
                                :ChrV 27344+
```

```
                ( ACGGCGCTTGC-3’
0 base loop     ( |||||||  ||
                ( TGCCGCGTCCGTGTCTTACTGAACGTCGAATTAGGTCCACGTAAAATTC...-5’
```

S11 Figure

H. bzg015 (*mms21-CH mre11*)

```
                       Chr V 25817-:
AGAGATTTTGGAGCGTAAAACTACCACCAGTGAAT:
|||||||||||||||||||||||||||||||||||
AGAGATTTTGGAGCGTAAAACTACCACCAGTGAAT:GCTAGCACGGCCGCCAGTGTGCTGG:AATTCACTGGTGGTAGTTTTACGCT
                                                              |||||||||||||||||||||||||
                                                             :AATTCACTGGTGGTAGTTTTACGCT
                                                             :ChrV 25816+
```

Starting sequence (engineered *CAN1/YEL068C* junction)

```
                                                 ChrV 30951-:
CAN1:    TTCTTGGTGCCGGAATAATTACTCAGCCGTTCGCGATCAAAAATGCTGGTA:
         |||||||||||||||||||||||||||||||||||||||||||||||||||
Junct:   TTCTTGGTGCCGGAATAATTACTCAGCCGTTCGCGATCAAAAATGCTGGTA:AGGGCG


Junct:   AATTCCAGCACACTGGCGGCCGTGCTAGC:ATTCACTGGTGGTAGTTTTACGCTCCAA
                                       ||||||||||||||||||||||||||||
YEL068C:                              :ATTCACTGGTGGTAGTTTTACGCTCCAA
                                      :ChrV 25817+

Junct:   AATCTCTTATCATA
         ||||||||||||||
YEL068C: AATCTCTTATCATA

                 C C  G
                C      G
                        GTGCTGGA ATT-3'
10 base loop   G        ||||| |  |||
                C      TCACGATCG:TAAGTGACCACCATCAAAATGCGAGGTTTTAGAGAATAGTAT...-5'
                 C A G
```

I. bzg016 (*mms21-CH mre11*)

```
2,048,825           CP019035.1 2,048,785-:
TAAAGACCATGAGCTTCAATACCCTGATTGACTGGAACAGC:
|||||||||||||||||||||||||||||||||||||||||
TAAAGACCATGAGCTTCAATACCCTGATTGACTGGAACAGC:CGGATCTGCCCGGGGGATCCGGCCAGATCCG:GCTGTTCCAGTCAAT
                                                                          |||||||||||||||
                                                                         :GCTGTTCCAGTCAAT
                                                                         :2,048,785+

CAGGGTATTGAAGCTCATGGTCTTTACTCCATCACAAGGTTCCGCCTTATCCGGC
|||||||||||||||||||||||||||||||||||||||||||||||||||||||
CAGGGTATTGAAGCTCATGGTCTTTACTCCATCACAGGGTTCCGCCTTATCCGGC
                                              2,048,854
```

Starting sequence (engineered *can1::hisG* junction)

```
                                   ChrV 30,928+:
5'-...ATAAGTCTTAACGTCCAATCAACTATAAATCCGAGGGCTACG:CTCGAGGTCGACGGTATCGATAAGCTTGATATCGAATTCCTGCA
      |||||||||||||||||||||||||||||||||||||||||| ||||||||||||||||||||||||||||||||||||||||||||
3'-...TATTCAGAATTGCAGGTTAGTTGATATTTAGGCTCCCGATGC:GAGCTCCAGCTGCCATAGCTATTCGAACTATAGCTTAAGGACGT


      GCCCGGGGGATCCGGCCAGATCCG:GCTGTTCCAGTCAATCAGGGTATTGAAGCTCATGGTCTTTACTCCAGCACAGGGTTCCGCCT...-3'
      |||||||||||||||||||||||| ||||||||||||||||||||||||||||||||||||||||||||||||||||||||||||||
      CGGGCCCCCTAGGCCGGTCTAGGC:CGACAAGGTCAGTTAGTCCCATAACTTCGAGTACCAGAAATGAGGTCGTGTCCCAAGGCGGA...-5'
                              :CP019035.1 2,048,785+

                C C CCGGGC-3'
5 base loop    C     |||| |
                T A GGCCGGTCTAGGCCGACAAGGTCAGTTAGTCCCATAACTTCGAGTACCAGAAATGAGGTCGTGTCCCAAGGCGGA...-5'
```

S11 Figure

J. bzg017 (*mms21-CH mre11*)

```
                                        ChrV 30,866–:
ATTTATAGTTGATTGGACGTTAAGACTTATTGTCATTAACTTGA:CTCTTGC:
|||||||||||||||||||||||||||||||||||||||||||| |||||||
ATTTATAGTTGATTGGACGTTAAGACTTATTGTCATTAACTTGA:CTCTTGC:CAGCAAGAGTCAAGTTAATGACAATAAGTCTTAA
                                             ||||||| ||||||||||||||||||||||||||||||||||
                                            :CTCTTGC:CAGCAAGAGTCAAGTTAATGACAATAAGTCTTAA
                                            :ChrV 30,857+
```

```
             G GCAAGAG–3’
2 base loop    |||||||
             T CGTTCTCAGTTCAATTACTGTTATTCAGAATTGCAGGTTAGTTGATATTTAGGCTCCCGATGCATACTATTATCGGGCGG...–5’
```

K. bzg020 (*mms21-CH mre11*)

```
                                     ChrV 29908–:
ACACAAATTTGTTGTTAACTAGTAAGAAGAAGAGTTGGAAG:GAAAGG:
||||||||||||||||||||||||||||||||||||||||| ||||||
ACACAAATTTGTTGTTAACTAGTAAGAAGAAGAGTTGGAAG:GAAAGG:AAGCCTTTCCTTCCAACTCTTCTTCTTACTAGT
                                          |||||| |||||||||||||||||||||||||||||||||
                                         :GAAAGG:AAGCCTTTCCTTCCAACTCTTCTTCTTACTAGT
                                         :ChrV 29899+
```

```
               T CCTTTC–3’
3 base loop  T   ||||||
               C GGAAAGGAAGGTTGAGAAGAAGAATGATCAATTGTTGTTTAAACACATACCGCCGT...–5’
```

L. bzg021 (*mms21-CH mre11*)

```
                                       ChrV 26469–:
AGGCCTTACGCAGAGTATGCAATGTACCGCCTGATCATCAGCT:GATCGC:
||||||||||||||||||||||||||||||||||||||||||| ||||||
AGGCCTTACGCAGAGTATGCAATGTACCGCCTGATCATCAGCT:GATCGC:ACTGAAGCGATCAGCTGATGATCAGGCGGTACATTGCA
                                            |||||| ||||||||||||||||||||||||||||||||||||||
                                           :GATCGC:ACTGAAGCGATCAGCTGATGATCAGGCGGTACATTGCA
                                           :ChrV 26457+
```

```
                G T
               A     GCGATC–3’
6 base loop          ||||||
                C T T CGCTAGTCGACTACTAGTCCGCCATGTAACGTATGAGACGCATTCCGGAAAAAGTGGTTAACTGGCGTAGCATATAAAGGGT...–5’
```

M. bzg022 (*mms21-CH mre11*)

```
                                     ChrV 28844–:
TGAACCCGAGGATTGTACCAACGATAACAGAAACAAATGAA:TACAAG:
TGAACCCGAGGATTGTACCAACGATAACAGAAACAAATGAA:TACAAG:AATTACCTCCTTGTATTCATTTGTTTCTGTTATCGTTGGT
                                          |||||| ||||||||||||||||||||||||||||||||||||||||
                                         :TACAAG:AATTACCTCCTTGTATTCATTTGTTTCTGTTATCGTTGGT
                                         :ChrV 28829+
```

```
                 A T T
                A       CTTGTA–3’
9 base loop     T       ||||||
                G       GAACATAAGTAAACAAAGACAATAGCAACCATGTTAGGAGCCCAAGTATGACTAATTTCAAT...–5’
                 G A G
```

S11 Figure

N. bzg034 (*mms21-CH mre11-H125N*)

```
                                  ChrV 27,091-:
GGTGATTGTAATGGTCTGTAACCCAGTACACTG:CAGGCGTACCGA:
||||||||||||||||||||||||||||||||| ||| ||| ||||
GGTGATTGTAATGGTCTGTAACCCAGTACACTG:CAGCCGTGCCGA:TGAGCTCGGTACGCCTGCAGTGTACTGGGTTACAG
                                  |||||||||||| |||||||||||||||||||||||||||||||||||
                                 :CAGCCGTGCCGA:TGAGCTCGGTACGCCTGCAGTGTACTGGGTTACAG
                                 :ChrV 27,074+
```

```
                C A TCGGCACGGCTG-3'
5 base loop  T     |||| ||| |||
                C G AGCCATGCGGACGTCACATGACCCAATGTCTGGTAATGTTAGTGGCGCGTGTCAACGACATGTG...-5'
```

O. bzg035 (*mms21-CH mre11-H125N*)

Two halves of the inversion junction

1st half sequence

```
                                                               chrV 33115 CC:
Query: 67    ATGCCAAAGAACCCATAAATAAATATGATATAAGAGCGCCCACTGGGCC:AACGCCGGCCC 8
             |||||||||||||||||||||||||||||||||||||||||||||||||   ||  || |
Sbjct: 33067 ATGCCAAAGAACCCATAAATAAATATGATATAAGAGCGCCCACTGGGCC:GGCGTTGGTC- 33125

Query: 7     AGTGG 3
             || ||
Sbjct: 33126 AGAGG 33130
```

2nd half sequence

```
                                                                chrV 33123 GG:
Query: 64    CCAAAGAACCCATAA-TAAATATGATATAAGAGCGCCCACTGGGCCGGCGTTGG:CCCAGT 6
             ||||||||||||||| ||||||||||||||||||||||||||||||||||||||  | ||
Sbjct: 33070 CCAAAGAACCCATAAATAAATATGATATAAGAGCGCCCACTGGGCCGGCGTTGG:TC-AGA 33128

Query: 5     GG 4
             ||
Sbjct: 33129 GG 33130
```

```
              T T GG-3'
6 base loop  G    ||
             C G GCCGGGTCACCCGCGAGAATATAGTATAAATAAATACCCAAGAAACCGTA...-5'
```

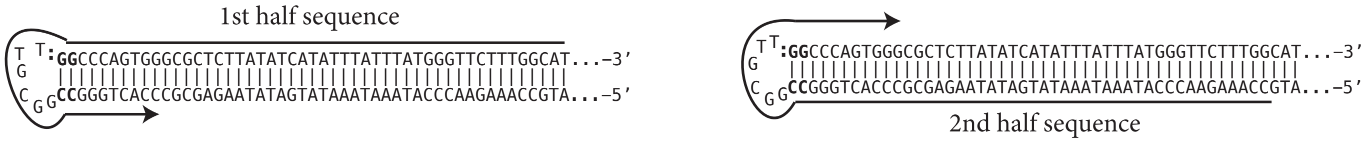

S11 Figure

P. bzg040 (*mms21-CH mre11-H125N*)

```
                                      ChrV 29,524-:
ATGTATGAAATTATTTGGGTGAGATAATATTAATCTTTCCTAT:CTTTAC:
||||||||||||||||||||||||||||||||||||||||||| ||||||
ATGTATGAAATTATTTGGGTGAGATAATATTAATCTTTCCTAT:CTTTAC:AAGCAGTAAAGATAGGAAAGATTAATATTA
                                            |||||| ||||||||||||||||||||||||||||||
                                           :CTTTAC:AAGCAGTAAAGATAGGAAAGATTAATATTA
                                           :ChrV 29,513+
```

```
                T T GTAAAG–3’
5 base loop   C     ||||||
                G T CATTTCTATCCTTTCTAATTATAATAGAGTGGGTTTATTAAAGTATGTACGTGTATATCGATGATGTATCA...–5’
```

Q. bzg042 (*mre11-H125N*)

Recovered junction

```
CCC:GGCGTATCACGAGGCCCTTTCGTCTTCAAGAATTCAG:ATCTTCCA:GTGGTGGAAGAT:CTGAATTCTTGAAGACGAAAGGGCCTCGTGATACGCC:GGGGGATCCCTGG
                                                                                                        :ChrV 34,339+
                                                                                         hisG+polylinker:
```

```
4 base loop

G GTG–3’
   ||
T G ACCTTCTAGACTTAAGAACTTCTGCTTTCCCGGAGCACTATGCGGCCCCCTAGG:GACCCAATCGAACTTCGCTGAAAGAAAGAGATGATTTCCCTTA...–5’
```

R. bzg047 (*mre11-H125N*)

```
                                  ChrV 26,457–:
ATGCAATGTACCGCCTGATCATCAGCTGATCGCTTCAGT:GCGATC:
||||||||||||||||||||||||||||||||||||||| ||||||
ATGCAATGTACCGCCTGATCATCAGCTGATCGCTTCAGT:GCGATC:AGCTGATGATCAGGCGGTACATTGCATACTCTGCGTAAGGCC
                                        |||||| ||||||||||||||||||||||||||||||||||||||||||
                                       :GCGATC:AGCTGATGATCAGGCGGTACATTGCATACTCTGCGTAAGGCC
                                       :ChrV 26,469+
```

```
                 G T GCGATC–3’
6 base loop    A      ||||||
                C
                  T T CGCTAGTCGACTACTAGTCCGCCATGTAACGTATGAGACGCATTCCGGAAAAAGTGGTTAACTGGCGTAGCATA...–5’
```
