# Supplementary material for: SUMO E3 ligase Mms21 prevents spontaneous DNA damage induced genome rearrangements
Source: PLoS Genet. 2018 Mar 5;14(3):e1007250. doi: 10.1371/journal.pgen.1007250 (PMC5860785; doi:10.1371/journal.pgen.1007250)
Supplement: S12 Fig — Sequence of the junction between YELWdelta6 (yellow) and YLRWdelta6 (red) that fuses the inverted duplication on chromosome V (magenta) with chromosome XII (grey). Sequence that could have been derived from either YELWdelta6 or YLRWdelta6 is displayed with an orange background. (PDF) [file pgen.1007250.s012.pdf]

S12 Figure

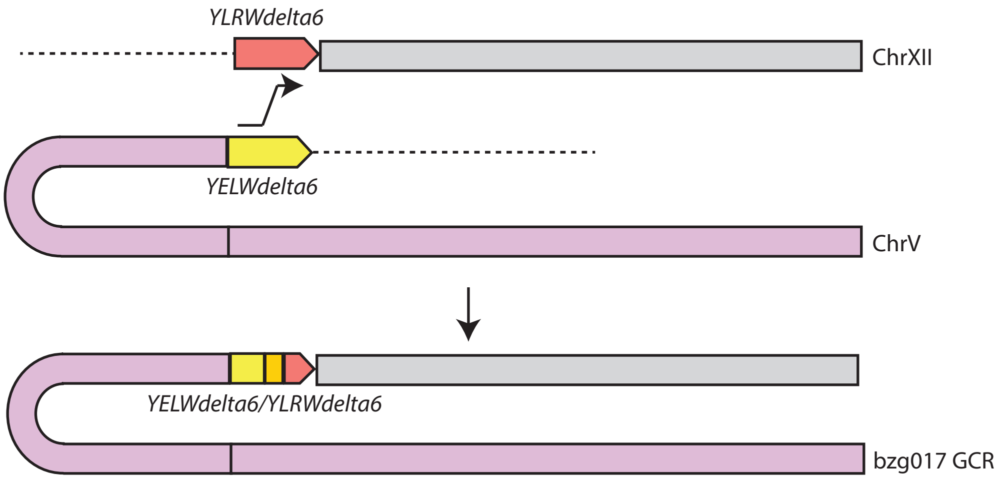


```
ChrV 137949     ATGATGATTCCATTATTGCGAATAAAGGTATTTCTGAAAGTACATCTAATGGGCTATTTG
                ||||||||||||||||||||||||||||||||||||||||||||||||||||||||||||
Junction        ATGATGATTCCATTATTGCGAATAAAGGTATTTCTGAAAGTACATCTAATGGGCTATTTG

ChrV 138009     AAAATTTGTTTAGGATTATGGTAATAAAAGAGGCATTAACGCCAGAACCTTTATTTTATC
                ||||||||||||||||||||||||||||||||||||||||||||||||||||||||||||
Junction        AAAATTTGTTTAGGATTATGGTAATAAAAGAGGCATTAACGCCAGAACCTTTATTTTATC

ChrV 138069     ACTTTTTCGATATTTGTTAATAATCTAAATCTAACTTATATCTAAACCTTAGCGTTTGCA
                ||||||||||||||||||||||||||||||||||||||||||||||||||||||||||||
Junction        ACTTTTTCGATATTTGTTAATAATCTAAATCTAACTTATATCTAAACCTTAGCGTTTGCA

ChrV 138129     TTCTTGATTTCTATATTTTTATTCACAATATGGAAAGGAAAAACAATCTCTTAGGTTGTC
                ||||||||||||||||||||||||||||||||||||||||||||||||||||||||||||
Junction        TTCTTGATTTCTATATTTTTATTCACAATATGGAAAGGAAAAACAATCTCTTAGGTTGTC

ChrV 138189     CAAGAAAACTTCTCTCCCTGTCATACTCAAAGTGTTGGAACAAAAATCAACTATCGTCTA
                ||||||||||||||||||||||||||||||||||||||||||||||||||||||||||||
Junction        CAAGAAAACTTCTCTCCCTGTCATACTCAAAGTGTTGGAACAAAAATCAACTATCGTCTA

ChrV 138249     TCAACTAGTAGCTATACTACTAATATATTATCATATACGGTGTTAGATGATGACATAAGT
                ||||||||||||||||||||||||||||||||||||||||||||||||||||||||||||
Junction        TCAACTAGTAGCTATACTACTAATATATTATCATATACGGTGTTAGATGATGACATAAGT

ChrV 138309     TATGAGAAGCTGTCATCGAAGTTAGAAGAAGCTGAAATGCAAGGATTGATAATGTAATAG
                ||||||||||||||||||||||||||||||||||||||||||||||||||||||||||||
Junction        TATGAGAAGCTGTCATCGAAGTTAGAAGAAGCTGAAATGCAAGGATTGATAATGTAATAG
                                           |||||||||||||||||||||||| ||||||||
ChrXII 448948                              GAAGCTGAAATGCAAGGATTGATAGTGTAATAG

ChrV 138369     A:AT:TTAATGAAACATATAAAACG
                | ||   ||||||||||||||||||
Junction        A:AT:--AATGAAACATATAAAACGAAAAGAGAAATAATCATAACATTATTTCCTAGAGT
                  ||   |||||||||||||||||||||||||||||||||||||||||||||||||||||
ChrXII 448981   G:AT:--AATGAAACATATAAAACGAAAAGAGAAATAATCATAACATTATTTCCTAGAGT

Junction        AGCGATTCCCCTTCGCGTATTCTTACATCTTCGAAGAGAACTTCTGGTGTAAGTATAATA
                ||||||||||||||||||||||||||||||||||||||||||||||||||||||||||||
ChrXII 449037   AGCGATTCCCCTTCGCGTATTCTTACATCTTCGAAGAGAACTTCTGGTGTAAGTATAATA

Junction        AATATTATAGCTCTATCGAATGGTGCAATTATTTACCAAATTCTCAATAGGAATCCATAA
                ||||||||||||||||||||||||||||||||||||||||||||||||||||||||||||
ChrXII 449097   AATATTATAGCTCTATCGAATGGTGCAATTATTTACCAAATTCTCAATAGGAATCCATAA

Junction        TACTACATACGATACTAATATTCTAGTATTTTTATACTTATTATTTCTTTTTTATTACAC
                ||||||||||||||||||||||||||||||||||||||||||||||||||||||||||||
ChrXII 449157   TACTACATACGATACTAATATTCTAGTATTTTTATACTTATTATTTCTTTTTTATTACAC
```
