# Supplementary material for: SUMO E3 ligase Mms21 prevents spontaneous DNA damage induced genome rearrangements
Source: PLoS Genet. 2018 Mar 5;14(3):e1007250. doi: 10.1371/journal.pgen.1007250 (PMC5860785; doi:10.1371/journal.pgen.1007250)
Supplement: S14 Fig — Sequence of the junction between YELWdelta6 (yellow) and ura3-52 (red). Sequence that could have been derived from either YELWdelta6 or ura3-52 is displayed with an orange background. (PDF) [file pgen.1007250.s014.pdf]

S14 Figure

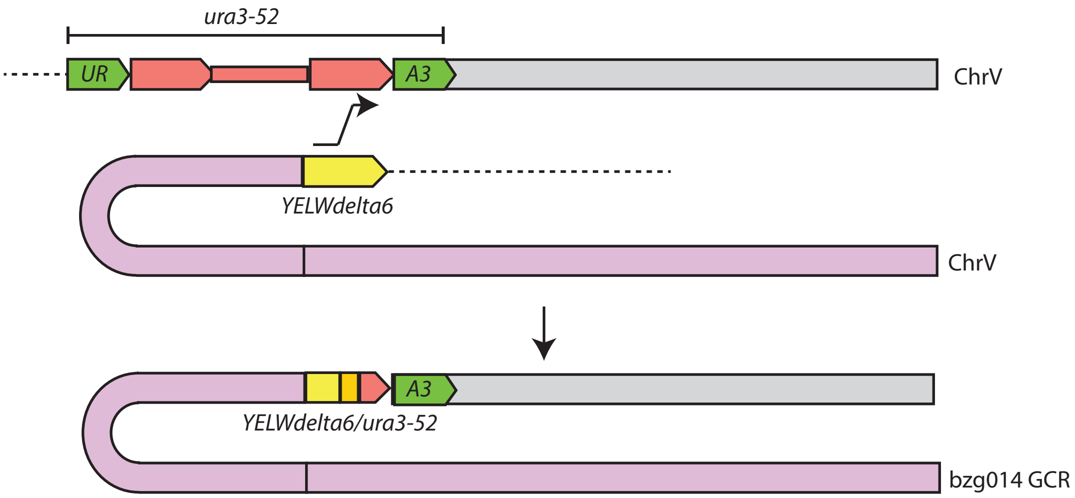


```
chrV 138350   AGGATTGATAATGTAATAGAATTTAATGAAACATATAAAACGGAATGAGGAATAATCGTA
              ||||||||||||||||||||||||||||||||||||||||||||||||||||||||||||
bzg014        AGGATTGATAATGTAATAGAATTTAATGAAACATATAAAACGGAATGAGGAATAATCGTA

chrV 138410   ATATTAGTATGTAAAAATATGGATTCCATTTTGAGGATTCCTATATCCATGAGGAGAACT
              ||||||||||||||||||||||||||||||||||||||||||||||||||||||||||||
bzg014        ATATTAGTATGTAAAAATATGGATTCCATTTTGAGGATTCCTATATCCATGAGGAGAACT
              |||  | | |||| || |   |||||| ||||  ||||||||| |||| |||||||||||
ura3-52       ATAGAATTGTGTAGAATTGCAGATTCCCTTTTATGGATTCCTAAATCCTTGAGGAGAACT

chrV 138470   TCTAGTATATGCTGTATACATAATACTATAGCCTTG:ATCAACAATGGAACCCCAACAATT
              |||||||||||||||||||||||||||||||||||| ||||||||||||||||||||||||
bzg014        TCTAGTATATGCTGTATACATAATACTATAGCCTTG:ATCAACAATGGAACCCCAACAATT
              |||||||||| |||||||| ||||| |||||||||  ||||||||||||||||||||||||
ura3-52       TCTAGTATATTCTGTATACCTAATATTATAGCCTTT:ATCAACAATGGAATCCCAACAATT

chrV 138530   ATCTCA:CAATTCACCCATTTCTCA:
              ||||||   ||||||  ||||||||
bzg014        ATCTCA:ACATTCACATATTTCTCA:GTACCACCAAG
              |||||| |||||||||||||||||| |||||||||||
ura3-52       ATCTCA:ACATTCACATATTTCTCA:GTACCACCAAG

bzg014        GAATTACTGGAGTTAGTTGAAGCATTAGGTCCCAAAATTTGTTTACTAAAAACACATGTG
              ||||||||||||||||||||||||||||||||||||||||||||||||||||||||||||
chrV 116293   GAATTACTGGAGTTAGTTGAAGCATTAGGTCCCAAAATTTGTTTACTAAAAACACATGTG

bzg014        GATATCTTGACTGATTTTTCCATGGAGGGCACAGTTAAGCCGCTAAAGGCATTATCCGCC
              ||||||||||||||||||||||||||||||||||||||||||||||||||||||||||||
chrV 116353   GATATCTTGACTGATTTTTCCATGGAGGGCACAGTTAAGCCGCTAAAGGCATTATCCGCC

bzg014        AAGTACAATTTTTTACTCTTCGAAGACAGAAAATTTGCTGACATTGGTAATACAGTCAAA
              ||||||||||||||||||||||||||||||||||||||||||||||||||||||||||||
chrV 116413   AAGTACAATTTTTTACTCTTCGAAGACAGAAAATTTGCTGACATTGGTAATACAGTCAAA
```
