# Supplementary material for: SUMO E3 ligase Mms21 prevents spontaneous DNA damage induced genome rearrangements
Source: PLoS Genet. 2018 Mar 5;14(3):e1007250. doi: 10.1371/journal.pgen.1007250 (PMC5860785; doi:10.1371/journal.pgen.1007250)
Supplement: S15 Fig — A. Analysis of copy number changes for GCRs bzg016, bzg018, and bzg042 using the S288c reference genome indicates that the duplication on chromosome XII begins adjacent to YLRCdelta21, which is in the opposite orientation relative to ura3-52 required to generate the breakpoint junction by HR. B. To understand the nature of the target region, read pairs with one read mapping to either side of YLRCdelta21 were analyzed in a sequenced uGCR strain that lacked a chromosome XII rearrangement (RDKY6761, [36]). First, read pairs in which one read mapped to Anchor region A (chrXII:817,711–818,470) preceding YLRCdelta21 were collected, and all reads that pointed in the reverse direction (e.g. reads that paired with a read that mapped in the forward direction in Anchor region A) were aligned. This alignment was identical to chrXII:817,711–818,470; however, the end of alignment did not map to chromosome XII adjacent to this region, but rather mapped to many delta sequences throughout the genome. Second, Anchor region B (chrXII:818,426–818,800) following YLRCdelta21 was analyzed using alignments from all reads pointing in the forward direction. This generated two alignments. The first alignment was identical to chrXII:818,470–818,849 but was preceded by a sequence containing the 3’ end of Ty sequence. The second alignment, derived from reads mapping to chrXII:818,387–818,467 were identical to the reference chromosome. Taken together, these junction sequences are consistent with the insertion of a full-length Ty in the forward orientation; called here “YLRWTy1-4” as the sequence of this element is most similar to other Ty1 elements in the S288c reference genome. The presence of this forward-oriented Ty element resolves the orientation problem for the formation of the chromosome V:XII translocation in GCRs bzg016, bzg018, and bzg042, and this Ty element has been previously observed in other strains derived in the S288c background [38,39]. C. A partial sequence of the junctions bet [file pgen.1007250.s015.pdf]

S15 Figure

A.

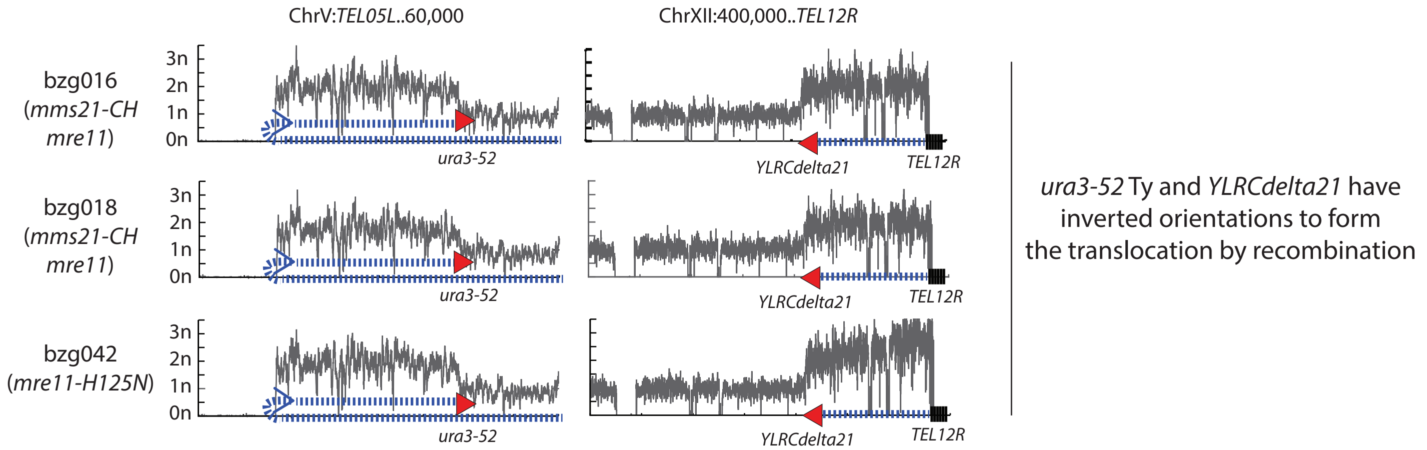

B.

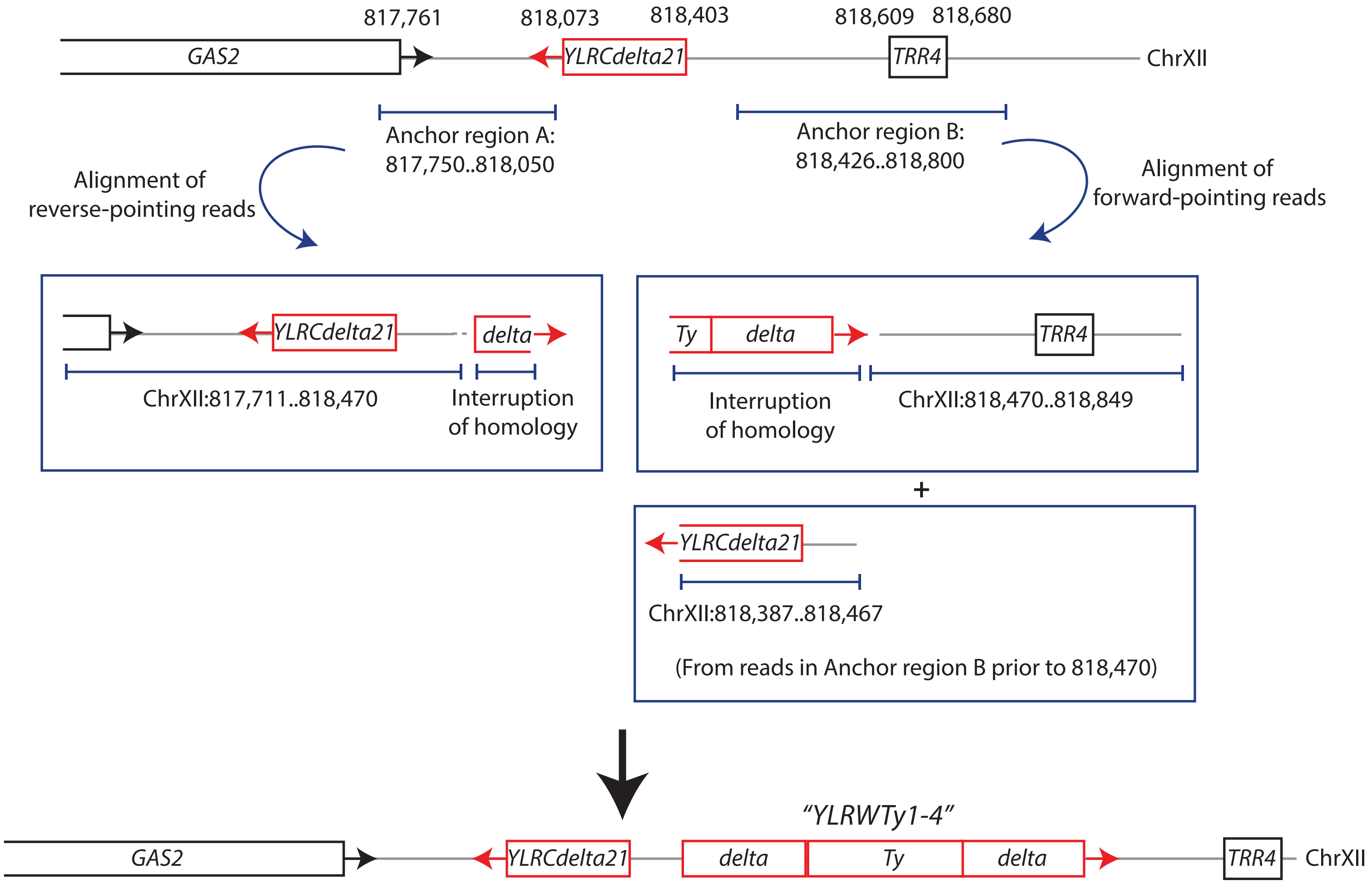

C.

```
TTTAAACTTGATTAATACTTTTTTTT:TGTTGGAATAAAAATCCACTATCG...AACAATTATCTCAACATTCACATATTTCTCA:TTTTTATACCTTTAACTATCATCATCAAT
TTTAAACTTGATTAATACTTTTTTTT:                                                              :TTTTTATACCTTTAACTATCATCATCAAT
            ChrXII 818,474+:                                                              :ChrXII 818,470+
```
