# Supplementary material for: SUMO E3 ligase Mms21 prevents spontaneous DNA damage induced genome rearrangements
Source: PLoS Genet. 2018 Mar 5;14(3):e1007250. doi: 10.1371/journal.pgen.1007250 (PMC5860785; doi:10.1371/journal.pgen.1007250)
Supplement: S1 Table — (PDF) [file pgen.1007250.s018.pdf]

S1 Table. Fluctuation results of double mutants containing a null mutation of genes in the Rad52 pathway and *mms21-CH.*

Genotype	uGCR rate*	dGCR rate*	Ratio #
Wild type**	$2.27x10^{-9}$ (1)<br>[$1.30x10^{-9}$ – $4.78x10^{-9}$]	$1.97x10^{-8}$ (8.7)<br>[$1.61x10^{-8}$ – $4.32x10^{-8}$]	8.7
*mms21-CH***	$2.08x10^{-8}$ (9.1)<br>[$0.00x10^{-8}$ – $18.2x10^{-8}$]	$4.2x10^{-6}$ (1850)<br>[$2.4x10^{-6}$ – $22.2x10^{-6}$]	202
*rad52Δ***	$1.67x10^{-8}$ (7.4)<br>[$0.99x10^{-8}$ – $2.74x10^{-8}$]	$1.09x10^{-8}$ (4.8)<br>[$0.29x10^{-8}$ – $7.08x10^{-8}$]	0.7
rad52Δ mms21-CH	$1.66x10^{-7}$(73)<br>[$2.62x10^{-8}$ – $5.64x10^{-7}$]<br>{HZY3310/3311}	$1.20x10^{-7}$(53)<br>[$4.98x10^{-8}$ – $3.84x10^{-7}$]<br>{HZY3019/3020}	0.7
*rad59Δ***	$5.85x10^{-9}$ (2.6)<br>[$0.57x10^{-9}$ – $24.7x10^{-9}$]	$6.94x10^{-8}$ (31)<br>[$5.17x10^{-8}$ – $9.12x10^{-8}$]	12
rad59Δ mms21-CH	$3.66x10^{-7}$ (161)<br>[$1.56x10^{-7}$ – 8.82 $x10^{-7}$]<br>{HZY3362/3363}	$2.71x10^{-6}$ (1194)<br>[$1.16x10^{-6}$ – $7.30x10^{-6}$]<br>{HZY3166/3167}	7.4
rad51Δ	$6.19x10^{-9}$ (2.7)<br>[$2.17x10^{-9}$ – $2.99x10^{-8}$]<br>{HZY3264}	$2.31x10^{-8}$ (10)**<br>[$1.20x10^{-8}$ – $4.35x10^{-8}$]	3.7
rad51Δ mms21-CH	$2.17x10^{-8}$ (9.6)<br>[$4.06x10^{-9}$ – $4.90x10^{-8}$]<br>{HZY3265/3266}	1.91 $x10^{-7}$ (84)<br>[$1.02x10^{-7}$ – $5.00x10^{-7}$]<br>{HZY3257/3258,JLY380}	8.8
rad55Δ	$5.62x10^{-8}$ (25)<br>[$4.02x10^{-8}$ – $6.46x10^{-8}$]<br>{HZY3369}	$2.95x10^{-8}$ (13)<br>[$4.28x10^{-9}$ – $2.29x10^{-7}$]<br>{HZY3021/3368}	0.5
rad55Δ mms21-CH	$2.23x10^{-8}$ (9.8)<br>[$1.15x10^{-8}$ – $7.86x10^{-8}$]<br>{HZY3245/3246}	$1.41x10^{-7}$ (62)<br>[$6.74x10^{-8}$ – $2.23x10^{-7}$]<br>{HZY3025/3026}	6.3
rad54Δ	1.4 x 10^{-8} (6.2)<br>[$4.17x10^{-9}$ – $1.74x10^{-8}$]<br>{HZY3269/3270,JLY406}	$2.16x10^{-8}$ (9.5)<br>[$1.11x10^{-8}$ – $1.03x10^{-7}$]<br>{HZY3299/3306}	6.1
rad54Δ mms21-CH	$1.18x10^{-7}$ (52)<br>[$4.27x10^{-8}$ – $1.15x10^{-6}$]<br>{HZY3271/3272}	$3.0x10^{-8}$ (13)<br>[$8.71x10^{-9}$ – $9.26x10^{-8}$]<br>{HZY3300/3301}	0.3
rdh54Δ	$3.72x10^{-9}$ (1.6)<br>[$2.26x10^{-9}$ – $7.75x10^{-9}$]<br>{HZY3277,JLY382}	$7.85x10^{-8}$ (35)<br>[$4.36x10^{-8}$ – $1.27x10^{-7}$]<br>{HZY3274/JLY442}	21
rdh54Δ mms21-CH	$5.05x10^{-8}$ (22)<br>[$7.32x10^{-8}$ – $1.88x10^{-8}$]<br>{HZY3280/3281}	$4.26x10^{-6}$ (1877)<br>[$2.98x10^{-6}$ – $6.56x10^{-6}$]<br>{HZY3275/3276,JLY444}	84
*mre11Δ***	$5.75x10^{-7}$ (253)<br>[$2.82x10^{-7}$ – $19.2x10^{-7}$]	$1.52x10^{-6}$ (670)<br>[$0.95x10^{-6}$ – $2.77x10^{-6}$]	2.6
mre11Δ mms21-CH	$1.39x10^{-6}$ (612)<br>[$1.13x10^{-6}$ – $1.58x10^{-6}$]	$6.66x10^{-6}$ (2934)<br>[$3.28x10^{-6}$ – $1.49x10^{-5}$]	4.8

	{HZY2480/2481}	{HZY2476/2477/2516}	
Genotype	uGCR rate*	dGCR rate*	Ratio #
mre11-H125N	$2.78x10^{-7}$ (122)<br>[$1.80x10^{-7}$ – $4.01x10^{-7}$]<br>{HZY2771/2772}	$6.23x10^{-7}$ (274)<br>[$5.24x10^{-7}$ – $9.75x10^{-7}$]<br>{HZY2763/2764}	2.2
mre11-H125N mms21-CH	$6.35x10^{-7}$ (280)<br>[$2.73x10^{-7}$ – $1.43x10^{-6}$]<br>{JLY929/930}	$4.17x10^{-6}$ (1837)<br>[$2.86x10^{-6}$ – $5.28x10^{-6}$]<br>{JLY973/974/975}	6.6
*sae2Δ***	$4.23x10^{-8}$ (19)<br>[$2.92x10^{-8}$ – $5.32x10^{-8}$]	$1.65x10^{-7}$ (73)<br>[$1.09x10^{-7}$ – $2.09x10^{-7}$]	3.9
sae2Δ mms21-CH	$6.61x10^{-7}$ (291)<br>[$2.66x10^{-7}$ – $1.03x10^{-6}$]<br>{HZY2599/2600}	$6.03x10^{-6}$ (2656)<br>[$3.33x10^{-6}$ – $8.81x10^{-6}$]<br>{HZY2591/2592}	9.1

* Rate of accumulating Can 5-FOA progeny. Number in the parenthesis is the fold increase relative to wild-type uGCR strain.
** Rates taken from (Putnam et al., 2009; 2010; Albuquerque et al., 2013).
# The dGCR rate divided by the uGCR rate.
Square brackets represent the upper and lower limits of the 95% confidence intervals of the median.
Strains used to calculate the GCR rate from this study are as indicated in curly braces.
