# Supplementary material for: SUMO E3 ligase Mms21 prevents spontaneous DNA damage induced genome rearrangements
Source: PLoS Genet. 2018 Mar 5;14(3):e1007250. doi: 10.1371/journal.pgen.1007250 (PMC5860785; doi:10.1371/journal.pgen.1007250)
Supplement: S2 Table — (DOCX) [file pgen.1007250.s019.docx]

**S2 Table. Summary of Can^R^ 5FOA^R^ isolates from the uGCR assay.**

| **Isolate** | **Relevant Genotype** | **ChrV size by CHEF gel^*^** | **WGS Sample Name** | **Hygromycin Resistance** | **Type^**^** |
| --- | --- | --- | --- | --- | --- |
| PGSP3251 | wild-type | smaller | - | - | *de novo* telomere addition |
| PGSP3252 | wild-type | smaller | - | - | *de novo* telomere addition |
| PGSP3254 | wild-type | smaller | - | - | *de novo* telomere addition |
| PGSP3255 | wild-type | similar | - | + | interstitial deletion |
| PGSP3256 | wild-type | smaller | - | - | *de novo* telomere addition |
| PGSP3257 | wild-type | larger | bzg001 | - | hairpin-mediated inverted duplication + t(V L; V R) |
| PGSP3258 | wild-type | smaller | - | - | *de novo* telomere addition |
| PGSP3259 | wild-type | smaller | - | - | *de novo* telomere addition |
| PGSP3260 | wild-type | smaller | - | - | *de novo* telomere addition |
| PGSP3261 | wild-type | smaller | - | - | *de novo* telomere addition |
| PGSP3262 | wild-type | smaller | - | - | *de novo* telomere addition |
| PGSP3263 | wild-type | smaller | bzg002 | - | interstitial deletion |
| PGSP4078 | *mre11* | smaller | - | - | *de novo* telomere addition |
| PGSP4079 | *mre11* | larger | bzg003 | - | microhomology-mediated t(V L;XII R) |
| PGSP4080 | *mre11* | smaller | - | - | *de novo* telomere addition |
| PGSP4081 | *mre11* | smaller | - | - | *de novo* telomere addition |
| PGSP4082 | *mre11* | larger | bzg004 | + | hairpin-mediated inverted duplication + *URA3/ura3-52* translocation |
| PGSP4083 | *mre11* | smaller | - | - | *de novo* telomere addition |
| PGSP4084 | *mre11* | larger | bzg005 | - | hairpin-mediated inverted duplication  + t(V L;XII R) |
| PGSP4085 | *mre11* | larger | bzg006 | + | hairpin-mediated inverted duplication + *URA3/ura3-52* translocation |
| PGSP4086 | *mre11* | larger | bzg007 | + | hairpin-mediated inverted duplication + *URA3/ura3-52* translocation |
| PGSP4087 | *mre11* | similar | - | - | *de novo* telomere addition |
| PGSP4088 | *mre11* | similar | - | + | interstitial deletion |
| PGSP4089 | *mre11* | larger | bzg008 | - | microhomology-mediated t(V L;XII R) |
| PGSP4090 | *mre11* | larger | bzg009 | - | microhomology-mediated t(VL;XII R) |
| JLM001 | *mms21-CH* | smaller | - | - | *de novo* telomere addition |
| JLM002 | *mms21-CH* | larger | bzg010 | - | microhomology-mediated t(V;XII) |
| JLM003 | *mms21-CH* | smaller | - | - | *de novo* telomere addition |
| JLM004 | *mms21-CH* | larger | bzg011 | - | homology-mediated t(V;XIV) |
| JLM006 | *mms21-CH* | larger | bzg012 | - | microhomology-mediated t(V;XI) |
| JLM007 | *mms21-CH* | smaller | - | - | *de novo* telomere addition |
| JLM008 | *mms21-CH* | smaller | - | - | *de novo* telomere addition |
| JLM009 | *mms21-CH* | smaller | - | - | *de novo* telomere addition |
| JLM011 | *mms21-CH* | smaller | - | - | *de novo* telomere addition |
| JLM012 | *mms21-CH* | smaller | - | - | *de novo* telomere addition |
| JLM013 | *mms21-CH* | smaller | - | - | *de novo* telomere addition |
| JLM104 | *mms21-CH mre11* | similar | - | - | *de novo* telomere addition |
| JLM105 | *mms21-CH mre11* | larger | bzg013 | + | hairpin-mediated inverted duplication + *URA3/ura3-52* translocation  + chrVIII disomy |
| JLM106 | *mms21-CH mre11* | smaller | - | - | *de novo* telomere addition |
| JLM107 | *mms21-CH mre11* | larger | bzg014 | + | hairpin-mediated inverted duplication  + *URA3/ura3-52* translocation  + chrVIII disomy |
| JLM136 | *mms21-CH mre11* | larger | bzg016 | - | hairpin-mediated inverted duplication  + t(V L;XII R)  + chrVIII disomy |
| JLM137 | *mms21-CH mre11* | larger | bzg015 | - | hairpin-mediated inverted duplication  + translocation involving chr XII R and XIV R |
| JLM138 | *mms21-CH mre11* | larger | bzg017 | - | hairpin-mediated inverted duplication  + t(V L;XII R)  + chrI disomy |
| JLM139 | *mms21-CH mre11* | larger | bzg018 | - | hairpin-mediated inverted duplication  + t(V L;XII R)  + chrVIII disomy |
| JLM140 | *mms21-CH mre11* | larger | bzg019 | - | hairpin-mediated inverted duplication  + t(V L;XII R)  + chrVIII disomy |
| JLM141 | *mms21-CH mre11* | larger | bzg020 | - | hairpin-mediated inverted duplication  + t(V L;XII R)  + chrVIII disomy |
| JLM142 | *mms21-CH mre11* | larger | bzg021 | - | hairpin-mediated inverted duplication  + t(V L;XV L)  + chrVIII disomy |
| JLM143 | *mms21-CH mre11* | larger | bzg022 | - | hairpin-mediated inverted duplication  + t(V L;XII R)  + chrVIII disomy |
| JLM52 | *mms21-CH mre11-H125N* | larger | bzg030 | - | microhomology-mediated t(V L;XII R) |
| JLM53 | *mms21-CH mre11-H125N* | larger | bzg031 | - | microhomology-mediated t(V L;XII R; VII R) |
| JLM54 | *mms21-CH mre11-H125N* | similar | - | - | *de novo* telomere addition |
| JLM55 | *mms21-CH mre11-H125N* | larger | bzg032 | - | microhomology-mediated t(V L;XII R) |
| JLM56 | *mms21-CH mre11-H125N* | larger | bzg033 | - | hairpin-mediated inverted duplication + t(V L;XV R) |
| JLM57 | *mms21-CH mre11-H125N* | similar | - | - | *de novo* telomere addition |
| JLM58 | *mms21-CH mre11-H125N* | larger | bzg034 | - | hairpin-mediated inverted duplication + t(V L;XII R) |
| JLM59 | *mms21-CH mre11-H125N* | larger | bzg035 | - | hairpin-mediated inverted duplication + t(V L;XII R) |
| JLM60 | *mms21-CH mre11-H125N* | larger | bzg036 | - | microhomology-mediated t(V L;XV R) |
| JLM61 | *mms21-CH mre11-H125N* | larger | bzg037 | - | microhomology-mediated t(V L;XII R) |
| JLM62 | *mms21-CH mre11-H125N* | larger | bzg038 | - | microhomology-mediated t(V L;XII R) |
| JLM63 | *mms21-CH mre11-H125N* | larger | bzg039 | - | microhomology-mediated t(V L;V R;XIII L) |
| JLM64 | *mms21-CH mre11-H125N* | larger | bzg040 | - | hairpin-mediated inverted duplication + t(V L;X R) |
| JLM65 | *mms21-CH mre11-H125N* | larger | bzg041 | - | hairpin-mediated inverted duplication + t(V L;IV R) |
| mre11-H125N#1 | *mre11-H125N* | smaller | - | + | interstitial deletion |
| mre11-H125N #2 | *mre11-H125N* | similar | - | + | interstitial deletion |
| mre11-H125N #3 | *mre11-H125N* | smaller | - | - | *de novo* telomere addition |
| mre11-H125N #4 | *mre11-H125N* | similar | - | + | interstitial deletion |
| mre11-H125N #5 | *mre11-H125N* | similar | - | + | interstitial deletion |
| mre11-H125N #6 | *mre11-H125N* | smaller | - | - | *de novo* telomere addition |
| mre11-H125N #7 | *mre11-H125N* | similar | - | + | interstitial deletion |
| mre11-H125N #8 | *mre11-H125N* | larger | bzg042 | - | hairpin-mediated inverted duplication + t(V L;XII R) |
| mre11-H125N #9 | *mre11-H125N* | larger | bzg043 | + | hairpin-mediated inverted duplication + *URA3/ura3-52* translocation |
| mre11-H125N #10 | *mre11-H125N* | larger | bzg044 | + | hairpin-mediated inverted duplication + *URA3/ura3-52* translocation |
| mre11-H125N #11 | *mre11-H125N* | larger | bzg045 | + | hairpin-mediated inverted duplication + *URA3/ura3-52* translocation |
| mre11-H125N #12 | *mre11-H125N* | larger | bzg046 | - | hairpin-mediated inverted duplication + t(V L;X R) |
| mre11-H125N #13 | *mre11-H125N* | larger | bzg047 | - | hairpin-mediated inverted duplication + t(V L;IV R) |

^*^Size reported relative to the size of chromosome V in the parental strain lacking a GCR.

^**^Isolates were assigned to the *de novo* telomere addition in the absence of other information if they had (1) a chromosome V that was smaller than wild-type and (2) loss of the telomeric hygromycin resistance marker *hph*. Isolates were assigned to the interstitial deletion in the absence of other information if they had a (1) a chromosome V that was similar to or smaller than wild-type and (2) retention of *hph*. This analysis cannot rule out the possibility that some isolates may have an interstitial deletion or translocation causing both a smaller size and loss of the *hph* marker.
