# Supplementary material for: SUMO E3 ligase Mms21 prevents spontaneous DNA damage induced genome rearrangements
Source: PLoS Genet. 2018 Mar 5;14(3):e1007250. doi: 10.1371/journal.pgen.1007250 (PMC5860785; doi:10.1371/journal.pgen.1007250)
Supplement: S3 Table — (DOCX) [file pgen.1007250.s020.docx]

**S3 Table. Statistics for Whole Genome Sequencing results.**

| **Sample**  **(Relevant Gentoype)** | **No. Read Pairs^*^** | **% Read 1 Mapped** | **% Read 2 Mapped** | **No. Uniquely Mapping Read Pairs** | **Median Intra-Read Pair Distance (bp)** | **Median Read Depth^**^** |
| --- | --- | --- | --- | --- | --- | --- |
| bzg001  (wild-type) | 40,786,020  (44,192,529) | 99.43% | 95.58% | 34,777,694 | 395 | 289 |
| bzg002  (wild-type) | 41,369,356  (44,143,194) | 99.40% | 96.21% | 35,324,321 | 373 | 275 |
| bzg003  (*mre11*) | 19,703,687  (21,311,670) | 98.27% | 94.47% | 16,092,716 | 393 | 124 |
| bzg004  (*mre11*) | 37,498,294  (41,454,254) | 99.14% | 96.64% | 32,165,819 | 335 | 284 |
| bzg005  (*mre11*) | 35,134,728  (38,732,783) | 99.19% | 96.98% | 30,248,955 | 322 | 258 |
| bzg006  (*mre11*) | 30,964,848  (34,968,820) | 99.06% | 96.27% | 26,054,529 | 312 | 211 |
| bzg007  (*mre11*) | 13,264,577  (13,860,940) | 99.32% | 96.32% | 11,121,693 | 349 | 95 |
| bzg008  (*mre11*) | 23,485,318  (26,020,641) | 99.37% | 96.70% | 19,659,711 | 328 | 168 |
| bzg009  (*mre11*) | 25,275,393  (26,640,602) | 98.87% | 95.62% | 21,563,996 | 362 | 195 |
| bzg010  (*mms21-CH*) | 26,471,341  (27,583,800) | 99.29% | 95.77% | 22,795,312 | 369 | 205 |
| bzg011  (*mms21-CH*) | 21,973,676  (23,403,227) | 99.29% | 95.67% | 18,417,083 | 391 | 162 |
| bzg012  (*mms21-CH*) | 11,743,627  (12,250,798) | 99.23% | 95.50% | 9,767,111 | 398 | 83 |
| bzg013  (*mms21-CH mre11*) | 22,926,548  (25,320,705) | 99.29% | 94.62% | 19,101,999 | 344 | 160 |
| bzg014  (*mms21-CH mre11*) | 19,415,905  (21,366,096) | 99.28% | 94.68% | 16,056,400 | 344 | 133 |
| bzg015  (*mms21-CH mre11*) | 21,443,423  (23,429,396) | 98.61% | 94.09% | 17,787,190 | 338 | 139 |
| bzg016  (*mms21-CH mre11*) | 25,074,767  (28,588,213) | 99.16% | 95.05% | 20,860,751 | 325 | 169 |
| bzg017  (*mms21-CH mre11*) | 30,089,574  (32,393,510) | 99.27% | 95.43% | 25,602,695 | 316 | 215 |
| bzg018  (*mms21-CH mre11*) | 21,587,157  (23,584,688) | 99.35% | 94.95% | 17,941,199 | 337 | 150 |
| bzg019  (*mms21-CH mre11*) | 15,662,127  (16,728,362) | 98.61% | 94.29% | 12,899,338 | 329 | 106 |
| bzg020  (*mms21-CH mre11*) | 27,202.784  (29,542,738) | 99.36% | 94.97% | 23,085,997 | 336 | 192 |
| bzg021  (*mms21-CH mre11*) | 19,095,179  (20,377,752) | 99.01% | 94.01% | 15,799,313 | 336 | 137 |
| bzg022  (*mms21-CH mre11*) | 19,727,697  (21,391,013) | 99.24% | 94.31% | 16,346,267 | 331 | 132 |
| bzg030  (*mms21-CH mre11-H125N*) | 27,887,528  (29,565,440) | 99.57% | 96.82% | 24,128,142 | 303 | 212 |
| bzg031  (*mms21-CH mre11-H125N*) | 28,845,369  (31,659,248) | 99.55% | 96.63% | 24,960,576 | 316 | 210 |
| bzg032  (*mms21-CH mre11-H125N*) | 22,649,136  (24,421,773) | 99.50% | 96.18% | 19,393,429 | 340 | 162 |
| bzg033  (*mms21-CH mre11-H125N*) | 21,389,337  (23,010,007) | 99.33% | 95.52% | 18,101,467 | 347 | 157 |
| bzg034  (*mms21-CH mre11-H125N*) | 22,114,702  (23,873,469) | 99.43% | 95.56% | 18,723,887 | 352 | 163 |
| bzg035  (*mms21-CH mre11-H125N*) | 24,532,594  (26,130,327) | 99.54% | 96.31% | 20,946,390 | 322 | 181 |
| bzg036  (*mms21-CH mre11-H125N*) | 19,693,201  (20,938,775) | 99.45% | 96.13% | 16,810,990 | 325 | 141 |
| bzg037  (*mms21-CH mre11-H125N*) | 26,564,952  (28,589,410) | 99.45% | 95.50% | 22,502,926 | 350 | 197 |
| bzg038  (*mms21-CH mre11-H125N*) | 17,932,900  (19,028,331) | 99.59% | 95.59% | 15,144,373 | 357 | 130 |
| bzg039  (*mms21-CH mre11-H125N*) | 23,389,211  (24,936,369) | 99.40% | 96.07% | 19,836,895 | 339 | 176 |
| bzg040  (*mms21-CH mre11-H125N*) | 25,725,667  (28,154,376) | 97.27% | 94.29% | 21,109,605 | 332 | 181 |
| bzg041  (*mms21-CH mre11-H125N*) | 34,703,539  (37,573,272) | 97.83% | 94.49% | 28,780,405 | 326 | 261 |
| bzg042  (*mre11-H125N*) | 19,373,314  (21,065,622) | 98.54% | 95.08% | 15,924,703 | 326 | 126 |
| bzg043  (*mre11-H125N*) | 26,786,386  (31,820,709) | 99.55% | 95.60% | 22,526,739 | 358 | 201 |
| bzg044  (*mre11-H125N*) | 29,044,961  (38,495,985) | 99.62% | 94.91% | 24,165,775 | 384 | 221 |
| bzg045  (*mre11-H125N*) | 22,752,374  (25,051,806) | 99.47% | 94.82% | 19,066,376 | 374 | 150 |
| bzg046  (*mre11-H125N*) | 23,275,209  (28,917,458) | 99.49% | 94.19% | 19,166,089 | 402 | 146 |
| bzg047  (*mre11-H125N*) | 24,726,817  (27,430,629) | 99.58% | 95.84% | 20,897,867 | 351 | 165 |

^*^Numbers in parentheses indicate the number of read pairs prior to culling PCR duplicates. Reads were 50 bases long.

^**^Median read depth is the median number of times that each base in uniquely mapping regions of the nuclear genome was present within a read.
