# Supplementary material for: SUMO E3 ligase Mms21 prevents spontaneous DNA damage induced genome rearrangements
Source: PLoS Genet. 2018 Mar 5;14(3):e1007250. doi: 10.1371/journal.pgen.1007250 (PMC5860785; doi:10.1371/journal.pgen.1007250)
Supplement: S4 Table — (PDF) [file pgen.1007250.s021.pdf]

**S4 Table.** Number of junction-defining read pairs and junction-sequencing reads for Can$^R$ 5FOA$^R$ isolates from the uGCR assay with sequenced genomes.

| **Sample (Relevant Genotype)** | **GCR Junction Description** | **GCR Junction Evidence[1]** |
|---|---|---|
| bzg001 (wild-type) | Hairpin-mediated inverted duplication at chrV L (~35,800) | Copy number (Fig. S3)<br>Discordant read pairs (172 read pairs)<br>Junction sequence (54 reads; Fig. S10a) |
| | Homology translocation between chrV L *YELCdelta4* and chrV R *YERCTy1-2* | Copy number (Fig. S3) |
| bzg002 (wild-type) | Interstitial deletion | Copy number (Fig. S3)<br>Discordant read pairs (1443 read pairs)<br>Junction sequence (229 reads; Fig. S3) |
| bzg003 (*mre11*) | Microhomology translocation between chrV L (~29,000) and chrXII R rDNA repeats | Copy number (Fig. S5)<br>Junction sequence (Fig. S5) |
| bzg004 (*mre11*) | Hairpin-mediated inverted duplication at chrV L (~42,400) | Copy number (Fig. S5)<br>Discordant read pairs (309 read pairs)<br>Junction sequence (228 reads; Fig. S10b) |
| | Homology translocation between chrV L *ura3-52* and chrV L *URA3* in *CAN1-URA3* cassette | Copy number (Fig. S5) |
| bzg005 (*mre11*) | Hairpin-mediated inverted duplication at chrV L *can1::hisG* | Copy number (Fig. S5)<br>Junction sequence (Fig. S10c) |
| | Homology translocation between chrV L *ura3-52* and chrXII R *YLRWTy1-3* | Copy number (Fig. S5) |
| bzg006 (*mre11*) | Hairpin-mediated inverted duplication at chrV L *can1::hisG* | Copy number (Fig. S5)<br>Discordant read pairs (480 read pairs)<br>Junction sequence (22 reads; Fig. S10d) |
| | Homology translocation between chrV L *ura3-52* and chrV L *URA3* in *CAN1-URA3* cassette | Copy number (Fig. S5) |
| bzg007 (*mre11*) | Hairpin-mediated inverted duplication at chrV L *can1::hisG* | Copy number (Fig. S5)<br>Discordant read pairs (5 read pairs)<br>Junction sequence (8 reads; Fig. S10e) |
| | Homology translocation between chrV L *ura3-52* and chrV L *URA3* in *CAN1-URA3* cassette | Copy number (Fig. S5) |

| Sample (Relevant Genotype) | GCR Junction Description | GCR Junction Evidence[1] |
|---|---|---|
| bzg008 (*mre11*) | Microhomology translocation between chrV L and chrXII R rDNA | Copy number (Fig. S5)<br>Junction sequence (43 reads; Fig. S5) |
| bzg009 (*mre11*) | Microhomology translocation between chrV L and chrXII R rDNA | Copy number (Fig. S5)<br>Junction sequence (Fig. S5) |
| bzg010 (*mms21-CH*) | Non-homology translocation between chrV L and chrXII R rDNA | Copy number (Fig. S5)<br>Junction sequence (101 reads; Fig. S5) |
| bzg011 (*mms21-CH*) | Homology translocation between chrV L *CAN1* and chrXIV *LYP1* | Copy number (Fig. S5)<br>Discordant read pairs (630 read pairs)<br>Junction sequence (44 reads; Fig. S4) |
| bzg012 (*mms21-CH*) | Microhomology translocation between chrV L and chrXI | Copy number (Fig. S5)<br>Discordant read pairs (249 read pairs)<br>Junction sequence (33 reads; Fig. S4) |
| bzg013 (*mms21-CH mre11*) | Hairpin-mediated inverted duplication at chrV L (~42,200) | Copy number (Fig. S7)<br>Discordant read pairs (10 read pairs)<br>Junction sequence (23 reads; Fig. S10f) |
| | Homology translocation between chrV L *ura3-52* and chrV L *URA3* in *CAN1-URA3* cassette | Copy number (Fig. S7) |
| bzg014 (*mms21-CH mre11*) | Hairpin-mediated inverted duplication at chrV L (~27,000) | Copy number (Fig. S7)<br>Discordant read pairs (19 read pairs)<br>Junction sequence (34 reads; Fig. S10g) |
| | Homology-mediated translocation between chrV L *YELWdelta6* and the second delta sequence in chrV L *ura3-52* | Copy number (Fig. S7)<br>Junction sequence (Fig. S13) |
| | Homology translocation between chrV L *ura3-52* and chrV L *URA3* in *CAN1-URA3* cassette | Copy number (Fig. S7) |
| | chrVIII disomy | Copy depth histogram (Fig. S16) |
| bzg015 (*mms21-CH mre11*) | Hairpin-mediated inverted duplication at chrV L (~25,800) copying to chrV L *ura3-52* | Copy number (Fig. S7)<br>Discordant read pairs (68 read pairs)<br>Junction sequence (116 reads; Fig. S10h) |
| | Duplication from chrXII R *YLRWdelta4* to chrXII R *TEL12R*; unclear how this duplication relates to either chrV hairpin or duplication on chrXIV R. Also well-supported but unexpected junction in rDNA | Copy number (Fig. S7) |

| Sample (Relevant Genotype) | GCR Junction Description | GCR Junction Evidence[1] |
|---|---|---|
| | repeats chrXII:459765..460321. | |
| | Duplication from chrXIV R *YPRWTy1-3/YPRCTy1-4* to chrXIV *TEL16R*; unclear how this duplication relates to either chrV hairpin or duplication on chrXII R. | Copy number (Fig. S7) |
| bzg016 (*mms21-CH mre11*) | Hairpin-mediated inverted duplication at chrV L *can1::hisG* | Copy number (Fig. S7)<br>Discordant read pairs (8 read pairs)<br>Junction sequence (247 reads; Fig. S10i) |
| | Homology-mediated translocation between chrXII R "*YLRWTy1-4*", a Ty adjacent to *YLRCdelta21* not present in the reference sequence (Fig. S13) | Copy number (Fig. S7) |
| | chrVIII disomy | Copy depth histogram (Fig. S16) |
| bzg017 (*mms21-CH mre11*) | Hairpin-mediated inverted duplication at chrV L (~30,900) | Copy number (Fig. S7)<br>Discordant read pairs (29 read pairs)<br>Junction sequence (188 read pairs; Fig S10j) |
| | Translocation between chrV L *YELWdelta6* and chrXII R *YLRWdelta6* | Copy number (Fig. S7)<br>Discordant read pairs (280 read pairs)<br>Junction sequence (Fig. S11) |
| bzg018 (*mms21-CH mre11*) | Hairpin-mediated inverted duplication at chrV L *can1::hisG* | Copy number (Fig. S7) |
| | Homology-mediated translocation between chrXII R "*YLRWTy1-4*", a Ty adjacent to *YLRCdelta21* not present in the reference sequence (Fig. S13) | Copy number (Fig. S7) |
| | chrVIII disomy | Copy depth histogram (Fig. S16) |
| bzg019 (*mms21-CH mre11*) | Hairpin-mediated inverted duplication at chrV L *can1::hisG* | Copy number (Fig. S7) |
| | Homology translocation between chrV L *ura3-52* and chrXII R *YLRWTy1-3* | Copy number (Fig. S7) |
| | chrVIII disomy | Copy depth histogram (Fig. S16) |
| bzg020 (*mms21-CH mre11*) | Hairpin-mediated inverted duplication at chrV L (~30,000) | Copy number (Fig. S7)<br>Discordant read pairs (24 read pairs)<br>Junction sequence (30 reads; Fig. S10k) |
| | Homology translocation between chrV L *ura3-52* and chrXII R *YLRWTy1-3* | Copy number (Fig. S7) |

| Sample<br>(Relevant Genotype) | GCR Junction Description | GCR Junction Evidence[1] |
|---|---|---|
| | chrVIII disomy | Copy depth histogram (Fig. S16) |
| bzg021<br>(*mms21-CH mre11*) | Hairpin-mediated inverted duplication at chrV L (~26,500) | Copy number (Fig. S7)<br>Discordant read pairs (11 read pairs)<br>Junction sequence (35 reads; Fig. S10l) |
| | Homology translocation between chrV L *PAU2* and chrXV L *PAU20* | Copy number (Fig. S7)<br>Discordant read pairs (114 read pairs)<br>Junction sequence (Fig. S12) |
| | chrVIII disomy | Copy depth histogram (Fig. S16) |
| bzg022<br>(*mms21-CH mre11*) | Hairpin-mediated inverted duplication at chrV L (~28,900) | Copy number (Fig. S7)<br>Discordant read pairs (55 read pairs)<br>Junction sequence (53 reads; Fig. S10m) |
| | Homology translocation between chrV L *ura3-52* and chrXII R *YLRWTy1-3* | Copy number (Fig. S7) |
| | chrVIII disomy | Copy depth histogram (Fig. S15) |
| bzg030<br>(*mms21-CH mre11-H125N*) | Microhomology translocation between chrV L and chrXII R rDNA | Copy number (Fig. S8)<br>Junction sequence (56 reads; Fig. S8) |
| bzg031<br>(*mms21-CH mre11-H125N*) | Microhomology translocation between chrV L and chrXII R | Copy number (Fig. S8)<br>Discordant read pairs (438 read pairs)<br>Junction sequence (123 reads; Fig. S8) |
| | Homology translocation between chrXII R *YLRWTy2-1* and chrVII R *YGRCTy1-2* or *YGRCTy2-1* | Copy number (Fig. S8) |
| bzg032<br>(*mms21-CH mre11-H125N*) | Non-homology translocation between chrV L and chrXII R | Copy number (Fig. S8)<br>Discordant read pairs (467 read pairs)<br>Junction sequence (95 reads; Fig. S8) |
| bzg033<br>(*mms21-CH mre11-H125N*) | Hairpin-mediated inverted duplication at chrV L *can1::hisG* | Copy number (Fig. S8)<br>Junction sequence (Fig. S10c) |
| | Homology translocation between chrV L *ura3-52* and chrXV R *YORWTy2-2* | Copy number (Fig. S8) |
| bzg034<br>(*mms21-CH* | Hairpin-mediated inverted duplication at chrV L (~27,000) | Copy number (Fig. S8)<br>Discordant read pairs (45 read pairs) |

| Sample (Relevant Genotype) | GCR Junction Description | GCR Junction Evidence[1] |
|---|---|---|
| *mre11-H125N*) | | Junction sequence (42 reads; Fig. S10n) |
| | Homology translocation between chrV L *ura3-52* and chrXII R *YLRWTy1-2* | Copy number (Fig. S8) |
| bzg035 (*mms21-CH mre11-H125N*) | Hairpin-mediated inverted duplication at chrV L (~33,100) | Copy number (Fig. S8)<br>Junction sequence (53 reads; Fig. S10o) |
| | Microhomology translocation from chrV L to chrXII R in *YLRWTy1-3* | Copy number (Fig. S8)<br>Junction sequence (50 reads; Fig. S8) |
| bzg036 (*mms21-CH mre11-H125N*) | Non-homology translocation between chrV L and chrXV R | Copy number (Fig. S8)<br>Discordant read pairs (310 read pairs)<br>Junction sequence (53 reads; Fig. S8) |
| bzg037 (*mms21-CH mre11-H125N*) | Microhomology translocation between chrV L and chrXII R rDNA | Copy number (Fig. S8)<br>Junction sequence (14 reads; Fig. S8) |
| bzg038 (*mms21-CH mre11-H125N*) | Microhomology translocation between chrV L and chrXII R rDNA | Copy number (Fig. S8)<br>Junction sequence (13 reads; Fig. S8) |
| bzg039 (*mms21-CH mre11-H125N*) | Microhomology translocation between chrV L and chrV R | Copy number (Fig. S8)<br>Discordant read pairs (584 read pairs)<br>Junction sequence (115 reads; Fig. S8) |
| | Homology translocation between chrV R *YERCTy1-2* and chrXIII L *YMLWTy1-1* | Copy number (Fig. S8) |
| bzg040 (*mms21-CH mre11-H125N*) | Hairpin-mediated inverted duplication at chrV L (~29,500) | Copy number (Fig. S8)<br>Discordant read pairs (119 read pairs)<br>Junction sequence (52 reads; Fig. S10p) |
| | Homology translocation between chrV L *ura3-52* and chrX R *YJRWTy1-1* or *YJRWTy1-2* | Copy number (Fig. S8) |
| bzg041 (*mms21-CH mre11-H125N*) | Hairpin-mediated inverted duplication at chrV L *can1::hisG* | Copy number (Fig. S8)<br>Junction sequence (Fig. S10c) |
| | Microhomology translocation from chrV L to chrIV R | Copy number (Fig. S8)<br>Discordant read pairs (455 read pairs)<br>Junction sequence (100 reads; Fig. S8) |

| **Sample (Relevant Genotype)** | **GCR Junction Description** | **GCR Junction Evidence**[1] |
|---|---|---|
| bzg042 (*mre11-H125N*) | Hairpin-mediated inverted duplication at chrV L *can1::hisG* | Copy number (Fig. S6)<br>Junction sequence (Fig. S10q) |
| | Homology-mediated translocation between chrXII R "*YLRWTy1-4*", a Ty adjacent to *YLRCdelta21* not present in the reference sequence (Fig. S13) | Copy number (Fig. S6) |
| bzg043 (*mre11-H125N*) | Hairpin-mediated inverted duplication at chrV L *can1::hisG* | Copy number (Fig. S6)<br>Junction sequence (Fig. S10c) |
| | Homology translocation between chrV L *ura3-52* and chrV L *URA3* in *CAN1-URA3* cassette | Copy number (Fig. S6) |
| bzg044 (*mre11-H125N*) | Hairpin-mediated inverted duplication at chrV L *can1::hisG* | Copy number (Fig. S6)<br>Junction sequence (Fig. S10c) |
| | Homology translocation between chrV L *ura3-52* and chrV L *URA3* in *CAN1-URA3* cassette | Copy number (Fig. S6) |
| bzg045 (*mre11-H125N*) | Hairpin-mediated inverted duplication at chrV L *can1::hisG* | Copy number (Fig. S6)<br>Junction sequence (Fig. S10c) |
| | Homology translocation between chrV L *ura3-52* and chrV L *URA3* in *CAN1-URA3* cassette | Copy number (Fig. S6) |
| bzg046 (*mre11-H125N*) | Hairpin-mediated inverted duplication at chrV L *can1::hisG* | Copy number (Fig. S6)<br>Junction sequence (Fig. S10c) |
| | Homology translocation between chrV L *ura3-52* and chrX R *YJRWTy1-1* or *YJRWTy1-2* | Copy number (Fig. S6) |
| bzg047 (*mre11-H125N*) | Hairpin-mediated inverted duplication at chrV L (~26,500) | Copy number (Fig. S6)<br>Discordant read pairs (27 read pairs)<br>Junction sequence (40 reads; Fig. S10r) |
| | Homology translocation between chrV L *ura3-52* and chrIV R *YDRWTy1-4* | Copy number (Fig. S6) |

[1]Evidence supporting GCR-related junctions can be derived from whole genome sequencing data in several ways. Frequently, multiple lines of evidence support many junctions. Copy number changes, from normalized read depth plots of uniquely mapping regions of the genome, can suggest possible rearrangements, particularly those involving HR between repetitive elements. Discordant read pairs are read pairs where two uniquely mapping reads map to regions consistent with the GCR, but not with reference chromosome; note that the number of discordant read pairs is influenced by whether the rearrangement in within or adjacent to non-uniquely mapping regions of the genome.

Junction sequences can be determined in two ways, depending on the junction. For junctions that generate a novel sequence, such as a deletion or hairpin-mediated inversion, the sequence can be derived from read pairs adjacent to junction-defining discordant read pairs in which one read does not map to the reference and potentially sequences the novel junction. In these cases, the numbers of reads that align to define the novel junction sequence are reported. For junctions mediated by homologies, such as tRNA genes or Ty-related sequences, sequences can be determined by aligning reads that are linked to uniquely mapping regions adjacent to the homologies involved in the junction.
