# Supplementary material for: SUMO E3 ligase Mms21 prevents spontaneous DNA damage induced genome rearrangements
Source: PLoS Genet. 2018 Mar 5;14(3):e1007250. doi: 10.1371/journal.pgen.1007250 (PMC5860785; doi:10.1371/journal.pgen.1007250)
Supplement: S5 Table — (PDF) [file pgen.1007250.s022.pdf]

S5 Table. Fluctuation results of double mutants containing a null mutation of genes in the BIR pathway and *mms21-CH* or *sgs1Δ.*

Genotype	uGCR rate*	dGCR rate*	Ratio #
Wild type**	$2.27x10^{-9}$ (1)	$1.97x10^{-8}$ (8.7)	8.7
*mms21-CH***	$2.08x10^{-8}$ (9.1)	$4.2x10^{-6}$ (1850)	202
*sgs1Δ***	$1.69x10^{-8}$ (7.4)	$1.93x10^{-6}$ (850)	114
sgs1Δ mms21-CH	$1.53x10^{-7}$ (67) [$9.58x10^{-8}$ – $8.56x10^{-7}$] {HZY2478/2479}	$2.56x10^{-5}$ (11278) [$1.74x10^{-5}$ – $3.33x10^{-5}$] {HZY2474/2475/2547}	167
*sgs1Δ rad52Δ***	$7.75x10^{-8}$ (34)	$8.07x10^{-8}$ (36)	1.0
sgs1Δ pol32Δ	$2.05x10^{-8}$ (9.0) [$8.75x10^{-9}$ – $3.26x10^{-8}$] {JLY1423/1425}	$3.35x10^{-6}$ (1476) [$2.16x10^{-6}$ – $4.11x10^{-6}$] {JLY1427/1428}	163
sgs1Δ mre11-H125N	$8.07x10^{-7}$ (355) [$7.46x10^{-7}$ – $2.97x10^{-6}$] {JLY1489/1491}	$1.25x10^{-5}$ (5507) [$7.31x10^{-6}$ – $2.36x10^{-5}$] {JLY1490/1493}	15
sgs1-3KR	$2.10x10^{-9}$ (0.9) [$5.50x10^{-10}$ – $3.64x10^{-8}$] {JLY1534/1535}	$9.68x10^{-8}$ (43) [$7.03x10^{-8}$ – $3.54x10^{-7}$] {JLY1537/1538}	46
sgs1-E12G,H13C	$6.51x10^{-8}$ (29) [$4.12x10^{-8}$ – $1.05x10^{-7}$] {JLY1569}	$4.25x10^{-6}$ (1872) [$3.29x10^{-6}$ – $6.89x10^{-6}$] {JLY1572/1573}	65
sgs1-K706A	$2.16x10^{-8}$ (9.5) [$8.91x10^{-9}$ – $3.86x10^{-8}$] {JLY1570/1571}	$2.49x10^{-6}$ (1097) [$2.17x10^{-6}$ –$3.32x10^{-6}$] {JLY1574/1576}	115
exo1Δ	$1.01x10^{-8}$ (4.4) [$5.13x10^{-9}$ – $2.00x10^{-8}$] {HZY2922/3036}	$8.44x10^{-8}$ (37)**	8.4
exo1Δ mms21-CH	$1.29x10^{-7}$ (57) [$8.21x10^{-8}$ – $1.67x10^{-7}$] {HZY3196/3197}	$4.39x10^{-6}$ (1934) [$2.65x10^{-6}$ – $1.02x10^{-5}$] {HZY3190}	34
*pol32Δ***	$3.41x10^{-9}$ (1.5)	$3.15x10^{-8}$ (14)	9.2
pol32Δ mms21-CH	$3.64x10^{-8}$ (16) [$1.24x10^{-8}$ – $1.52x10^{-7}$] {JLY726/727}	$2.75x10^{-7}$ (121) [$1.11x10^{-7}$ – $6.82x10^{-7}$] {JLY768/769}	7.6
srs2Δ	$9.01x10^{-9}$ (4.0) [$5.83x10^{-9}$ – $7.17x10^{-8}$] {JLY1316}	$1.28x10^{-7}$ (56)**	14.2
srs2Δ mms21-CH	$2.03x10^{-7}$ (89) [$1.25x10^{-7}$ – $2.90x10^{-7}$]	$3.41x10^{-6}$ (1502) [$2.59x10^{-6}$ – $4.26x10^{-6}$]	17

	{HZY3286/3287}	{HZY3328/3329}	
pif1Δ**	3.73×10^{-7} (164)	3.61×10^{-7} (159)	1.0
pif1Δ mms21-CH	4.91×10^{-6} (2163)<br>[4.04×10^{-6} – 7.68×10^{-6}]<br>{JLY723/724}	1.21×10^{-5} (5330)<br>[5.56×10^{-6} – 1.93×10^{-5}]<br>{JLY667/668}	2.5
rrm3Δ	1.46×10^{-8} (6.4)<br>[3.33×10^{-9} – 2.00×10^{-7}]<br>{JLY737}	3.87×10^{-8} (17)**	2.7
Genotype	uGCR rate*	dGCR rate*	Ratio #
rrm3Δ mms21-CH	2.37×10^{-8} (10)<br>[4.91×10^{-9} – 8.68×10^{-8}]<br>{JLY773/779}	9.74×10^{-8} (43)<br>[5.77×10^{-8} – 2.28×10^{-7}]<br>{JLY671/672/777}	4.1
mph1Δ**	2.00×10^{-9} (0.9)	1.05×10^{-7} (46)	53
mph1Δ mms21-CH	3.50×10^{-7} (154)<br>[1.47×10^{-7} – 6.72×10^{-7}]<br>{JLY1452/1453}	8.21×10^{-6} (3617)<br>[2.52×10^{-6} – 9.99×10^{-6}]<br>{JLY1456/1457}	23
csm2Δ	1.12×10^{-8} (4.9)<br>[5.30×10^{-9} – 1.96×10^{-8}]<br>{JLY469/470}	7.19×10^{-8} (32)<br>[4.94×10^{-8} – 8.71×10^{-8}]<br>{JLY483/484}	6.4
csm2Δ mms21-CH	2.85×10^{-7} (126)<br>[5.36×10^{-7} – 2.08×10^{-7}]<br>{JLY485}	2.38×10^{-6} (1048)<br>[1.86×10^{-6} – 3.05×10^{-6}]<br>{JLY467/468}	8.4

* Rate of accumulating Can 5-FOA progeny. Number in the parenthesis is the fold increase relative to wild-type uGCR strain.
** Rates taken from (Putnam et al., 2009; 2010; Albuquerque et al., 2013).
# The dGCR rate divided by the uGCR rate.
[ ] represent the upper and lower limits of the 95% confidence intervals of the median.
Strains used to calculate the GCR rate from this study are as indicated using { }.
