# Supplementary material for: SUMO E3 ligase Mms21 prevents spontaneous DNA damage induced genome rearrangements
Source: PLoS Genet. 2018 Mar 5;14(3):e1007250. doi: 10.1371/journal.pgen.1007250 (PMC5860785; doi:10.1371/journal.pgen.1007250)
Supplement: S6 Table — (PDF) [file pgen.1007250.s023.pdf]

S6 Table. Fluctuation results of double mutants containing a null mutation of genes in the DNA damage checkpoint pathways and *mms21-CH.*

Genotype	uGCR rate*	dGCR rate*	Ratio #
Wild type**	$2.27x10^{-9}$ (1) [$1.30x10^{-9}$ – $4.78x10^{-9}$]	$1.97x10^{-8}$ (8.7) [$1.61x10^{-8}$ – $4.32x10^{-8}$]	8.7
*mms21-CH***	$2.08x10^{-8}$ (9.1) [$0.00x10^{-8}$ – $18.2x10^{-8}$]	$4.2x10^{-6}$ (1850) [$2.4x10^{-6}$ – $22.2x10^{-6}$]	202
*mec1Δ sml1Δ***	$2.34x10^{-8}$ (10) [$1.29x10^{-8}$ – $4.02x10^{-8}$]	$1.50x10^{-7}$ (66) [$0.55x10^{-7}$ – $2.74x10^{-7}$]	6.4
mec1Δ sml1Δ mms21-CH	$3.87x10^{-7}$(170) [$8.45x10^{-8}$ – $7.34x10^{-7}$] {HZY2927/2928}	$7.81x10^{-7}$ (344) [$2.20x10^{-7}$ – $2.74x10^{-6}$] {HZY2836/2837}	2.0
*tel1Δ***	$4.99x10^{-9}$ (2.2) [$0.0x10^{-9}$ – $9.20x10^{-9}$]	$2.87x10^{-8}$ (13) [$1.81x10^{-8}$ – $5.68x10^{-8}$]	5.8
tel1Δ mms21-CH	$1.41x10^{-8}$ (6.2) [$8.04x10^{-9}$ – $5.72x10^{-8}$] {HZY3145}	$3.80x10^{-6}$ (1674) [$2.66x10^{-6}$ – $7.67x10^{-6}$] {HZY2974/2975}	270
*rad9Δ***	$2.17x10^{-8}$ (9.6) [$0.98x10^{-8}$ – $4.81x10^{-8}$]	$3.82x10^{-8}$ (17) [$0.0x10^{-8}$ – $9.93x10^{-8}$]	1.8
rad9Δ mms21-CH	$1.14x10^{-7}$ (50) [$2.39x10^{-8}$ – $3.38x10^{-7}$] {HZY3082/3083}	$1.74x10^{-7}$ (77) [$1.40x10^{-7}$ – $2.86x10^{-7}$] {HZY2940/2941}	1.5
*mrc1Δ***	$3.35x10^{-9}$ (1.5) [$0.0x10^{-9}$ – $16.4x10^{-9}$]	$3.75x10^{-7}$ (165) [$2.79x10^{-7}$ – $5.16x10^{-7}$]	112
mrc1Δ mms21-CH	$9.50x10^{-7}$ (419) [$6.05x10^{-8}$ – $1.75x10^{-6}$] {HZY3112/3113}	$7.30x10^{-6}$ (3216) [$4.66x10^{-6}$ – $1.03x10^{-5}$] {HZY2964/2965}	7.7
*mrc1-aq***	$1.51x10^{-9}$ (0.7) [$0.0x10^{-9}$ – $5.19x10^{-9}$]	$1.23x10^{-7}$ (54) [$0.64x10^{-7}$ – $5.29x10^{-7}$]	81
mrc1-aq mms21-CH	$1.06x10^{-7}$ (47) [$7.52x10^{-8}$ – $1.89x10^{-7}$] {JLY1460/1461}	$4.60x10^{-6}$ (2026) [$3.18x10^{-6}$ – $7.06x10^{-6}$] {JLY1464/1465}	43
*rad53Δ sml1Δ***	$5.60x10^{-8}$ (25) [$2.53x10^{-8}$ – $11.0x10^{-8}$]	$3.05x10^{-7}$ (134) [$1.16x10^{-7}$ – $7.31x10^{-7}$]	5.4
rad53Δ sml1Δ mms21-CH	$7.19x10^{-8}$ (32) [$2.89x10^{-8}$ – $9.05x10^{-8}$] {HZY3008}	$5.48x10^{-7}$ (241) [$2.73x10^{-7}$ – $9.80x10^{-7}$] {HZY2805/3236}	7.6
*chk1Δ***	$1.76x10^{-8}$ (7.8) [$7.5x10^{-10}$ – $6.02x10^{-8}$]	$1.96x10^{-7}$ (86) [$0.86x10^{-7}$ – $4.37x10^{-7}$]	11
chk1Δ mms21-CH	$6.75x10^{-8}$ (30) [$3.99x10^{-6}$ – $9.75x10^{-6}$] {HZY3158}	$4.02x10^{-6}$ (1771) [$3.02x10^{-6}$ – $5.93x10^{-6}$] {HZY2987/2988}	60

Genotype	uGCR rate*	dGCR rate*	Ratio #
dun1Δ	1.26×10^{-8} (5.6) [7.70×10^{-9} – 2.25×10^{-8}] {HZY2876/2877}	5.52×10^{-7} (243) [4.13×10^{-7} – 1.04×10^{-6}] {HZY2864/2865}	44
dun1Δ mms21-CH	5.96×10^{-7} (263) [4.91×10^{-8} – 6.01×10^{-6}] {HZY3154}	3.39×10^{-6} (1493) [2.51×10^{-6} – 5.76×10^{-6}] {HZY2839/2995}	5.7
mec3Δ	1.02×10^{-8} (4.5) [2.56×10^{-9} – 6.33×10^{-8}] {HZY3088}	3.77×10^{-7} (166) [2.29×10^{-7} – 7.37×10^{-7}] {HZY2632/2633}	37
mec3Δ mms21-CH	1.01×10^{-7} (44) [4.74×10^{-8} – 2.14×10^{-7}] {HZY3092}	6.15×10^{-7} (271) [4.13×10^{-7} – 8.78×10^{-7}] {HZY2634/2635}	6.1
*rad24Δ***	2.00×10^{-8} (8.8) [0.99×10^{-8} – 3.06×10^{-8}]	1.97×10^{-7} (87) [1.43×10^{-7} – 2.52×10^{-7}]	9.9
rad24Δ mms21-CH	1.68×10^{-7} (74) [5.41×10^{-8} – 5.29×10^{-7}] {HZY3103/3104}	1.84×10^{-6} (811) [9.73×10^{-7} – 3.87×10^{-6}] {HZY2698/2699}	11

* Rate of accumulating Can 5-FOA progeny. Number in the parenthesis is the fold increase relative to wild-type uGCR strain.
** Rates taken from (Putnam et al., 2009; 2010; Albuquerque et al., 2013).
# The dGCR rate divided by the uGCR rate.
Square brackets represent the upper and lower limits of the 95% confidence intervals of the median.
Strains used to calculate the GCR rate from this study are as indicated using curly braces.
