# Supplementary material for: SUMO E3 ligase Mms21 prevents spontaneous DNA damage induced genome rearrangements
Source: PLoS Genet. 2018 Mar 5;14(3):e1007250. doi: 10.1371/journal.pgen.1007250 (PMC5860785; doi:10.1371/journal.pgen.1007250)
Supplement: S7 Table — (DOCX) [file pgen.1007250.s024.docx]

**S7 Table.** *S. cerevisiae* strains

| ***Strain*** | ***Genotype**** | ***Reference*** |
| --- | --- | --- |
| RDKY6677 | **MATa** *iYEL072W::hph can1::hisG yel068c::CAN1/URA3 leu2Δ1 trp1Δ63 his3Δ200 lys2ΔBgl hom3-10 ade2Δ1 ade8 ura3-52* | Putnam et al. 2009 |
| RDKY6686 | RDKY6677 *mre11::HIS3* | Putnam et al. 2009 |
| HZY2181 | RDKY6677 *cir0* | Albuquerque et al. 2013 |
| JLY469 | HZY2181 *csm2Δ::natMX4* | This study |
| HZY2876 | HZY2181 *dun1Δ::HIS3* | This study |
| HZY3036 | HZY2181 *exo1Δ::natMX4* | This study |
| HZY3088 | HZY2181 *mec3Δ::HIS3* | This study |
| HZY2173 | HZY2181 *mms21-CH::kanMX6* | Albuquerque et al. 2013 |
| HZY2199 | HZY2181 *mms21-CH::kanMX6* | Albuquerque et al. 2013 |
| HZY3197 | HZY2181 *mms21-CH::kanMX6 exo1Δ::natMX4* | This study |
| HZY2927 | HZY2181 *mms21-CH::kanMX6 mec1Δ::HIS3 sml1Δ::TRP1* | This study |
| HZY3092 | HZY2181 *mms21-CH::kanMX6* *mec3Δ::HIS3* | This study |
| JLY1452 | HZY2181 *mms21-CH::kanMX6 mph1::natMX4* | This study |
| HZY3112 | HZY2181 *mms21-CH::kanMX6 mrc1Δ::HIS3* | This study |
| JLY1460 | HZY2181 *mms21-CH::kanMX6* *mrc1-AQ::TRP1* | This study |
| HZY2480 | HZY2181 *mms21-CH::kanMX6 mre11Δ::natMX4* | This study |
| JLY930 | HZY2181 *mms21-CH::kanMX6 mre11-H125N::TRP1* | This study |
| JLY723 | HZY2181 *mms21-CH::kanMX6 pif1Δ::HIS3* | This study |
| JLY726 | HZY2181 *mms21-CH::kanMX6 pol32Δ::HIS3* | This study |
| JLY727 | HZY2181 *mms21-CH::kanMX6 pol32Δ::HIS3* | This study |
| HZY3082 | HZY2181 *mms21-CH::kanMX6 rad9Δ::HIS3* | This study |
| HZY3103 | HZY2181 *mms21-CH::kanMX6* *rad24Δ::HIS3* | This study |
| HZY3265 | HZY2181 *mms21-CH::kanMX6 rad51Δ::HIS3* | This study |
| HZY3310 | HZY2181 *mms21-CH::kanMX6 rad52Δ::HIS3* | This study |
| HZY3271 | HZY2181 *mms21-CH::kanMX6 rad54Δ::HIS3* | This study |
| HZY3245 | HZY2181 *mms21-CH::kanMX6* *rad55Δ::HIS3* | This study |
| HZY3362 | HZY2181 *mms21-CH::kanMX6 rad59Δ::HIS3* | This study |
| HZY3280 | HZY2181 *mms21-CH::kanMX6 rdh54Δ::HIS3* | This study |
| JLY779 | HZY2181 *mms21-CH::kanMX6 rrm3Δ::natMX4* | This study |
| HZY2599 | HZY2181 *mms21-CH::kanMX6 sae2Δ::TRP1* | This study |
| HZY2478 | HZY2181 *mms21-CH::kanMX6 sgs1Δ::natMX4* | This study |
| HZY3286 | HZY2181 *mms21-CH::kanMX6 srs2Δ::natMX4* | This study |
| HZY2771 | HZY2181 *mre11-H125N::TRP1* | This study |
| HZY3269 | HZY2181 *rad54Δ::HIS3* | This study |
| HZY3270 | HZY2181 *rad54Δ::HIS3* | This study |
| JLY406 | HZY2181 *rad54Δ::kanMX6* | This study |
| JLY382 | HZY2181 *rdh54Δ::HIS3* | This study |
| JLY737 | HZY2181 *rrm3Δ::natMX4* | This study |
| JLY1534 | HZY2181 *sgs1-3KR::HIS3* | This study |
| JLY1535 | HZY2181 *sgs1-3KR::HIS3* | This study |
| JLY1489 | HZY2181 *sgs1∆::kanMX6 mre11-H125N::TRP1* | This study |
| JLY1425 | HZY2181 *sgs1Δ::kanMX6 pol32∆::natMX4* | This study |
| JLY1316 | HZY2181 *srs2Δ::TRP1* | This study |
| JLY1569 | HZY2181 *sgs1-E12G,H13C::HIS3* | This study |
| JLY1570 | HZY2181 *sgs1-K706A::HIS3* | This study |
| JLY1571 | HZY2181 *sgs1-K706A::HIS3* | This study |
| JLY470 | HZY2181 **MATalpha** *csm2Δ::natMX4* | This study |
| HZY2877 | HZY2181 **MATalpha** *dun1Δ::HIS3* | This study |
| HZY2922 | HZY2181 **MATalpha** *exo1Δ::natMX4* | This study |
| HZY3158 | HZY2181 **MATalpha** *mms21-CH::kanMX6* *chk1Δ::HIS3* | This study |
| JLY485 | HZY2181 **MATalpha** *mms21-CH::kanMX6* *csm2Δ::natMX4* | This study |
| HZY3154 | HZY2181 **MATalpha** *mms21-CH::kanMX6 dun1Δ::HIS3* | This study |
| HZY3196 | HZY2181 **MATalpha** *mms21-CH::kanMX6 exo1Δ::natMX4* | This study |
| HZY2928 | HZY2181 **MATalpha** *mms21-CH::kanMX6* *mec1Δ::HIS3 sml1Δ::TRP1* | This study |
| JLY1453 | HZY2181 **MATalpha** *mms21-CH::kanMX6 mph1Δ::natMX4* | This study |
| HZY3113 | HZY2181 **MATalpha** *mms21-CH::kanMX6 mrc1Δ::HIS3* | This study |
| JLY1461 | HZY2181 **MATalpha** *mms21-CH::kanMX6 mrc1-AQ::TRP1* | This study |
| HZY2481 | HZY2181 **MATalpha** *mms21-CH::kanMX6 mre11Δ::natMX4* | This study |
| JLY929 | HZY2181 **MATalpha** *mms21-CH::kanMX6 mre11-H125N::TRP1* | This study |
| JLY724 | HZY2181 **MATalpha** *mms21-CH::kanMX6 pif1Δ::HIS3* | This study |
| HZY3083 | HZY2181 **MATalpha** *mms21-CH::kanMX6 rad9Δ::HIS3* | This study |
| HZY3104 | HZY2181 **MATalpha** *mms21-CH::kanMX6* *rad24Δ::HIS3* | This study |
| HZY3266 | HZY2181 **MATalpha** *mms21-CH::kanMX6 rad51Δ::HIS3* | This study |
| HZY3311 | HZY2181 **MATalpha** *mms21-CH::kanMX6 rad52Δ::HIS3* | This study |
| HZY3008 | HZY2181 **MATalpha** *mms21-CH::kanMX6* *rad53Δ::HIS3 sml1Δ::TRP1* | This study |
| HZY3272 | HZY2181 **MATalpha** *mms21-CH::kanMX6 rad54Δ::HIS3* | This study |
| HZY3246 | HZY2181 **MATalpha** *mms21-CH::kanMX6 rad55Δ::HIS3* | This study |
| HZY3363 | HZY2181 **MATalpha** *mms21-CH::kanMX6* *rad59Δ::HIS3* | This study |
| HZY3281 | HZY2181 **MATalpha** *mms21-CH::kanMX6 rdh54Δ::HIS3* | This study |
| JLY773 | HZY2181 **MATalpha** *mms21-CH::kanMX6 rrm3Δ::natMX4* | This study |
| HZY2600 | HZY2181 **MATalpha** *mms21-CH::kanMX6 sae2Δ::TRP1* | This study |
| HZY2479 | HZY2181 **MATalpha** *mms21-CH::kanMX6 sgs1Δ::natMX4* | This study |
| HZY3287 | HZY2181 **MATalpha** *mms21-CH::kanMX6* *srs2Δ::natMX4* | This study |
| HZY3145 | HZY2181 **MATalpha** *mms21-CH::kanMX6* *tel1Δ::HIS3* | This study |
| HZY2772 | HZY2181 **MATalpha** *mre11-H125N::TRP1* | This study |
| HZY3264 | HZY2181 **MATalpha** *rad51Δ::HIS3* | This study |
| HZY3369 | HZY2181 **MATalpha** *rad55Δ::HIS3* | This study |
| HZY3277 | HZY2181 **MATalpha** *rdh54Δ::HIS3* | This study |
| JLY1491 | HZY2181 **MATalpha** *sgs1∆::kanMX6 mre11-H125N::TRP1* | This study |
| JLY1423 | HZY2181 **MATalpha** *sgs1Δ::kanMX6* *pol32∆::natMX4* | This study |
| RDKY6678 | **MATa** *iYEL072W::hph can1::hisG yel072w::CAN1/URA3 leu2Δ1 trp1Δ63 his3Δ200 lys2ΔBgl hom3-10 ade2Δ1 ade8 ura3-52* | Putnam et al. 2009 |
| HZY2131 | RDKY6678 *cir0* | Albuquerque et al. 2013 |
| JLY483 | HZY2131 *csm2Δ::natMX4* | This study |
| HZY2864 | HZY2131 *dun1Δ::HIS3* | This study |
| HZY2632 | HZY2131 *mec3Δ::HIS3* | This study |
| HZY3299 | HZY2131 *rad54Δ::HIS3* | This study |
| HZY2145 | HZY2131 *mms21-CH::kanMX6* | Albuquerque et al. 2013 |
| HZY2146 | HZY2131 *mms21-CH::kanMX6* | Albuquerque et al. 2013 |
| HZY2987 | HZY2131 *mms21-CH::kanMX6* *chk1Δ::HIS3* | This study |
| HZY2988 | HZY2131 *mms21-CH::kanMX6* *chk1Δ::HIS3* | This study |
| JLY468 | HZY2131 *mms21-CH::kanMX6 csm2Δ::natMX4* | This study |
| HZY2839 | HZY2131 *mms21-CH::kanMX6 dun1Δ::HIS3* | This study |
| HZY2995 | HZY2131 *mms21-CH::kanMX6 dun1Δ::HIS3* | This study |
| HZY3190 | HZY2131 *mms21-CH::kanMX6 exo1Δ::natMX4* | This study |
| HZY2836 | HZY2131 *mms21-CH::kanMX6 mec1Δ::HIS3 sml1Δ::TRP1* | This study |
| HZY2634 | HZY2131 *mms21-CH::kanMX6* *mec3Δ::HIS3* | This study |
| HZY2964 | HZY2131 *mms21-CH::kanMX6 mrc1Δ::HIS3* | This study |
| JLY1465 | HZY2131 *mms21-CH::kanMX6 mrc1-AQ::TRP1* | This study |
| HZY2476 | HZY2131 *mms21-CH::kanMX6 mre11Δ::natMX4* | This study |
| HZY2516 | HZY2131 *mms21-CH::kanMX6 mre11Δ::natMX4* | This study |
| JLY974 | HZY2131 *mms21-CH::kanMX6 mre11-H125N::TRP1* | This study |
| JLY975 | HZY2131 *mms21-CH::kanMX6* *mre11-H125N::TRP1* | This study |
| JLY668 | HZY2131 *mms21-CH::kanMX6 pif1Δ::HIS3* | This study |
| JLY769 | HZY2131 *mms21-CH::kanMX6 pol32Δ::natMX4* | This study |
| HZY2940 | HZY2131 *mms21-CH::kanMX6* *rad9Δ::HIS3* | This study |
| HZY2698 | HZY2131 *mms21-CH::kanMX6* *rad24Δ::TRP1* | This study |
| HZY3257 | HZY2131 *mms21-CH::kanMX6 rad51Δ::HIS3* | This study |
| HZY3019 | HZY2131 *mms21-CH::kanMX6 rad52Δ::HIS3* | This study |
| HZY2805 | HZY2131 *mms21-CH::kanMX6 rad53Δ::HIS3 sml1Δ::TRP1* | This study |
| HZY3236 | HZY2131 *mms21-CH::kanMX6 rad53Δ::HIS3 sml1Δ::TRP1* | This study |
| HZY3300 | HZY2131 *mms21-CH::kanMX6 rad54Δ::HIS3* | This study |
| HZY3025 | HZY2131 *mms21-CH::kanMX6 rad55Δ::HIS3* | This study |
| HZY3166 | HZY2131 *mms21-CH::kanMX6 rad59Δ::HIS3* | This study |
| HZY3275 | HZY2131 *mms21-CH::kanMX6 rdh54Δ::HIS3* | This study |
| JLY672 | HZY2131 *mms21-CH::kanMX6 rrm3Δ::HIS3* | This study |
| HZY2591 | HZY2131 *mms21-CH::kanMX6 sae2Δ::TRP1* | This study |
| HZY2474 | HZY2131 *mms21-CH::kanMX6 sgs1Δ::natMX4* | This study |
| HZY2547 | HZY2131 *mms21-CH::kanMX6 sgs1Δ::natMX4* | This study |
| HZY3328 | HZY2131 *mms21-CH::kanMX6 srs2Δ::natMX4* | This study |
| HZY2974 | HZY2131 *mms21-CH::kanMX6* *tel1Δ::HIS3* | This study |
| HZY2763 | HZY2131 *mre11-H125N::TRP1* | This study |
| HZY3021 | HZY2131 *rad55Δ::HIS3* | This study |
| JLY1537 | HZY2131 *sgs1-3KR::HIS3* | This study |
| JLY1538 | HZY2131 *sgs1-3KR::HIS3* | This study |
| JLY1493 | HZY2131 *sgs1∆::kanMX6 mre11-H125N::TRP1* | This study |
| JLY1572 | HZY2131 *sgs1-E12G,H13C::HIS3* | This study |
| JLY1573 | HZY2131 *sgs1-E12G,H13C::HIS3* | This study |
| JLY1574 | HZY2131 *sgs1-K706A::HIS3* | This study |
| JLY1576 | HZY2131 *sgs1-K706A::HIS3* | This study |
| JLY484 | HZY2131 **MATalpha** *csm2Δ::natMX4* | This study |
| HZY2865 | HZY2131 **MATalpha** *dun1Δ::HIS3* | This study |
| JLY467 | HZY2131 **MATalpha** *mms21-CH::kanMX6 csm2Δ::natMX4* | This study |
| HZY2633 | HZY2131 **MATalpha** *mec3Δ::HIS3* | This study |
| HZY2837 | HZY2131 **MATalpha** *mms21-CH::kanMX6* *mec1Δ::HIS3 sml1Δ::TRP1* | This study |
| HZY2635 | HZY2131 **MATalpha** *mms21-CH::kanMX6* *mec3Δ::HIS3* | This study |
| JLY1456 | HZY2131 **MATalpha** *mms21-CH::kanMX6 mph1Δ::natMX4* | This study |
| JLY1457 | HZY2131 **MATalpha** *mms21-CH::kanMX6 mph1Δ::natMX4* | This study |
| HZY2965 | HZY2131 **MATalpha** *mms21-CH::kanMX6 mrc1Δ::HIS3* | This study |
| JLY1464 | HZY2131 **MATalpha** *mms21-CH::kanMX6* *mrc1-AQ::TRP1* | This study |
| HZY2477 | HZY2131 **MATalpha** *mms21-CH::kanMX6 mre11Δ::natMX4* | This study |
| JLY973 | HZY2131 **MATalpha** *mms21-CH::kanMX6* *mre11-H125N::TRP1* | This study |
| JLY667 | HZY2131 **MATalpha** *mms21-CH::kanMX6 pif1Δ::HIS3* | This study |
| JLY768 | HZY2131 **MATalpha** *mms21-CH::kanMX6* *pol32Δ::natMX4* | This study |
| HZY2941 | HZY2131 **MATalpha** *mms21-CH::kanMX6 rad9Δ::HIS3* | This study |
| HZY2699 | HZY2131 **MATalpha** *mms21-CH::kanMX6 rad24Δ::TRP1* | This study |
| JLY380 | HZY2131 **MATalpha** *mms21-CH::kanMX6 rad51Δ::HIS3* | This study |
| HZY3258 | HZY2131 **MATalpha** *mms21-CH::kanMX6 rad51Δ::HIS3* | This study |
| HZY3020 | HZY2131 **MATalpha** *mms21-CH::kanMX6* *rad52Δ::HIS3* | This study |
| HZY3301 | HZY2131 **MATalpha** *mms21-CH::kanMX6 rad54Δ::HIS3* | This study |
| HZY3026 | HZY2131 **MATalpha** *mms21-CH::kanMX6 rad55Δ::HIS3* | This study |
| HZY3167 | HZY2131 **MATalpha** *mms21-CH::kanMX6 rad59Δ::HIS3* | This study |
| HZY3276 | HZY2131 **MATalpha** *mms21-CH::kanMX6 rdh54Δ::HIS3* | This study |
| JLY444 | HZY2131 **MATalpha** *mms21-CH::kanMX6 rdh54Δ::HIS3* | This study |
| JLY671 | HZY2131 **MATalpha** *mms21-CH::kanMX6 rrm3Δ::HIS3* | This study |
| JLY777 | HZY2131 **MATalpha** *mms21-CH::kanMX6* *rrm3Δ::natMX4* | This study |
| HZY2592 | HZY2131 **MATalpha** *mms21-CH::kanMX6 sae2Δ::TRP1* | This study |
| HZY2475 | HZY2131 **MATalpha** *mms21-CH::kanMX6 sgs1Δ::natMX4* | This study |
| HZY3329 | HZY2131 **MATalpha** *mms21-CH::kanMX6* *srs2Δ::natMX4* | This study |
| HZY2975 | HZY2131 **MATalpha** *mms21-CH::kanMX6* *tel1Δ::HIS3* | This study |
| HZY2764 | HZY2131 **MATalpha** *mre11-H125N::TRP1* | This study |
| HZY3306 | HZY2131 **MATalpha** *rad54Δ::HIS3* | This study |
| HZY3368 | HZY2131 **MATalpha** *rad55Δ::HIS3* | This study |
| HZY3274 | HZY2131 **MATalpha** *rdh54Δ::HIS3* | This study |
| JLY442 | HZY2131 **MATalpha** *rdh54Δ::HIS3* | This study |
| JLY1490 | HZY2131 **MATalpha** *sgs1∆::kanMX6 mre11-H125N::TRP1* | This study |
| JLY1427 | HZY2131 **MATalpha** *sgs1Δ::kanMX6 pol32∆::natMX4* | This study |
| JLY1428 | HZY2131 **MATalpha** *sgs1Δ::kanMX6* *pol32∆::natMX4* | This study |
| RDKY8934 | **MATalpha** *trp1∆63 ura3∆0 leu2∆1 his3∆200 lys2∆Bgl hom3-10 ade2∆1 ade8 can1 cyh2-Q38K iYFR016C::PMFA1-LEU2 DDC2-EGFP::HIS3MX6 NUP49-mCherry::hphNT1* | This study |
| JLY1484 | RDKY8934 *MMS21* | This study |
| JLY1485 | RDKY8934 *MMS21* | This study |
| JLY1481 | RDKY8934 *mms21-CH::kanMX6* | This study |
| JLY1486 | RDKY8934 *mms21-CH::kanMX6* | This study |
| RDKY8936 | **MATalpha** *trp1∆63 ura3∆0 leu2∆1 his3∆200 lys2∆Bgl hom3-10 ade2∆1 ade8 can1 cyh2-Q38K iYFR016C::PMFA1-LEU2 RAD52-EGFP::HIS3MX6 NUP49-mCherry::hphNT1* | This study |
| JLY1548 | RDKY8936 *mms21-CH::kanMX6* | This study |
| JLY1549 | **MATa** RDKY8936 *mms21-CH::kanMX6* | This study |
| LSY2202-15D | **MATa** *ade2-n his3::NATMX4 met22::KIURA3 can1-100 his3-11,15 leu2-3,112 trp1-1 ura3-1 RAD5+* | Ho et al. 2010 |
| LSY2165-71B | **MATalpha** *ade2-ISceIcs his3::hphMX4 can1-100 his3-11,15 leu2-3,112 trp1-1 ura3-1 RAD5+* | Ho et al. 2010 |
| JLY1566 | **MATa**/**MATalpha** *ade2-n /ade2-ISceIcs his3::NATMX4/his3-11,15 met22::KIURA3/met22 can1-100/can1-100 leu2-3,112/leu2-3,112 trp1-1/trp1-1 ura3-1/ura3-1 RAD5+/RAD5+* | This study |
| JLY1568 | JLY1566 *mms21-CH::KanMX6/mms21-CH::KanMX6* | This study |

**cir0* are strains lacking the 2-micron plasmid (see Methods). The *mms21-CH* mutation encodes the Mms21-C200A,H202A variant protein. The *sgs1-3KR* mutation encodes the Sgs1-K175R,K621R,K831R variant protein.
