# Supplementary material for: SUMO E3 ligase Mms21 prevents spontaneous DNA damage induced genome rearrangements
Source: PLoS Genet. 2018 Mar 5;14(3):e1007250. doi: 10.1371/journal.pgen.1007250 (PMC5860785; doi:10.1371/journal.pgen.1007250)
Supplement: S10 Fig — A mechanism that can explain the formation of the inverted duplication junctions observed here (A and S11 Fig). A. An example of an inversion junction sequence identified in mms21-CH mre11Δ GCR isolate bzg013 is displayed with the inversion junction in center and the sequence alignments to chromosome V in opposite orientation above and below. B. The inversion junction can be formed by 5’ resection from a double-stranded break (DSB) to generate a 3’-overhang. Intramolecular loop formation mediated by intra-strand base pairing generates a 3’ primer terminus that can be extended by DNA polymerases. This initial hairpin-capped chromosome will generate a dicentric chromosome upon replication, which is unstable and undergoes additional rounds of rearrangement. Note that if a nuclease like Rad1-Rad10 is present to cleave flap-containing DNA molecules [73], then the double-strand break need to not occur at the end of the homology, but rather an annealed primer terminus can be generated by flap cleavage, analogously to the cleavage of 3’ non-homologous tails during homologous recombination [74]. (PDF) [file pgen.1007250.s010.pdf]

S10 Figure

A. **Bzg013 (*mms21-CH mre11*)**
Mapped junction sequence

```
                                    :ChrV 42277+
                                    :CCCGGGACCCCGGG:CATAAGTGCATAGTAGGCACAGACCTGA
                                     |||||||||||||| |||||||||||||||||||||||||||||
GACAGATCAGGTCTGTGCCTACTATGCACTTATG:CCCGGGACCCCGGG:CATAAGTGCATAGTAGGCACAGACCTGA
|||||||||||||||||||||||||||||||||| |||||| |||||||
GACAGATCAGGTCTGTGCCTACTATGCACTTATG:CCCGGGGTCCCGGG:
                                     ChrV 42277-:
```

B. **Potential inversion formation mechanism**

DSB

```
5’-CCCGGGACCCCGGGCATAAGTGCATAGTAGGCACAGACCTGATCTGTCGGGTGCAGTTTA...-3’
   ||||||||||||||||||||||||||||||||||||||||||||||||||||||||||||
3’-GGGCCCTGGGGCCCGTATTCACGTATCATCCGTGTCTGGACTAGACAGCCCACGTCAAAT...-5’
```

5’ resection to generate 3’ overhang

```
                                          5’-TGATCTGTCGGGTGCAGTTTA...-3’
                                             |||||||||||||||||||||
3’-GGGCCCTGGGGCCCGTATTCACGTATCATCCGTGTCTGGACTAGACAGCCCACGTCAAAT...-5’
```

intramolecular loop formation

```
            T CCCGGG-3’
2 base loop   ||||||
            G GGGCCCGTATTCACGTATCATCCGTGTCTGGACTAGACAGCCCACGTCAAAT...-5’
```

DNA replication

```
            T CCCGGGCATAAGTGCATAGTAGGCACAGACCTGATCTGTCGGGTGCAGTTTA-3’
2 base loop   ||||||||||||||||||||||||||||||||||||||||||||||||||||
            G GGGCCCGTATTCACGTATCATCCGTGTCTGGACTAGACAGCCCACGTCAAAT...-5’
```
